# SECmeres outperform extracellular vesicles as potential blood RNA biomarkers for Alzheimer’s disease

**DOI:** 10.1038/s41467-026-74541-8

**Published:** 2026-06-22

**Authors:** Edgar Gonzalez-Kozlova, Swapnil Tichkule, Yohei Nose, Tzu-Yi Chen, Eduard Reznik, Juliet V. Santiago, Anish Korrapati, Taliah Soleymani, Roman Kosoy, Igor Figueiredo, Donghoon Lee, Gabriel E. Hoffman, Natasha Kyprianou, Ronald E. Gordon, Carlos Cordon-Cardo, Srikant Rangaraju, Nicholas T. Seyfried, Vahram Haroutunian, John F. Fullard, Panos Roussos, Navneet Dogra

**Affiliations:** 1 Department of Immunology, Icahn School of Medicine at Mount Sinai, New York, NY USA; 2 Department of Psychiatry, Icahn School of Medicine at Mount Sinai, New York, NY USA; 3 Department of Pathology, Molecular and Cell-Based Medicine, Icahn School of Medicine at Mount Sinai, New York, NY USA; 4 Department of Urology, Icahn School of Medicine at Mount Sinai, New York, NY USA; 5 Department of Biochemistry and Neurology, Emory University, Atlanta, GA USA; 6 Department of Genetics and Genomics Sciences, Icahn School of Medicine at Mount Sinai, New York, NY USA; 7 Center for Disease Neurogenomics, Icahn School of Medicine at Mount Sinai, New York, NY USA; 8 Department of Neurology, Yale University, New Haven, CT USA; 9 Center for Precision Medicine and Translational Therapeutics, James J. Peters VA Medical Center, Bronx, NY USA; 10 Mental Illness Research, Education, and Clinical Center (VISN 2 South), James J. Peters VA Medical Center, Bronx, NY USA; 11 Icahn Genomics Institute, Icahn School of Medicine at Mount Sinai, New York, NY USA; 12 Artificial Intelligence and Human Health, Icahn School of Medicine at Mount Sinai, New York, NY USA; 13 Alzheimer’s Disease Research Center, Icahn School of Medicine at Mount Sinai, New York, NY USA

**Keywords:** Alzheimer's disease, Alzheimer's disease

## Abstract

Cells release heterogeneous extracellular vesicles and particles (EVPs) into circulation, carrying RNA and proteins that reflect their origin. Recently, brain-derived EVs have gained significant attention as non-invasive biomarkers for Alzheimer’s disease (AD). Here, we identified sub-50nm extracellular nanoparticles in human brain and blood that lack the hallmarks of small EVs, exosomes, exomeres, and supermeres but are enriched for brain-specific markers, hereafter termed small EPs or ‘SECmeres’. We discovered that RNAs associated with SECmeres discriminated AD cases from controls with higher significance than small EVs, large EVs showed no differences. Discriminating RNAs were enriched in small EVs (Synaptotagmin, Alpha-synuclein, MAPT) or SECmeres (L1CAM, Syntaxin, Neurogranin), indicating distinct brain-derived signatures. Single-cell RNAseq deconvolution shows small EVs contain RNAs from diverse brain cells, whereas SECmeres enrich brain endothelial transcripts, lining cerebral blood vessels and forming the blood–brain barrier (BBB). These findings challenge the prevailing view that small EVs are the primary carriers of biomarkers. Collectively, our study shows that blood EVPs carry brain-specific information for liquid biopsy, pending validation in larger blinded clinical trials.

## Abstract (web summary)

Extracellular vesicles (EVs) can help diagnose diseases. Here, the authors show that brain-derived RNAs associated with SECmeres, particles smaller than EVs, in the blood can identify cases of Alzheimer’s disease, offering a potential biomarker.

## Introduction

Alzheimer’s disease (AD) is a neurodegenerative disorder that can only be definitively diagnosed through pathological examination of post-mortem brain tissue^1,2^. Blood-based RNA biomarkers provide a minimally invasive approach that is amenable to repeated sampling and longitudinal studies, enabling the capture of diverse disease-related processes, including neuronal, glial, and immune alterations^3^. Extracellular vesicles (EVs) are heterogeneous entities in circulation that carry molecular signatures from their cell of origin, such as proteins^4^ and RNA^5^, with potential applications as non-invasive diagnostic biomarkers^6,7^. Consequently, brain-derived EVs have emerged as critical biomarkers, mediators of intercellular communication^8–10^, and mechanisms for molecular clearance among neurons, glia, and other cell lineages of the central nervous system^11–14^. Recently, blood and cerebrospinal fluid (CSF) derived EVs from subjects with AD were reported to exhibit changes in phosphorylated(p) tau, APoE4, Aβ42, and RNAs^3,11,15–17^. Furthermore, growing clinical and commercial interest is evident, with over 200 trials (clinicaltrials.gov) and 15 ventures (sec.gov) launched using the technical terms “*exosomes*” or “*extracellular vesicles*”^18^.

Although significant progress has been made in understanding the biology, function, and translational potential of small EVs’and “exosomes”, other heterogeneous extracellular vesicles and particles (EVPs) remain poorly characterized, representing a major gap in our knowledge^19,20^. Notably, recent studies, ongoing clinical trials, and commercial ventures have focused on isolating ~100–200 nm subpopulations that are positive for hallmarks of small EVs^18,21^. As such, other potentially important particles are often discarded as debris. However, given recent studies showing that sub-50 nm particles are of importance in cancer^22–25^, a comprehensive characterization of the diverse EVP subpopulations from human brain and blood is overdue. This is, in part, due to a lack of technologies to efficiently and reproducibly isolate heterogeneous EVPs from different biofluids^26–30^. Furthermore, relatively little attention has been paid to characterizing the RNA associated with sub-50 nm particles from human brain and blood, especially from AD and non-AD subjects.

While this study builds on our previous work^4–6,31,32^, we expand our investigation to a broader range of EVPs beyond the traditionally studied small EVs and exosomes. During the isolation of small EVs, we discovered a novel set of particles in the brain microenvironment and matched blood. These nanoparticles are smaller (<50 nm), negative for the hallmarks of small EVs^21,33^, exosomes, exomeres^25^, and supermeres^21,24^, and are enriched with brain-specific proteins and RNAs. We have termed these particles as “EPs” or “SECmeres”. Here, we asked whether RNAs associated with different EVPs can discriminate AD from non-AD subjects. To identify high-confidence RNA signatures associated with definitive AD pathology, we isolated and characterized EVP subpopulations (large EVs, small EVs, and small EP) from post-mortem brains (*n* = 26) and blood (*n* = 26) of neuropathologically confirmed AD (*n* = 10) and non-AD (*n* = 16) subjects (total EVPs *n* = 140). We then generated and compared comprehensive proteomic and transcriptomic datasets of diverse EVPs. To identify their brain cell of origin, we conducted deconvolution of EVP specific signatures using single-nucleus RNA sequencing (snRNA-seq) data from specimens (*n* = 1494) of human dorsolateral prefrontal cortex (DLPFC) generated in our lab^34,35^. Finally, we evaluated the diagnostic potential of EVP-RNAs as liquid biopsies for AD. To ensure that our RNA discovery is robust, unbiased, and reproducible across institutions, we analyzed additional EVP RNA-seq from human brain homogenates and iPSC neurons (total combined samples 62, with 40 AD and 22 controls)^36,37^. We discovered that RNAs associated with small EVs, and SECmeres differentiated neuropathologically confirmed AD cases from controls. The RNA species that enabled this discrimination were uniquely enriched in either small EVs (Synaptotagmin, MAPT) or SECmeres (L1CAM, syntaxin-1B, MBP), suggesting distinct AD signatures within these subpopulations. Collectively, our study provides a proof-of-concept that the transcriptome of peripheral blood EVPs carries disease-specific information in AD with potential applications as a minimally invasive diagnostic tool.

## Results

### Rigorous and reproducible isolation of brain and blood-derived EVPs (large EVs, small EVs, SECmeres)

**Fig. 1:**
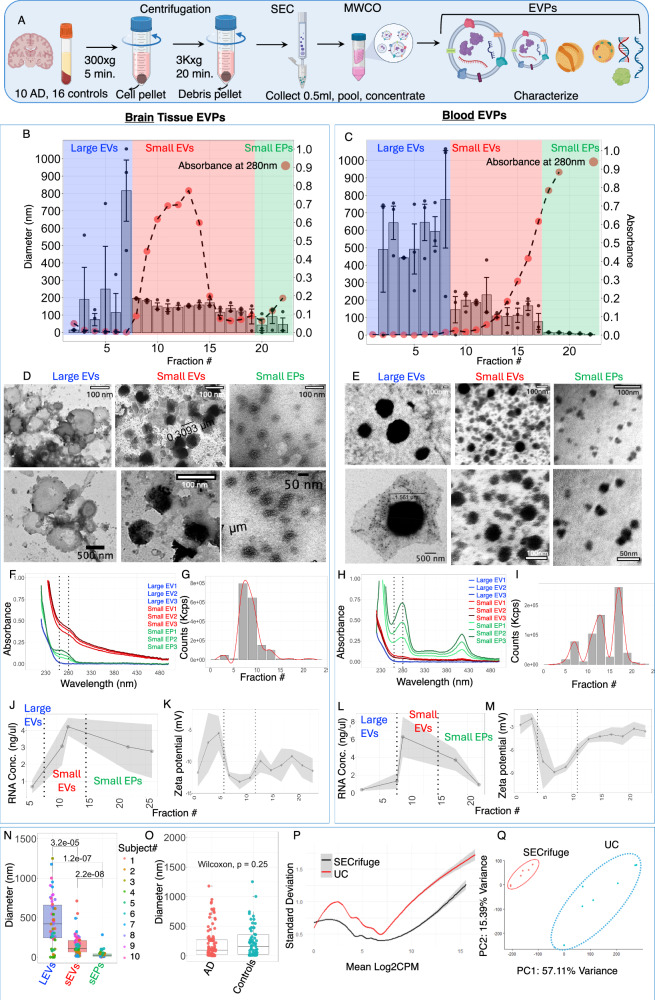
Characterization of brain and blood derived EVPs. **A** Schematic drawing of integrative *SECrifuge* methodology used in this study to isolate diverse EVPs from brain and blood (described in detail in Supplementary Fig. 1). Created in BioRender. Dogra, N. (2026) https://BioRender.com/9c4uw9y. EVPs isolated from brain (**B**) and blood (**C**). **D**, **E** Transmission electron microscopy (TEM) revealing distinct morphologies and sizes of EVPs from brain and blood, respectively. All scale bars for top panels for **D**, **E** are 100 nm. scale bars for lower panels are 500 nm, 100 nm, and 50 nm for large EVs, small EVs, and small EPs (SECmeres), respectively. Unprocessed TEMs are provided in Supplementary Fig. 2F, H. Absorbance patterns of brain and blood EVPs. **G**, **I** DLS scattering pattern of brain and blood EVPs. **J**, **L** We extracted and quantified the total RNA concentration enriched among different EVPs from brain and blood, respectively. **K**, **M** Electrophoretic mobility (zeta potential) analysis of brain and blood EVPs. **N** Reproducible separation of large EVs, small EVs, and small EPs in 10 human subjects (5 AD and 5 controls). The dots are colored by each individual subject. Significant *P*-value (3.2e^−05^, 1.2e^−07^, 2.2e^−08^) is observed for all EVPs (*n* = 30). *P*-values were calculated using a two-sided Welch’s *t*-test, which compares group means while accounting for unequal variances. **O** EVPs isolated from AD and controls had no significant differences in their diameters (nm). The dots are colored by AD and controls (from 10 human brains, EVP *n* = 30) and blood (from 10 human serum, EVP *n* = 30). Because the data did not meet assumptions of normality, comparisons between AD and control groups were performed using a two-sided non-parametric Wilcoxon rank-sum test. **P** Plot of standard deviation versus mean log2 counts per million (CPM) for RNA-seq data from SECrifuge and UC EV isolates. **Q** PCA from RNA-seq data from UC and SECrifuge isolates from human serum. Data are presented as mean ± s.e.m. For **N**, **O** the boxplots are defined as Center line or median, the bounds of the box correspond to 25th percentile (Q1) or lower hinges and 75th percentile (Q3) upper hinges). The Whiskers extend to the smallest value no further than 1.5 × IQR from the lower hinge and the largest value no further than 1.5 × IQR from the upper hinge representing the IQR or inter-quartile range (Q3–Q1). The dots outside the whiskers are considered outliers. For **B**, **C**, **F**, **H**, **G**, **I** the experiment was repeated independently by 3 different technicians with brain tissue supernatants (from 10 human brains, EVP *n* = 30) and blood (from 10 human serum, EVP *n* = 30) with similar results. Data are presented as mean ± s.e.m. Overall, based on these data, we classified EVPs into three subpopulations—large EVs, small EVs, and small EPs (SECmeres). Collectively, we term this canopy of particles as “EVPs”.

While EVPs are heterogeneous entities with unique biophysical and biochemical properties, a broad consensus classifies EVPs based on their formation mechanism, size, and protein cargo^21^. The current gold standard EV isolation technology, ultracentrifugation (UC) lacks reproducibility^30,38^, whereas the next most popular approach, size exclusion chromatography (SEC), leads to >10–20× dilution^39^, making it unsuitable for low volume (1–2 ml) biological samples^29,40,41^. Here, to isolate EVP subpopulations from human brain and blood, we developed “*SECrifuge*”, a methodology that integrates benchtop centrifugation, SEC, and molecular weight cut off (MWCO) filtration (“Methods”, Fig. 1A). Our objective was to develop a simple, cost/time-effective, benchtop approach for reproducible isolation of EVs from human biospecimens. Contrary to the ~4–16 h needed for EV isolation with other technologies, SECrifuge can be performed in ~60 min with >10-fold enrichment (*P* < 0.05) than SEC (Supplementary Fig. 1A, B). To assess reproducibility, we isolated EVs from 10 human subject’s blood, and quantified, sequenced, and compared, their transcriptomes. Compared to UC, SECrifuge replicates were highly correlated (Rho = 0.97 vs 0.6, *P* < 0.05) (Supplementary Fig. 1C, D). When compared with other approaches, *SECrifuge* is rapid (<60 min), highly reproducible (*R*^2^ > 96%), cost-effective (<$10/prep), requires no specialist equipment, and is amenable to clinical settings (Supplementary Fig. 1E). To further ensure the rigor and reproducibility of SECrifuge in comparison to UC, we tested our methodology on a new set of 10 human subjects (5 AD, 5 controls). Dynamic light scattering (DLS) measurements displayed consistent particle size distributions and low variability across replicates, indicating high reproducibility of the isolation method (Fig. 1N). Additionally, EVPs from AD and control subjects display no significant difference in size (Fig. 1O). To measure variability in EVP cargo, we compared EVs isolated by UC and by SECrifuge. SECrifuge replicates displayed relatively low variability, while UC replicates introduced considerable additional variability, indicating highly expressed genes detected at significantly different expression levels (Fig. 1P, Supplementary Fig. 1F). This variability is likely due to isolation of different EVPs together with UC, hence the additional genetic sources, including larger vesicles and small EPs during UC processing. These data imply that SECrifuge-isolated EVs yielded more reproducible sequencing data than UC-isolated EVs. Principal components analysis (PCA) for RNA-seq data from SECrifuge EV isolates displayed lower variance compared to UC (Fig. 1Q). Finally, we compared a list of brain-derived RNA markers in each individual serum EV sample (Supplementary Fig. 1F). SECrifuge demonstrated reproducible identification of brain-derived markers in all six samples, while UC isolated samples displayed low reproducibility (Supplementary Fig. 1F). Using this approach, we fractionated and collected EVP subpopulations from human brain tissue and blood and subjected them to phenotypic and molecular characterization.

### Brain and blood-derived EVPs exhibit distinct biophysical properties

To accurately characterize the EVPs isolated in our study, we followed the minimal information for studies of extracellular vesicles (MISEV) guidelines, a field-consensus rigor initiative of the International Society for Extracellular Vesicles (ISEV)^21^. First, we conducted a comprehensive quality control analysis (see below) of EVPs to characterize their size, shape, morphology, scattering patterns, surface charges, proteins, RNAs, and canonical markers. Based on these distinct features, we classified EVPs into three subpopulations — large EVs, small EVs, and small EPs or “SECmeres” (Fig. 1B, C).

First, we measured the hydrodynamic size (using DLS) of EVPs. The sizes spanned approximately 10–1000 nm in both brain- and blood-derived EVP samples (Fig. 1B, C). We found that brain EVPs displayed increased relative absorbance at 280 nm in the small EV region (shaded red in Fig. 1B, C). In contrast, blood EVPs showed the highest relative absorbance in the small EP region (shaded Green in Fig. 1B, C). The relatively higher absorbance in blood EPs is likely due to an abundance of lipoproteins (≈10^16^ lipoproteins/mL) in blood, which are generally less pronounced in tissue microenvironment^42^. We then observed EVPs using a high-resolution transmission electron microscopy (TEM). Large EVs (~500–1000 nm) displayed large “bleb” like structures, characterized by a bulge or protrusion (Fig. 1D, E)^43^. Small EVs mostly displayed a frequently reported round cup-shaped morphology associated with ~100–150 nm particles^4^, while SECmeres mostly displayed sub-50 nm electron dense morphologies (Fig. 1D, E, unprocessed TEMs are provided in Supplementary Fig. 2A–F). Moreover, brain and blood EVPs displayed unique absorbance patterns between 250–300 nm (Fig. 1F, H). Small EVs and SECmeres exhibited high absorbance, indicating likely enrichment of biomolecules such as proteins and nucleic acids (Fig. 1F, H). In contrast, large EVs showed relatively low absorbance (Fig. 1F, H). Blood SECmeres displayed an additional absorbance band of the porphyrins at the Sorét band between 370 and 420 nm, a typical absorbance in serum^44^. Finally, we noticed a multimodal distribution of the scattering pattern from different brain and blood EVPs, indicating that there are distinct subpopulations of EVPs that are contributing to the unique counts (Fig. 1G, I).

Recent evidence suggests that large EVs and small EVs encapsulate RNAs, keeping them protected from the extracellular RNase^45–47^. We asked whether the SECmeres are resistant or susceptible to RNase. To address this, we treated each EVP subpopulation (10 human subjects’ tissue and blood, EVP *n* = 60) with RNase (Supplementary Fig. 3A–D). We found that most of the small EVs and SECmeres associated RNAs were resistant to RNase treatment (Supplementary Fig. 3A–D). Notably, formation of a protein corona on the surface of EVs and EPs can also enable resistance to exogenous enzymes^48^. We then extracted and quantified the total RNA of each EVPs, and discovered that RNA content was highest in small EVs and SECmeres (Fig. 1J, L). Finally, the three EVPs displayed unique surface charges, with small EVs exhibiting the most negatively charged surfaces (Fig. 1K, M), likely due to anionic phospholipids in the membrane, or glycoproteins on their surfaces^29,49^.

Overall, based on their biophysical properties, we identified three subpopulations of EVPs and classified them as large EVs, small EVs, and SECmeres, collectively referred to as “EVPs”. The EVPs displayed unique size, morphology, absorbance patterns, scattering patterns, RNA enrichments, protein enrichments, and surface charges. Most of the RNAs associated with small EVs and SECmeres are resistant to RNase treatment, indicating their protection from extracellular RNase^46^.

### Characterization of MISEV-recommended biomolecular cargo associated with heterogeneous EVPs

**Fig. 2:**
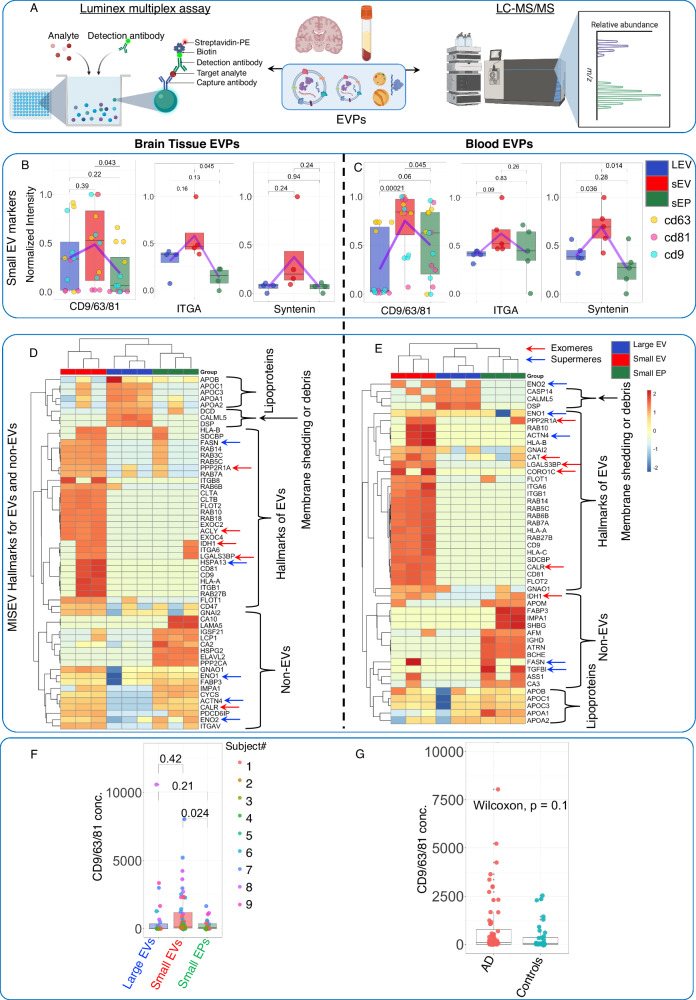
Characterization of MISEV-recommended biomolecular cargo associated with EVPs. **A** To address MISEV recommended hallmarks for EVs and NVEPs, we used two orthogonal technologies, a Luminex human exosome characterization multiplex panel and liquid chromatography coupled to high-resolution mass spectrometry (LC-MS/MS). Created in BioRender. Dogra, N. (2026) https://BioRender.com/9c4uw9y. MISEV recommended proteins for small EV hallmarks and their relative enrichment in different EVPs from brain (**B**) and blood (**C**). A curated a list of proteins from MISEV guidelines and compared with our EVP data from brain (**D**) and blood (**E**). Exomeres and supermeres associated proteins are displayed with arrows. Only significant proteins (|log2(FC)| > 0.5 and FDR < 0.05) are reported; colors represent scaled Z-score distribution, with blue for low expression and red for the high expression levels. Source Data are provided with this manuscript. **F** Relative comparison of tetraspanins in EVPs isolated from brain and blood large EVs, small EVs, and small EPs. the experiment was repeated independently with brain tissue supernatants (from 10 human brains, EVP *n* = 30) and blood (from 10 human serum, EVP *n* = 30) with similar results. Data are presented as mean ± s.e.m. **G** Relative expression of tetraspanins between AD and controls. The experiment was repeated on 10 (5AD, 5 controls, EVP *n* = 30) different subject’s EVPs from brain tissue supernatants and blood. Comparisons between AD and control groups were performed using a two-sided non-parametric Wilcoxon rank-sum test. For **B**, **C**, **F** Statistical significance between two independent groups was assessed using a two-sided Wilcoxon rank-sum test. For analyses involving multiple pairwise comparisons, *p* values were adjusted for multiple testing using the Holm method. For **B**, **C**, **F**, **G** the boxplots are defined as Center line or median, the bounds of the box correspond to 25th percentile (Q1) or lower hinges and 75th percentile (Q3) upper hinges). The Whiskers extend to the smallest value no further than 1.5 × IQR from the lower hinge and the largest value no further than 1.5 × IQR from the upper hinge representing the IQR or inter-quartile range (Q3–Q1). The dots outside the whiskers are considered outliers. Source Data are provided with this manuscript.

Next, we asked if the MISEV guideline recommended hallmark proteins for small EVs, and commonly co-isolated non-vesicular extracellular particles (NVEPs) are present in our EVP subpopulations^21^. To address this, we used two orthogonal technologies, the Luminex human exosome characterization multiplex panel and liquid chromatography coupled to high-resolution mass spectrometry (LC-MS/MS) (Fig. 2A). Here, we isolated large EVs, small EVs, and SECmeres from 10 (5 AD and 5 non-AD) different human subject’s brain and blood (total EVP samples = 60). Below, we discuss our EVPs hallmark analyses in detail:

#### Luminex assay

We conducted a Luminex assay on each EVP following the published protocols (Fig. 2A, “Methods”). The canonical tetraspanins transmembrane protein markers (CD9, CD63, CD81), which are considered the hallmarks of small EVs, significantly differentiated small EVs, and SECmeres from brain (*P* = 0.043) and blood (*P* = 0.045), respectively (Fig. 2B, C). Tetraspanin markers were also differentially expressed in blood large vs small EVs (*P* = 0.0002), small EVs vs SECmeres (*P* = 0.045), and SECmeres vs large EVs (*P* = 0.06). Other small EV hallmarks, membrane protein integrin (ITGA) and cytosolic protein (syntenin-1), were also differentially expressed in all three EVPs. In contrast, the non-EV markers, cytochrome C, Ecadhrin, and HSP90 were not significantly (*P* > 0.05) different between large EVs, small EVs, and SECmeres (Supplementary Fig. 4A–D). The debris exclusion marker HMGB, a nonhistone nuclear protein, was below the detection limits in our assay (Supplementary Fig. 4C, D). We found that while the nuclear protein HMGB1 was below the detection limits in the blood and brain-derived EVPs, Cytochrome C was elevated in brain-derived EVPs. Common adhesion proteins, Perforin and EpCAM, were not significantly enriched in EVPs (*P* > 0.05) (Supplementary Fig. 4C, D).

#### Proteomics assay

A common confounder of EV studies is that in complex biological samples such as blood serum, protein, can be associated with several particles, such as lipoproteins, Argonaute2 (AGO2), nuclear debris, and diverse EVs^50,51^. For this reason, it is important to assess the contribution of NVEPs, such as lipoproteins^52,53^. To address this concern and to uncover the ubiquitous and abundant EVP proteome, we used an unbiased and quantitative proteomic approach, namely LC-MS/MS. We curated a list of proteins from the MISEV guidelines and compared it with our EVP data. Notably, MISEV hallmarks of small EVs were enriched in small EVs of the brain and blood, including integrins (ITGA, ITGB), Flotillin (FLOT1, FLOT2), tetraspanins (CD9, CD63, CD81, CD47), heterotrimeric G-proteins (GNAO, GNAi), and RAB proteins (RAB3C, RAB5C, RAB27B, etc.) (Fig. 2D, E, F, G). RAB proteins play a crucial role in regulating membrane trafficking and vesicle transport within cells^54^. CD63 was not significantly detected in the proteomic data from EVPs. The lipoproteins (ApoA, ApoB, ApoC), which are commonly associated with HDL, LDL, vLDL, and Chylomicrons, were consistently depleted in the proteome of small EVs (Fig. 2D, E, and Supplementary Fig. 4E, F). We also investigated other common co-isolates (Argonaute proteins, nuclear proteins, calnexin, golgimatrix130, and histones), none of which were significantly (*P* < 0.05) enriched in large EVs, small EVs, and SECmeres (Supplementary Fig. 4G, H). We also investigated exomeres^25^ and supermeres^24^ associated proteins in our EVP datasets (Fig. 2D, E). We found that most of the exomeres and supermeres associated proteins were enriched in the small EV fractions. Notably, blood SECmeres in our study are smaller (10–20 nm) than the reported size of both exomeres (~35 nm) and supermeres (~25 nm), both of which were identified in in vitro *cell* cultures. In contrast, the SECmeres carried a unique set of canonical metabolic enzymes and extracellular matrix proteins (AFM, IGHD, ATRN, BCHE, CA2, CA3, CA10 FABP3, IMPA1, SHBG, LCP1). We found three proteins (CALML5, DSP, CASP14) reproducibly present only in the large EVs. These proteins associate with membrane shedding or debris and can potentially be used as markers from large EVs in blood. While tetraspanins were differentially enriched among EVPs, no significant difference in tetraspanins was observed between AD and controls (Fig. 2F, G).

Overall, to characterize MISEV-recommended proteins for small EV hallmarks and commonly co-isolated NVEPs, we have used two orthogonal technologies, the Luminex human exosome characterization multiplex Panel and LC-MS/MS. The Luminex assay significantly (*P* < 0.05) differentiated EVPs based on their exosome panel, while non-EV markers were not enriched in different EVPs. Our proteomic analyses revealed that large EVs, small EVs, and SECmeres enrich distinct hallmark proteins. As expected, small EVs enrich MISEV hallmarks proteins (integrins, Flotillins, tetraspanins, heterotrimeric G-proteins, and RAB proteins). Taken together, the EVPs carry unique biochemical cargo, tetraspanins, lipoproteins, and nuclear, mitochondrial, cytoplasmic, and extracellular cargo. These findings imply possible fundamental differences in their cargo, potentially due to different modes of their biogenesis.

### Proteomic analyses of brain and blood derived large EVs, small EVs, and SECmeres reveal that subpopulations carry distinct proteins

**Fig. 3:**
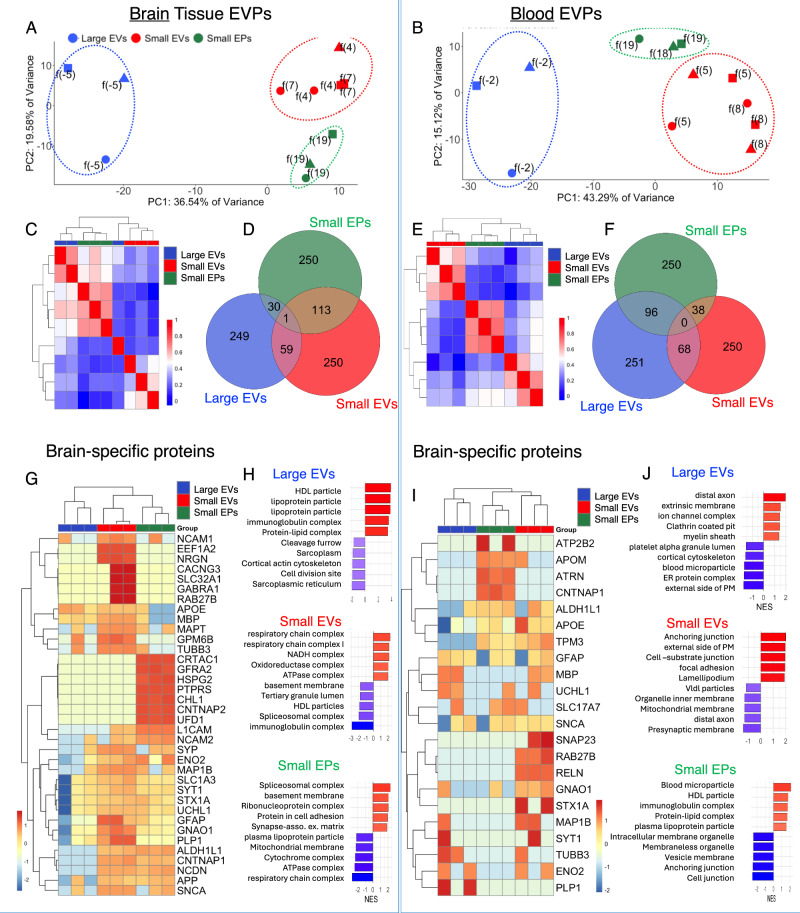
Proteomic analyses of brain and blood EVPs. **A**, **B** Principal components analysis (PCA) identified distinct clustering of EVP-proteome by their subpopulation. **C**, **E** Spearman correlation between large EVs, small EVs, and small EPs. Each EVP subpopulations are highly correlated. **D**, **F** Among the top 250 proteins enriched with EVPs, only 1 was common overlap in brain, while none in blood. **G**, **I** We curated a list of brain-specific proteins markers and compared among the EVPs. Only significant proteins (|log2(FC)| > 0.5 and FDR < 0.05) are reported; colors represent scaled Z-score distribution, with blue for low expression and red for the high expression levels. **H**, **J** Gene set enrichment analyses (GSEA) of EVPs. Source Data are provided with this manuscript.

To investigate unbiased and abundant proteins of EVPs, we used an LC-MS/MS based quantitative proteomic approach. Our proteomic analysis yielded 647 and 1470 proteins from blood and brain EVPs, respectively, that passed our filtering criteria (Methods). PCA identified distinct clustering of EVP-proteomes by subpopulation (Fig. 3A, B). The variance between EVPs indicates that the most abundant proteins in each of the subpopulations were unique. Furthermore, EVP subpopulations were highly correlated, further confirming the reproducibility of our isolation and analyses (Fig. 3C, E). Among the top 250 proteins enriched in EVPs, only 1 was common among EVPs of the brain, while none were common in blood (Fig. 3D, F). Importantly, most proteins were enriched in small EVs and SECmeres, and less abundant in large EVs. These results are consistent with our observation of low absorbance at 280 nm for large EVs (Fig. 1B, C).

Finally, we curated a list of brain-specific protein markers (Supplementary Table 1) from the Human Protein Atlas, GTEx Portal, and Allen Brain Atlas^55,56^. Over 95% of the select proteins in our curated list were enriched in the brain, reflecting brain lineage specific markers (Supplementary Table 1). We found that brain small EVs were enriched in neuronal (SNCA, GPM6B, NCAM1, NRGN, EEF1A2, CACNG3), vesicular GABA transporter (VGAT/SLC32A1), GABA-A receptor (GABRA1), and oligodendrocytes protein (MBP). SECmeres, on the other hand, were enriched in neuronal (L1CAM, NCAM2, CNTNAP1), astrocytic (ALDH1L1), and glial proteins (GFAP) (Fig. 3G). Next, we asked if brain-specific proteins are present in blood EVPs at detectable levels. We found that only a subset of the proteins detected in brain-derived EVPs were present in the blood circulation. Small EVs from blood were enriched in neuronal proteins (SNCA, SNAP23, GNAO1, ENO2, SYT1), and oligodendrocytes proteins (MBP), while SECmeres displayed astrocytic (ALDH1L1) and neuronal proteins (CNTNAP1), as well as lipoproteins (APOE) (Fig. 3I). To gain insight into the function of the EVP subsets, we conducted gene set enrichment analyses (GSEA) using the gene ontology (GO), Kyoto Encyclopedia of Genes and Genomes (KEGG) and Hallmark databases^57,58^. Among the brain EVPs, large EVs were enriched in lipoproteins and immunoglobulin complex ontologies, small EVs were enriched in mitochondrial protein complex, depleted lipoproteins and immunoglobulin complexes, and SECmeres were enriched in ribonucleic protein complexes but depleted mitochondrial ontologies (Fig. 3H). Next, we investigated blood EVPs ontologies. Blood large EVs enriched cytoskeletal proteins, small EVs enriched in membrane integral proteins, and SECmeres enriched in lipoproteins, protein-lipid associated ontologies (Fig. 3J).

Collectively, proteomic analysis of brain and blood EVPs revealed that different subpopulations carry distinct proteins. While large EVs did not significantly enrich brain markers, both small EVs and SECmeres were distinctly enriched in brain-specific proteins. While large EVs and SECmeres carried lipoproteins associated ontologies, small EVs carried integral proteins and metabolic mitochondrial protein complexes associated with protein networks. Both small EVs and SECmeres significantly carried distinct brain markers.

### The RNA content of brain and blood derived large EVs, small EVs, and SECmeres is distinct

**Fig. 4:**
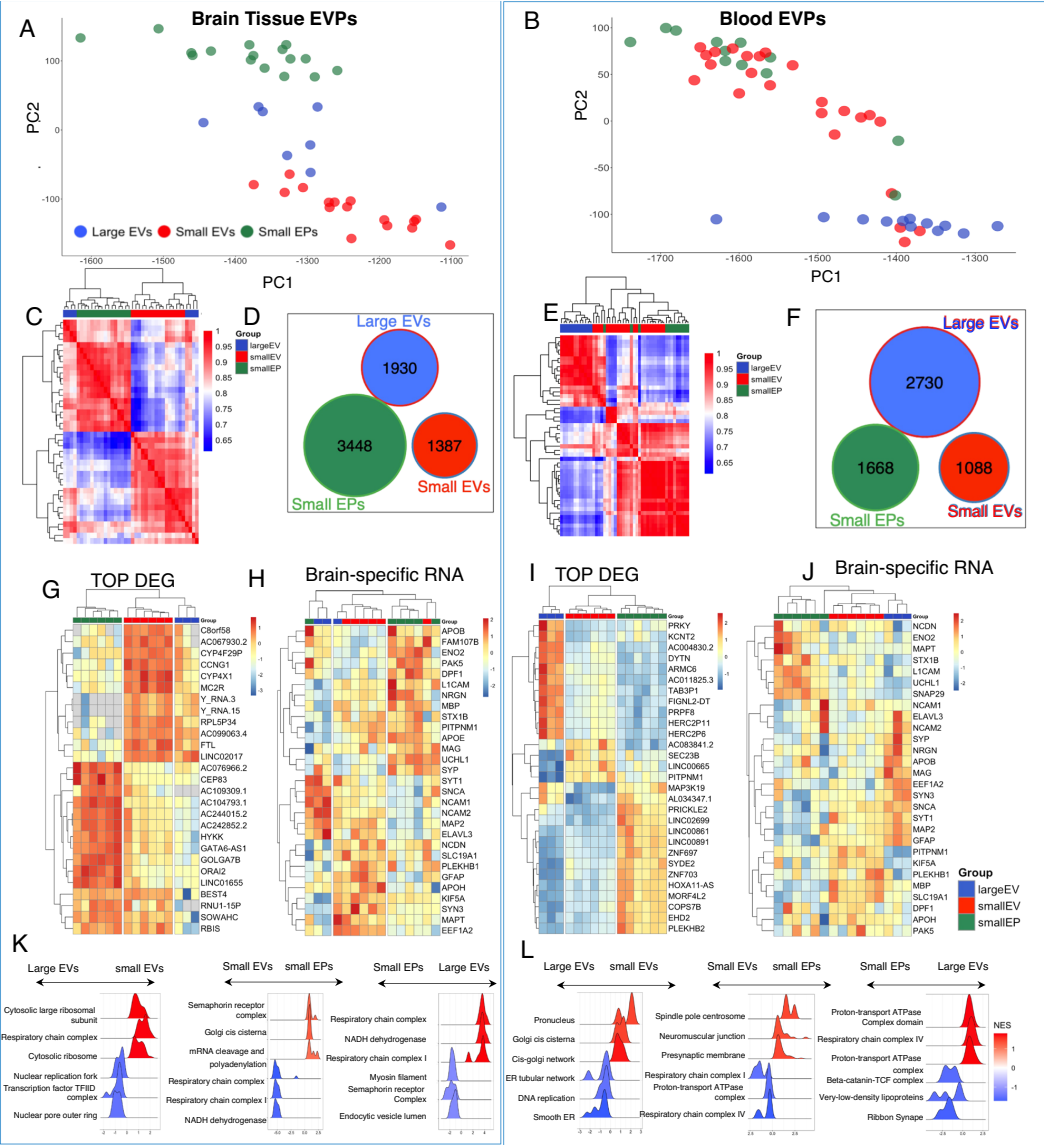
Transcriptomic cargo of brain tissue and blood-derived EVPs. **A**, **B** Principal component analysis(PCA) identified clusters of EVP-transcriptome by their subpopulation. **C**, **E** Spearman correlation between large EVs, small EVs, and small EPs. EVP subpopulations are highly correlated, further confirming reproducibility of our isolation. **D**, **F** Among the top ~6000 RNAs enriched with EVPs, no overlap was found. **G**, **I** Top differentially expressed RNAs between EVPs. **H**, **J** A curated list of brain-specific RNA markers and their relative presence in different EVPs. Only significant RNAs (|log2(FC)| > 0.5 and FDR < 0.05) are reported; colors represent scaled Z-score distribution, with blue for low expression and red for the high expression levels. **K**, **L** Gene set enrichment analyses (GSEA) of EVPs from brain and blood. Top and bottom three normalized enrichment scores (NES) are shown. Only significant RNAs (|log2(FC)| > 0.5 and FDR < 0.05) are reported. Source Data are provided with this manuscript.

Recent studies show that different EVPs carry different RNAs in cancer^22–24^. Yet, a comprehensive characterization of the diverse EVP subpopulations from the human brain and blood is overdue. Here, to assess the RNA content of EVPs, we performed total RNA sequencing (Methods) on EVPs isolated from the blood and brain of 26 subjects (*n* = 10 cases and *n* = 16 controls). In total, we identified over ∼30,000 distinct coding and non-coding RNAs, with 50% of the variance explained by PC1 and PC2 (Fig. 4A, B), indicating that the most abundant transcripts in each EVP subpopulation are unique. Using unsupervised clustering of the RNA-seq data, we were reassured that brain and blood samples displayed distinct enrichment of the RNA molecules within each EVP subtype (large EVs, small EVs, and SECmeres) (Supplementary Fig. 5A, B displays brain blood RNAs). Furthermore, EVP subpopulations were highly correlated, further confirming reproducibility of our isolation and analyses (Fig. 4C, E, G, I). Among the top ~6000 transcripts significantly enriched in EVPs, there is no overlap between large EVs, small EVs, or SECmeres, in either brain (6765 transcripts) or blood (5486 transcripts) (Fig. 4D, F). We next compared our curated list of brain-specific transcripts with the EVP transcriptomes and found that EVs from brain and blood are enriched (*P* < 0.05) in neuronal (SYT1, KIF5A) and microglial (VSIG, PROS1, PLXDC2, CX3CR1) transcripts (Fig. 4H, J) and (Supplementary Fig. 5A, B). SECmeres, on the other hand, show enrichment for a different set of neuronal (L1CAM, ENO2) and microglial transcripts (CSF1R) (Fig. 4H, J) and (Supplementary Fig. 5A, B). We then compared tissue EVPs ontologies (Fig. 4K, L), and found that large EVs were enriched in terms related to myosin filament and the nuclear complex, small EVs were enriched in mitochondrial respiratory chain complex, while being depleted in nuclear and immunoglobulin complex, and, finally, SECmeres were enriched in ribonucleic protein complexes. Next, we compared blood EVPs ontologies and observed that large EVs were enriched for membrane proteins, small EVs enriched in mitochondrial respiratory chain complex, and SECmeres enriched in lipoprotein-associated ontologies. Notably, both brain- and blood-derived small EVs enriched common mitochondrial and metabolic pathways.

Collectively, brain and blood EVPs carry distinct RNA and gene sets, which are associated with unique ontologies. Brain- and blood-derived small EVs carry common mitochondrial and metabolic pathways Importantly, blood-derived small EVs and SECmeres are enriched in brain-derived transcripts, carrying both neuronal and glial markers.

### Deconvolution of EV’s and EP’s cell of origin using brain single-nucleus RNA sequencing

**Fig. 5:**
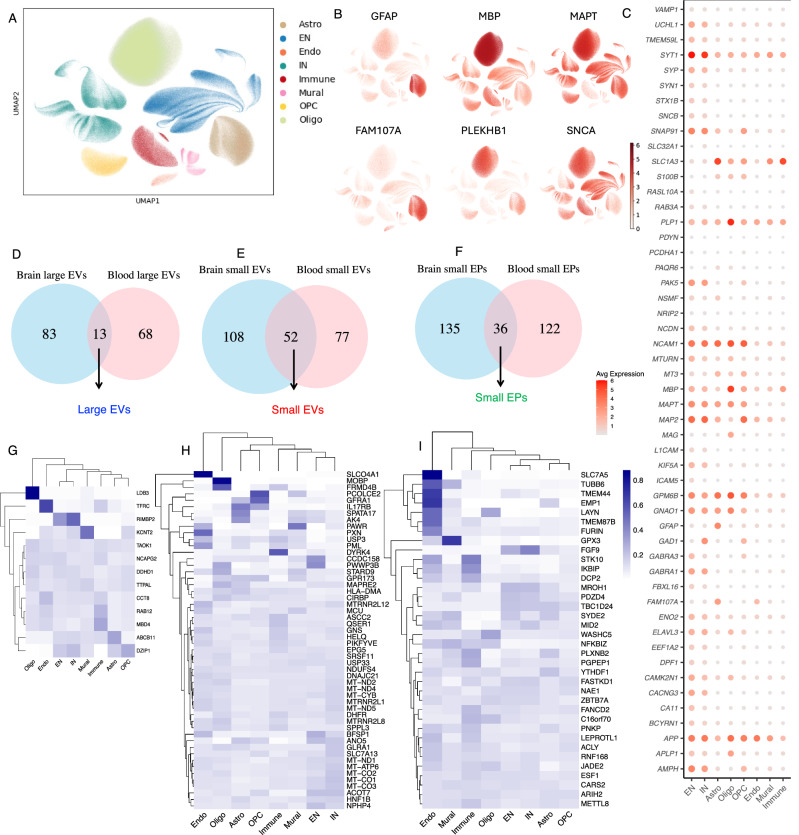
Deconvolution of EVPs’ cell of origin using single-nucleus RNA sequencing. We analyzed data from specimens (*n* = 1494) of human dorsolateral prefrontal cortex (DLPFC) generated in our lab) to infer the cell of origin for EVP-specific signatures. To quantify cell type specificity more formally, we used the “cellTypeSpecificity” function from the dreamlet R package (Methods). **A** Brain snRNA-seq revealed distinct 8 cellular clusters associated with astrocytes (Astro), excitatory neurons (EN), endothelial cells (Endo), inhibitory neurons (IN), immune cells (Immune), mural cells (Mural), other progenitor cells (OPC), and oligodendrocytes (Oligo). **B**, **C** We compared our curated list of brain-specific RNA, their enrichment in EVPs, and expression in single-cell data. **D**–**F** We compared the overlapping gene signature between brain and blood EVPs. We examined only those transcripts significantly (all genes with *P* < 0.05) detected in EVPs and evaluated their expression profiles and cell type specificity within the snRNA-seq reference. Two-sided Wilcoxon rank-sum test was used and *p* values were adjusted for multiple testing using the Holm method. **G**–**I** While large EVs and small EVs enriched in diverse brain cell-types (oligodendrocytes, astrocytes, neurons), small EPs specifically enriched in brain-endothelial cell subtypes.

Advances in bulk RNA deconvolution leveraging single-cell RNA-sequencing (snRNA-seq) have markedly expanded our ability to resolve the origins of extracellular RNA (exRNA)^59^. Emerging evidence indicates that tissue-restricted exRNA signatures within EVs can serve as robust markers of their cellular provenance^60,61^. Here, we analyzed data from specimens (*n* = 1494) of human DLPFC generated in our lab^34,35^ to infer the cell of origin for EVP-specific signatures. The snRNA-seq data revealed distinct 8 cellular clusters, namely astrocytes, excitatory neurons, endothelial cells, inhibitory neurons, immune cells, mural cells, oligodendrocyte progenitor cells, and oligodendrocytes (Fig. 5A). To derive their cell-type of origin, we then assessed the presence of our curated set of brain-specific RNAs within each cluster (Fig. 5B, C). Next, we compared the overlapping gene signatures between brain and blood EVPs (Fig. 5D–F) and their cell-type specificity (Fig. 5G–I). While SECmeres distinctly exhibited endothelial cell signatures, small EVs showed significantly greater enrichment for non-neuronal cells, whereas large EVs displayed heterogeneous enrichment patterns. Large EVs yielded a small overlap (13 RNAs) compared to small EVs (52 RNAs) and SECmeres (36 RNAs), which hindered their deconvolution (Fig. 5D–F). This analysis revealed that while small EVs are enriched for RNA from diverse brain cell-types (oligodendrocytes, astrocytes), SECmeres specifically carry transcripts from brain-endothelial cells that line the inside of blood vessels in the brain, forming the BBB (Fig. 5G–I).

Taken together, comparing human brain snRNA-seq data with EVP-specific signatures revealed that, while large EVs and small EVs were enriched in diverse brain cell-types (vascular, glial, and neuronal cells), SECmeres were specifically enriched in markers of brain-vascular endothelial cell subtypes.

### Small EVs and SECmeres from the brain and blood discriminate neuropathologically confirmed AD from controls

The definitive diagnosis of AD requires post-mortem confirmation of characteristic neuropathology, as clinical diagnoses in living individuals can be prone to misclassification due to overlapping dementia symptoms^1,2^. Our approach emphasizes that AD liquid-biopsy biomarkers may be a more trustworthy diagnostic tool for use in living patients, longitudinal studies, and clinical trials, allowing for a quantifiable molecular signature for disease progression or treatment response^3^. Here, we conducted a proof-of-concept study and asked whether the transcriptome of peripheral blood EVP’s carries disease-specific information.

**Fig. 6:**
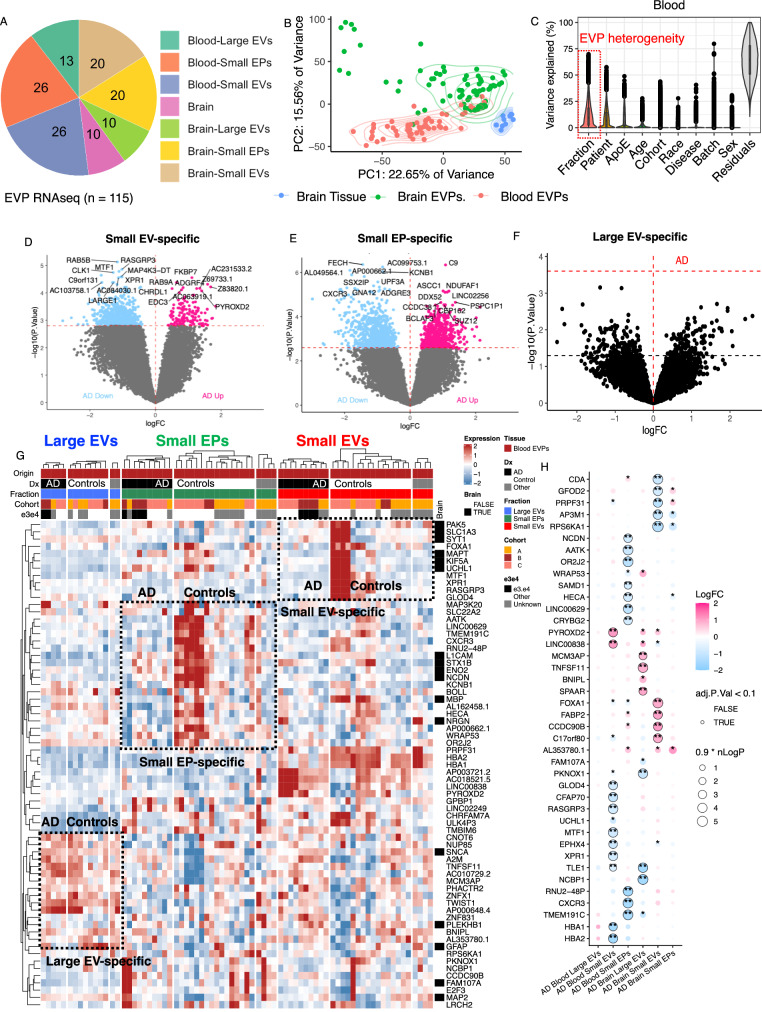
Small EVs and SECmeres can distinguish AD from non-AD subjects. **A** A diverse set of EVPs were isolated and compared. (EVP *n* = 115 samples from brain and blood of 26 (10 AD and 16 controls) subjects). **B** PCA of the transcriptome of brain tissue, brain EVPs, and blood EVPs. **C** Variance partitioning analysis for variance stabilizing transformed counts analysis to determine the effect of AD confounding factors. Violin plots describing relative explained variance by the experimental variables and their interactions and gene frequency (EVP *n* = 115 samples from brain and blood of 26 (10 AD and 16 controls) subjects). Variables are sorted sequentially by highest median of explained variance to lowest. Box plots span the IQR. The line in the center of each box plot is the median value. Whiskers depict minimum and maximum values or values up to Q3 + (1.5 × IQR) or Q1 − (1.5 × IQR). **D**, **E**, **F** While small EVs and SECmeres yielded significant (*P* < 0.05, FDR < 0.05) differences, large EVs did not yield differential RNA expression between AD vs controls in our dataset. **G** Significantly different RNA in AD vs controls, many of which are known to specifically express in brain (labeled with black right column). Final diagnostics, fraction type, batch/cohort, and apoE genotype is displayed next to the heatmap. Only significant RNAs (|log2(FC)| > 0.5 and FDR < 0.05) are reported; colors represent scaled Z-score distribution, with blue for low expression and red for the high expression levels. **H** RNA species that enabled this discrimination were uniquely enriched in either small EVs or small EPs, suggesting distinct AD signatures within these subpopulations. Only significant RNAs log2FC, and FDR are reported. Colors represent scaled LogFC distribution, with blue for low expression and red for the high expression levels. Larger circle represents larger -Log10(FDR) and higher significance. Source Data are provided with this manuscript.

In this new batch, we isolated EVPs (*n* = 115) from the brain and blood of 26 (10 AD and 16 controls) human subjects (Fig. 6A, detailed information is provided in Supplementary Fig. 6A, B, and “methods”). A total of 38% variance explained by PC1 and PC2, indicating that the most abundant transcripts in brain tissue are unique, while EVPs from brain tissue and blood displayed some overlap (Fig. 6B). Next, we performed variance partition analysis to determine the effect of AD confounding factors (demographics, age, race, sex, PMI, ApoE genotype, comorbidities, batch effect, and technical variables) in our study (Fig. 6C, Supplementary Table 2). We found that EVP subtype is the main driver of variance in our data (Fig. 6C). This data indicates that most variance is due to enrichment of different RNAs in different EVP-subpopulations (Supplementary Fig. 6C). While small EVs and SECmeres yielded significant (FDR < 0.05) differences, large EVs did not display differential RNA expression between AD and controls (Fig. 6D–F, Supplementary Fig. 6D). We identified ~60 RNAs that were significantly different between AD cases vs controls, many of which are known to be exclusively expressed in the brain (Fig. 6G). Blood-derived small EVs revealed differential enrichment in brain-specific RNAs (PAK5, SLC1A3, SYT1, MAPT, KIF5A, UCHL1) between AD vs controls (Fig. 6G, H). Similarly, blood-derived SECmeres differentially expressed brain-specific RNAs (L1CAM, STX1B, ENO2, NCDN, MBP, NRGN) between AD vs controls (Fig. 6G, H). While RNAs associated with SECmeres (21 RNAs) and small EVs (11 RNAs) discriminated AD from controls (FDR < 0.05), SECmeres had better statistical confidence and significance (FDR < 0.01), whereas large EVs showed no significant discrimination (FDR > 0.05) (Fig. 6H). The RNA species that enabled this discrimination were uniquely enriched in either small EVs or SECmeres, suggesting distinct AD signatures within these subpopulations (Fig. 6H). Overall, small EVs and SECmeres from brain and blood were able to distinguish neuropathologically confirmed AD from controls (Supplementary Fig. 7). Finally, we compared our AD EV- and EP-associated RNAs with the Braak staging system and CERAD (Consortium to Establish a Registry for Alzheimer’s Disease)^62,63^. Next, we aligned and harmonized the scale of clinical variables to facilitate annotation of both datasets (as described by Donghoon et al.^35^) and found that 9 and 4 RNA biomarkers associated with Serum AD-EPs and -EVs, respectively, we positively correlated with measures of the severity of AD neuropathology, namely diagnostic certainty of AD, CERAD, and Braak stage (Supplementary Fig. 8).

To ensure that our findings provide an unbiased outcome that is not dependent upon a small number, EVP isolation methods, and is reproducible across sites/institutions, and protocols applied for RNA-seq, we have increased our QC reproducibility and RNA-seq reproducibility in our own datasets and curated external datasets from Johns Hopkins University^36,37^ (details provided in Supplementary table 2). Our combined analyses have included new brain homogenates^36^ and iPSCs^37^ from living subjects (total samples 62, with 40 AD and 22 controls) presenting a set of overlapping 45 brain-derived RNA molecules which were represented across experiments, batches, and institutions (Supplementary Fig. 9).

**Fig. 7:**
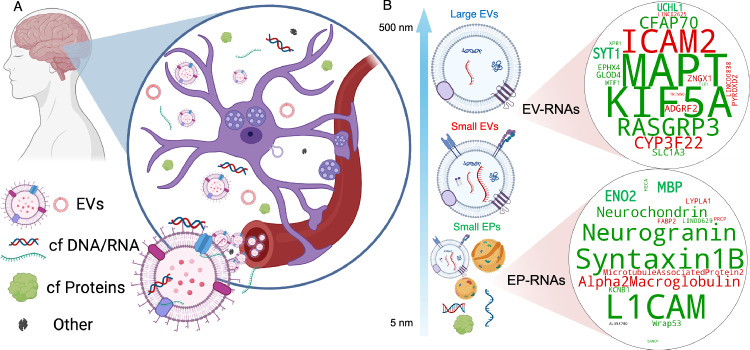
An overview of the study and findings. Created in BioRender. Dogra, N. (2026) https://BioRender.com/9c4uw9y. **A** Several brain-derived EVPs cross the blood brain barrier and reach circulation. **B** Different EVPs enrich different RNA cargo. The EVP-RNA is impacted, upregulated (green) or downregulated (red) in AD.

Taken together, RNAs associated with small EVs and EPs discriminated AD patients from cognitively normal controls, whereas large EVs showed no significant discrimination. The RNA species that enabled this discrimination were exclusively enriched in either small EVs (Synaptotagmin, Alpha-synuclein, MAPT, UCHL1) or SECmeres (L1CAM, syntaxin, ENO2, MBP), suggesting distinct AD signatures within EVP subpopulations (Fig. 7). Finally, we compared our results with other diagnostic tests and found that Braak staging, CERAD, and final diagnosis correlated with RNA biomarkers associated with Serum AD-EPs and -EVs.

## Discussion

AD is a neurodegenerative disorder that can only be definitively diagnosed through pathological examination of post-mortem brain tissue^1,2^. Here, we addressed the EVP heterogeneity, which displayed the highest variance contribution as AD cofactor (Supplementary Fig. 10). Accounting for EVP heterogeneity as a cofactor improved discrimination between AD and controls. We evaluated the diagnostic potential of brain and blood-derived EV- and EP-RNAs as liquid biopsies for AD. RNAs associated with SECmeres discriminated AD patients from cognitively normal controls with higher significance (*P* = 0.0002, FDR < 0.05) than small EVs (*P* = 0.001, FDR < 0.05), whereas large EVs showed no significant association. The RNA species that enabled this discrimination were uniquely enriched in either small EVs or SECmeres, suggesting distinct AD signatures within these subpopulations. We rigorously isolated and characterized EVP subpopulations from brains and blood of neuropathologically confirmed AD and non-AD subjects. We discovered that large EVs (>500 nm), small EVs (~150 nm), and SECmeres (<50 nm) are distinctly enriched in brain-specific proteins and transcripts. Deconvolution of EVPs using snRNA-seq revealed that, while large EVs and small EVs are enriched in markers for diverse brain cell-types (vascular, glial, and neuronal), SECmeres are specifically enriched in brain-endothelial cell subtype markers.

### Biogenesis of EVPs

The differential molecular enrichment observed among EVP subtypes may reflect their commencement through distinct biogenesis pathways, arising from separate cellular compartments and governed by selective sorting mechanisms^54^. Our proteo-transcriptomics, accompanied by gene ontology analysis, points towards unique mechanisms for EVPs biogenesis. Given the enrichment of proteins associated with transmembrane and endosomes, small EVs are likely produced in the cytoplasm and secreted via the late endosomal RAB proteins-mediated exocytosis^64^. SECmeres enriched in ribonucleic protein complexes and lipoproteins indicate multiple (both endocytic and exocytic) modes of release. Large EVs indicated direct plasma membrane shedding and lipid raft–mediated budding, given enriched cytoskeletal proteins. Additionally, the cellular state (stress, disease), and the cell type of origin (GABAergic vs glutamatergic neurons) can influence vesicle cargo, leading to unique molecular signatures in each extracellular particle subtype^3,65,66^.

### What are SECmeres?

lipoproteins (likely HDLs), nanovesicles, or NVEPs? To address this, based on our data and published results we postulate different possibilities and make three strong cases:

#### Case 1. Lipoprotein-associated RNAs

Unlike EVs, lipoproteins are micelle-like assemblies that consist of phosphatidylcholine and sphingomyelin (zwitterionic molecules)^29,67,68^, cholesterol, fatty acids, and apolipoproteins (apoA, apoB, and others)^67,69^. miRNA has been shown to bind to the apolipoproteins (ApoA-I on HDL)^70^ or phospholipids^71^, which can protect nucleic acids from RNase^70,72^. In our study, blood SECmeres are ~10–20 nm in diameter (Fig. 1C) and are enriched with apoA1 and apoM (Fig. 2E), indicating a prevalence of HDL lipoprotein-associated RNAs as the strongest case for SECmeres.

#### Case 2. A subpopulation of EVs that are sub-50 nm nanovesicles, negative for small EV markers

It is well documented that EVs exhibit subpopulations of nanovesicles carrying different proteins^73^. Our results indicate that an EV subpopulation may not carry the canonical small EV markers^21^, yet enrich other transmembrane and cytosolic proteins and small RNAs. Given that we discovered several cytoplasmic (ASS1, CA3) and transmembrane (ATRN) proteins associated with SECmeres, a subpopulation of EVs that are sub-50 nm nanovesicles, which is negative for small EV markers is also a moderate case for SECmeres.

#### Case 3. NVEP-associated RNAs

Recent publications indicate that several sub-50 nm particles, (including lipoproteins, exomeres, and supermeres) can carry proteins and RNA^24,25,70,74,75^. We found that most of the representative exomeres and supermeres associated protein markers were depleted in the SECmeres (Fig. 2D, E). However, the SECmeres carry a unique set of canonical metabolic enzymes and extracellular matrix proteins (AFM, IGHD, ATRN, BCHE, CA2, CA3, CA10 FABP3, IMPA1, SHBG, LCP1), which are not reported to associate with exomeres or supermeres. Notably, in our study, the blood SECmeres are smaller (~10–20 nm) compared to exomeres (~35 nm) and supermeres (~25 nm) (Fig. 1C). Furthermore, we would like to emphasize that the original publications that defined exomeres^25^ and supermeres^24^, conducted their experiments in different representative in vitro cell lines and used different isolation methodologies. In contrast, our study was conducted in human brain and blood, where EVPs were isolated using a benchtop, biologically/clinically relevant SECrifuge methodology, inducing minimum perturbations to the samples. Importantly, human biofluids add additional complexity to such experiments due to an abundance of lipoproteins (≈10^16^ lipoproteins/mL), extracellular matrix proteins, and other NVEPs present in blood, which are generally less pronounced in most cell cultures^42^. While SECmeres harbor distinct AD molecules, distinguishing different subpopulations under <50 nm remains technically difficult^29,76^. Here, our goal is to systematically isolate and compare their cargo with that of small EVs and large EVs from AD brains and matched blood. This goal indirectly helped us address a current controversy regarding neuronal L1CAM and its abundance in different EVPs^77^. Several recent publications have presented contrasting results^77,78^. In our proteomics data, L1CAM protein was enriched in SECmeres of the brain (Fig. 3G, H). In our transcriptomics data, L1CAM RNA was enriched in SECmeres of both the brain and blood (Fig. 4H, J). It is important to note that L1CAM is not a brain exclusive marker (gtexportal). Questions remain as to why different studies show different L1CAM enrichment. In our view, the answer lies in how EVPs are isolated, as no current methods can isolate true “exosomes” (vesicles of endocytic origin)^28,79^.

### Potential role of large EVs, small EVs, SECmeres in intercellular communication

Based on our results and published data, we hypothesize that different EVPs may have different modes of cellular entry and may enable unique cellular functions via transferring their unique cargo (RNAs and proteins)^22,80^. New isolation approaches will help unravel EVP’s contributions in intercellular communication and accelerate biomarker discovery.

Overall, our study provides a proof-of-concept that the transcriptome of peripheral blood EVPs shows disease-specific information in AD. We propose that sub-50 nm particles, which are frequently excluded in current EV isolation protocols and underrepresented in ongoing clinical trials, warrant closer investigation. While we were hesitant of adding yet another name to the already populated EVP canopy, our comprehensive proteomic and transcriptomic analyses suggest that “SECmeres” are novel SEC isolated “-meres” that carry unique proteins (Fig. 2D, E, and Fig. 3G, I) and RNAs (Fig. 4G–J). Collectively, we provide evidence that novel brain-derived EVPs may play a key role in neurodegenerative disease pathogenesis and hold promise as a real-time, non-invasive diagnostic tool for the living human brain^20^.

## Methods

We confirm that our research complies with all relevant ethical regulations and are approved by the Icahn School of Medicine at Mount Sinai Institutional Review Board (IRB). All procedures and research protocols were approved by the IRB of Mount Sinai and Mount Sinai/JJ Peters VA Medical Center IRB. The autopsy brain specimens originated from brain donation programs at the MSBB, including samples collected from JJ Peters VA Medical Center NIH Brain and Tissue Repository. All research conformed to the principles of the Declaration of Helsinki. Participants did not receive compensation. Samples were collected according to parameters of the MSBB–Mount Sinai NIH Neurobiobank cohort. All neuropsychological, diagnostic and autopsy protocols were approved by the Mount Sinai and JJ Peters VA Medical Center IRBs, with neuropathological assessments, cognitive, and medical and neurological status determinations performed. As the samples were collected postmortem, the Program for the Protection of Human Subjects office determined administratively that the project (HS no. 13-00709 PS-Psychiatry VA) is exempt human research as defined by Department of Health & Human Services regulations (45 CFR 46.101(b)).

### Brain specimen collection

Specimens were obtained from 26 de-identified subjects (10 AD brains and 16 controls) from the Mount Sinai NIH Brain Bank and Tissue Repository. The definitive diagnosis of AD was made at autopsy by the presence of abundant neuritic plaques (NP) and neurofibrillary tangles (NFT) in the neocortex, entorhinal cortex, and hippocampus. Additionally, disease diagnoses were also made based on DSM-IV criteria and obtained through direct assessment of subjects using structured interviews and/or through psychological autopsy by extensive review of medical records and informant and caregiver interviews. Age-matched donors with an equal ratio of male and female were considered for specimen collection. A comprehensive dataset of different confounding factors (age, race, ApoE genotype, sex, temporary/final diagnosis, cause of death, lobe, diabetes, smoking, depression, hypertension, early vs late onset of AD) was available and taken into consideration.

### Handling of the fresh human brain specimens

Postmortem brain tissue was transported on ice to the Neurobiobank laboratory, where a section of Brodmann area 10 was dissected, rinsed in ice-cold sterile saline, placed in ice-cold MACS tissue storage solution and immediately refrigerated (4 °C). Fresh autopsy and biopsy tissue specimens were processed using the Adult Brain Dissociation Kit (Miltenyi Biotech Cat# 130107677), according to manufacturer’s instructions. Following demyelination (Miltenyi de myelination kit, Miltenyi Biotech, Cat# 130096733) cells were incubated 10 in antibody (CD45: BD Pharmingen, Clone HI30 and CD11b: BD Pharmingen, Clone ICRF44) at 1:500 for 1 h in the dark at 4 °C with end-over-end rotation. RNase inhibitors (Takara Bio) were used throughout the cell prep. Prior to fluorescence activated cell sorting (FACS), DAPI (Thermoscientific) was added at 1:1000 to facilitate the separation of live from dead cells. Viable (DAPI negative) CD45/CD11b positive cells were isolated by FACS using a FACSAria flow 15 cytometer (BD Biosciences). Following FACS, cell concentrations and viability were confirmed using a Countess automated cell counter (Life technologies). The supernatant from this step was removed and stored at −80 °C until EVP isolation using the SECrifuge procedure, as described below.

#### Isolation of EVs

##### Isolation and separation of EVs using Integrative SECrifuge methodology

The ~2 ml blood serum or brain tissue supernatant was briefly centrifuged at 300 × *g* (cell pellet was removed), 3000 ×* g* (dead cell pellet was removed). The EVPs were separated using SEC qEV column (qEVoriginal/70 nm, Izon) in phosphate-buffered saline. A total of 20–26 SEC fractions of 500 µL each were collected. Molecular absorbance was measured for all fractions. Size (using DLS) was measured for all fractions. The fractions were collected and concentrated using Amicon Ultra-2 10 kDa filters (Millipore Sigma). Fractions consisting of 500 µL were collected in 1.5 mL microcentrifuge tubes and labeled with the sample and fraction number. Samples were immediately placed at −80 °C for future studies.

##### EVP characterization

In order to accurately characterize the EVPs, we followed the MISEV guidelines, a field-consensus rigor initiative of the ISEV^21^. As per the guidelines, we confirmed that our isolated vesicles are positive for known EV-associated: (1) Transmembrane or GPI-anchored proteins: (tetraspanins CD9/63/81), Cytosolic proteins: Flotilin-1, Alix, and caveolins, and lipoprotein-related antigens (APOA1/2 and APOB), and negative for established small EV-negative markers (calnexin, Golgi matrix protein 130 (GM130), cytochrome C).

#### Particle size and zeta potential

Particle size measurements via DLS were conducted at RT and performed with a Particle Size Analyzer (Litesizer 500; Anton Paar). Particle and intensity numbers were recorded systemically and measurement repetitions of 5–7 cycle for each sample were analyzed with particle analysis software Kalliope. Samples were then preserved for further processing. Seconds batch DLS was performed on a Malvern Zetasizer ZSP (Worcestershire, UK). Briefly for size measurements, 1 mL of sample was added to a disposable cuvette and analyzed according to the Mark-Houwink equation. Reported is the z-average of three runs per sample. Briefly, for the zeta potential, 750 µL of sample was added to a Malvin disposable folding capillary cuvette and analyzed according to the Smoluchowski equation. tThe average of three runs per sample is reported. NOTE: Fresh PBS appears necessary in order to run DLS/Zeta, as sterile, but previously opened, PBS generated ~100 nm particles and different ZP from fresh PBS.

#### EVP characterization with transmission electron microscopy (TEM)

Frozen EV pellet was brought to room temperature. Equal volumes of EVs and 3% Glutaraldehyde were mixed and kept at room temperature for 1 h. Osmium tetraoxide was added to the EV solution and was kept at room temperature for 1 h. The final EVs solution was transferred to formvar coated TEM grid and dried slowly. The grids are observed under the electron microscope at 80 kV. TEM grids are stored in the appropriate grid storage boxes for future use. Hitachi 7000 transmission electron microscope operating at 80 kV was used for imaging.

#### RNase treatment of EVPs

Brain tissue or blood samples from each SEC fraction, previously separated and stored frozen, were thawed and aliquoted (45 µL per well) into a 96-well round-bottom plate. To each well, 5 µL of either PBS or RNase (RNase A, DNase- and protease-free; 10 mg/mL, Thermo Scientific™) was added, followed by incubation at 37 °C with 5% CO₂ for 30 min. Absorbance was then measured using a NanoDrop spectrophotometer. For experiments involving lysis buffer, thawed samples were aliquoted (45 µL per well) into four wells of a 96-well round-bottom plate. PBS (5 µL) was added to two wells, and 10% (5 µL) lysis buffer (Triton X-100, SIGMA) was added to the other two wells to yield a final concentration of 1% Triton X-100. The plate was incubated at 37 °C with 5% CO₂ for 30 min. Subsequently, 5 µL of either PBS or RNase (RNase A, DNase- and protease-free; 10 mg/mL, Thermo Scientific™) was added to each pair of wells, followed by an additional 60 min incubation under the same conditions. Absorbance of all four wells was then measured using a NanoDrop spectrophotometer.

### Luminex assay protocols

#### Size exclusion chromatography

Brain tissue supernatant samples were subjected to an initial ultracentrifugation using a 10 kDa filter per manufacturer’s (Izon, 70 nm) instructions. Human serum samples were subjected to an initial benchtop centrifugation. An IZON70 SEC column was washed with 10 mL of fresh 1X PBS. After centrifugation, 4 mL of human tissue supernatant or 2 mL of human serum sample were loaded into the SEC column. The sample was allowed to load into the column, followed by 1X PBS as eluent. Fractions consisting of 500 µL were collected in 1.5 mL microcentrifuge tubes and labeled with the sample and fraction number. Samples were immediately placed at −80 °C for future studies.

#### Absorbance assay (UV-Vis)

Absorbance was measured on a NanoDrop using 2 µL of each sample.

#### Bradford assay

Protein concentration in EV fractions was determined using a Bradford assay, per the manufacturer’s instructions. Briefly, a fresh standard curve was prepared for each run. To a clear, flat-bottom 96-well plate, 150 µL of sample was loaded in triplicate. Note that lysate was 1% tritonX, and the protease was a working concentration of chymotrypsin. Then, 150 µL of the Bradford reagent was added to each well, and the plate remained at room temperature for 10 min. The data were then collected using a SpectraMax plate reader at 595 nm. A background blank consisting of PBS was subtracted from all samples prior to analysis by the standard curve.

#### Luminex assay

Briefly, samples were pooled into Large EV, small EV, small EPs. Samples were thawed prior to experimental use and added per the manufacturer’s instructions. Data was read using a Luminex 200, with the Luminex xPONENT for LC100/LX200, version 4.3 Update 1, Windows 10 Version 1909 (6.2.9200.0). Manufacturer’s protocols were followed for the ProcartaPlex Human Exosome Characterization Panel (with customization).

##### Proteomic analyses of EVPs

Each frozen pellet was homogenized by adding a predetermined volume of lysis buffer (2% SDS/1X protease inhibitor/0.1 M Ambic). The enhanced BCA Protein Quantification assay was used to determine the total protein amount from each sample. Three technical replications were run per sample. Proteins from 20 µL of EV lysates were separated from SDS using micro S-trap columns (http://www.protifi.com/s-trap/) and digested on column by trypsin. Resulting peptides were speedvac dried for LC-MS/MS analysis. Thermo Orbitrap Fusion Tribrid Mass Spectrometer was used for MS/MS analysis. Global normalization based on total number of ms/ms spectra (PSM) acquired was applied to the MS data. Spectral counts were used for semi-quantitative analysis to compare protein abundance among different samples.

Proteome Discoverer software (version 1.4) was used to search the acquired MS/MS data against a human protein database downloaded from the UniProt website. Positive identification was set at 5% protein FDR and 1% peptide FDR. Also, at least 2 unique spectra have to be identified per protein. Scaffold Proteome Software was used for post-database search processing. ~700 (for EVs) and ~1200 (for brain supernatant) proteins passed the filtering criteria and their expression profiles among these four samples were analyzed to identify differentially expressed proteins. Qlucore Omics Explorer Statistical Software was used to perform appropriate statistical analysis.

#### RNA extraction, library preparation and next-generation sequencing

Total RNA was extracted from the serum EVs using the RNAeasy kit protocol for total RNA extraction. 100–200 µL EVPs were resuspended in equal volume of ice-cold EV resuspension buffer. 2X denaturing solution was added to the final EVs solution on ice. Equal volume of acid- phenol:chloroform solution was added to each sample. The final solution was vortexed for 60 s and centrifuged at 10,000 × *g*. The top aqueous phase was carefully isolated without disturbing the lower organic phase. The top aqueous phase was transferred to the provided filter cartridge in collection tubes. Bound RNA was washed 3 times using the included wash solution. Finally, a preheated elution solution was used to elute the RNA in 20 μL volume. RNA was stored at −80 °C. RNA quality was assessed by bioanalyzer (Agilent 2100 Bioanalyzer, RNA 6000 Pico and small RNA Kit, Agilent Technologies). cDNA libraries were prepared for small RNAs using the SMARTer smRNA-seq Kit for Illumina (Takara Bio 635030). A total of 14–16 cycles of PCR were carried out to obtain a good yield of cDNA from EVPs. Final library quality was verified with Qbit and bioanalyzer. Negative (no RNA negative control, and control RNA miR163s) and positive controls provided expected results. Additionally, universal human reference RNA (UHRR) control was analyzed. Next-generation RNA sequencing was performed using a Novaseq (Illumina), 100 base pair, single-end reads at the Mount Sinai Genomics core.

### Quantification and statistical analysis

#### Careful quantification of RNA and cDNA in library preparation

Before the cDNA library prep, total RNA is carefully quantified and same amount of total RNA concentration is used for cDNA preparation. As controls, no RNA negative control, and control RNA miR163s are used. Additionally, universal human reference RNA (UHRR) control is used. Finally, the cDNA is normalized across samples and sequenced. Next-generation RNA sequencing is performed and sample read alignment to human genome is compared with negative and positive controls (typically in our samples negative controls yield <3% genome alignment). These steps make sure that similar amounts of RNA are present in each sample, allowing to compare the quantity of RNA across sample for a specific gene or non-coding region.

#### To normalize RNA-seq count data

Raw counts are first converted to log-counts per million (logCPM) using the voom method in limma. Before this transformation, library sizes are normalized to account for differences in sequencing depth across samples, commonly using the TMM (trimmed mean of *M*-values) method implemented in edgeR. TMM scales samples so that the majority of genes have similar expression distributions across libraries. The voom function then models the mean–variance relationship of logCPM values and assigns precision weights to each observation, allowing the RNA-seq data to be analyzed with limma’s linear modeling framework. Thus, normalization in a limma RNA-seq pipeline generally involves library size normalization, transformation to logCPM, and variance modeling via voom, rather than a single internal normalization procedure.

#### Genome mapping

For quantification of gene expression, raw reads were aligned to the latest Ensembl GRCh38.p13 (GCA_000001405.28) using bowtie aligner (version 2.5.4b). FeatureCounts was then used to map the aligned reads to the GENCODE v26 primary gene annotation, including transcripts corresponding to ncRNAs such as lncRNA, miRNA as well as protein-coding RNA. To maximize recovery and minimize the noise, multimapping reads were quantified up to *m* = 10 and distributed using unique reads mapping distribution, as described in most recent best practices protocols.

#### Formal analysis

Data cleaning, filtering, and analysis were performed in R and under expressed genes or proteins with low or no counts across all samples of the same phenotype were removed (at least one of the samples have CPM > 10). Normalization via trimmed mean of *M*-values in edgeR ensures library sizes of all samples are scaled properly to minimize the influences of external factors. The limma package, originally designed for microarray data, performs linear modeling on normally distributed data. Thus, to accommodate for the non-independent mean-variance relationship of RNA-seq data, the voom function assigns a precision weight derived from the library size and normalization factor of each sample itself to convert the raw counts to log2-CPM values. The log2-transformed counts minimize the changes in variance as the count size increases. Prior to examining differential expressions, we performed unsupervised clustering of samples to evaluate the similarities and dissimilarities between samples as well as across phenotypes of interest using the prcomp package in R. The result is reflected in the PCA plots. Differentially expressed genes are discerned via the standard differential expression pipeline as illustrated in limma/edgeR packages. Results of the differentially expressed genes are represented in high-resolution heatmap as well as volcano plots made using pheatmap and ggplot2 packages.

#### Correlation analyses

Spearman Rho correlations were determined across cellular and EV genetic profiles as well as the proteomic profiles. Gene expressions were plotted in the x/y axis, where x/y axis are log2 (CPM), and all RNA types were analyzed.

#### Biotype analysis

The gene biotype was recovered from the GTF annotation file for Ensembl GRCh38 (same as for alignment). Mapping resolution was kept as CDS with intron and exon annotation levels and combined to gene level when necessary. After differential expression quantification of gene biotype proportions, numbers and expression levels were taken into account. Thus, expressing gene biotype as (1) number of molecules per biotype (after library size adjustment) and (2) levels of expression using RPKM to adjust for gene/transcript length sizes.

##### Trimming and mapping

The SMARTerTM smRNA-seq kit yields reads that are flanked on the 5’ end by a leading triad of three bases from SMARTerTM template switching activity, and on the 3’ end by the Illumina adapter and extra bases from the oligo dT (which are exactly 15 bp in length). We used Cutadapt33 to remove the first 3 nucleotides of all reads, specify the homopolymer adapter sequence AAAAAAAAAA to remove along with any sequence 3’ of it, and finally discard all reads that are smaller than 15 bp long after these filters are applied. The exact command used, as recommended by the (strand-sensitive) SMARTerTM smRNA-seq kit, is cutadapt -m 15 -u 3 -a AAAAAAAAAA input.fastq > output.fastq. Therefore, our set of initial small RNAs were at least 15 bp long and were trimmed from positions 1–3 and also from the oligo dT 3’ through to the adapter. For quantification of gene expression, trimmed reads were aligned to the latest Ensembl GRCh38.p13 (GCA_000001405.28) using bowtie aligner (version 2.5.4b). FeatureCounts was then used to map the aligned reads to the GENCODE v26 primary gene annotation, including transcripts corresponding to ncRNAs such as lncRNA, miRNA, as well as protein-coding RNA. To maximize recovery and minimize the noise, multimapping reads were quantified up to *m* = 10 and distributed using a unique reads mapping distribution, as described in the most recent best practices protocols. All analysis were performed on the R studio platform using R Version 4.1 and several packages including limma, edgeR, ggplot2, reshape, tidyverse, variancePartition, pheatmap, annotables, GenomicFeatures, GenomicRanges and doParallel.

### Profiling the cellular origin of EVPs

To assess the cellular origin of extracellular vesicle particles, we queried previously generated single-nucleus RNA sequencing (snRNA-seq) data from human brain tissue samples^35^. This dataset had been transcriptionally annotated into eight major brain cell types: excitatory neurons (EN), inhibitory neurons (IN), astrocytes (Astro), endothelial cells (Endo), immune cells, mural cells, oligodendrocyte precursor cells (OPCs), and oligodendrocytes (Oligo). We examined only those transcripts detected in EVPs and evaluated their expression profiles and cell type specificity within the snRNA-seq reference. Dimensionality reduction (UMAP) was performed to visualize the distribution of these transcripts across cell types. Dot plots were generated to summarize the log-normalized average expression and the percentage of cells expressing each transcript, providing an overview of their enrichment across cell types. To quantify cell type specificity more formally, we used the cellTypeSpecificity function from the dreamlet R package (https://diseaseneurogenomics.github.io/dreamlet/reference/cellTypeSpecificity.html) which estimates the fraction of total expression attributable to each cell type. The resulting specificity scores were visualized as a heatmap to highlight cell types contributing most strongly to EVP-detected transcript expression.

### Statistics and reproducibility

All statistical analyses were performed using R (version 4.5.0). Group comparisons were conducted using two-sided Student’s *t*-tests or one-way ANOVA with post hoc Tukey’s test, unless otherwise stated. Multiple comparisons were corrected using the Benjamini–Hochberg false discovery rate (FDR) procedure. Significance was defined as *p* < 0.05 after correction. For RNA-seq, differential expression was determined using DESeq2 with an adjusted *p* < 0.05. Sample sizes were determined based on prior studies of extracellular vesicle and nanoparticle RNA biomarkers in AD; no statistical methods were used to predetermine sample size. Independent biological replicates refer to distinct patient samples. All experiments were repeated at least three times with consistent results. Data are presented as mean ± s.e.m. unless otherwise indicated. The investigator performing data analysis was blinded to diagnostic group labels during computational analyses.

### Criteria for gene selection in Fig. 6G

We applied bootstrapping to enhances the accuracy of differential analysis and the gene expression results provided a non-parametric, empirical approach to estimate variability, standard errors, confidence intervals, and *p*-values through repeated resampling with replacement from the original data. This reduces reliance on potentially mis specified parametric assumptions, leading to more robust and well-calibrated inferences, particularly in low-replicate scenarios or heterogeneous datasets common in omics. For differentials between batches (technical groupings such as sequencing runs) and cohorts (biological groupings such as patient populations or studies), we applied a specialized bootstrapping strategy: stratified resampling within batches and hierarchical resampling to respect nested structures (samples within donors or cohorts), to separate technical noise from biological signal. This improves reproducibility, controls false discovery rates more effectively, reduces bias from unmodeled batch effects or cohort heterogeneity, and increases statistical power and stability when comparing results across technical batches or biological cohorts, often yielding more reliable differential calls than standard parametric methods alone.

### Criteria to define brain-specificity

RNA lineage is specific to diverse tissue types^81^. To address their brain specificity, we carefully curated a list of commonly studied genes in the context of brain and compared their relative bulk tissue gene expression in GTEX. Our criteria were, If the top 5 tissue types are brain-tissue subtypes then the gene is termed as brain-specific. While some genes have been investigated for brain derived EVs (such as L1CAM), but they were abundantly expressed in Colon, Esophagus, and Kidney. On the other hand, GFAP RNA was overwhelmingly present in brain tissue subtypes, hence termed as highly specific brain gene.

### Limitations

First, few prior studies have characterized brain-derived small EPs, leaving limited opportunity for direct comparison and reflecting the absence of a comprehensive map of RNA species across EVP populations. Second, our sample cohort size is modest. Nonetheless, this work serves as a proof-of-principle demonstration that EVPs harbor differentially expressed AD- and brain-related biomarkers. Our next steps include validating these findings in independent, multi-institutional cohorts of living patients, as well as in ante-mortem blood samples from individuals with mild cognitive impairment or early-stage AD to assess their predictive value. We acknowledge the limited number of racial variations in our samples and, as a consequence, our claims were restricted to Caucasians due to the limited set of samples. We acknowledge that the biopsies samples may not truly represent controls. Finally, although we compared three distinct EV isolation technologies to mitigate variability, it is well recognized that the isolation method can substantially influence downstream molecular profiles. Caution is therefore warranted when interpreting data generated using different platforms. Although the sample size is adequate for discovery, more work needs to be done regarding influence of race, gender, age, stage of AD, and the influence of uncharacterized co-morbidities variably present in the autopsy samples. While care was taken to gently dissociate fresh brain tissue, it is difficult to completely eliminate the possibility of cellular contamination arising from cellular stress, rupture, or membrane blebbing during tissue processing. Consequently, the presence of intracellular components, including intracellular vesicles, in the EVP isolation from brain tissue cannot be entirely excluded. Taken together, our findings and methodology introduce a novel class of small EPs that can be leveraged in future studies to monitor diverse disease and healthy states, and to develop non-invasive molecular tools for tracking the dynamics of both coding and non-coding RNAs.

### Reporting summary

Further information on research design is available in the Nature Portfolio Reporting Summary linked to this article.

## Supplementary information

Supplementary Information

Reporting Summary

Transparent Peer Review file

## Source data

Source Data

## Data Availability

Data reported in this paper has been shared with the manuscript by the lead contact. Transcriptomics and Proteomics data is available at 10.5281/zenodo.20073415. We confirm that 10.5281/zenodo.19631706 links the code (GitHub) and the data. Source data are provided with this paper.

## Code availability

This paper does not report original code. The code used is available on https://github.com/eegk/EVP-RNA-outperforms-extracellular-vesicles-as-blood-biomarkers-for-Alzheimer-disease. Any additional information required to reanalyze the data reported in this paper is available from the lead contact upon request.
