# Supplementary material for: SECmeres outperform extracellular vesicles as potential blood RNA biomarkers for Alzheimer’s disease
Source: Nat Commun. 2026 Jun 22;17:5453. doi: 10.1038/s41467-026-74541-8 (PMC13287576; doi:10.1038/s41467-026-74541-8)
Supplement: Supplementary file 1 — Supplementary Information [file 41467_2026_74541_MOESM1_ESM.pdf]

SECmeres Outperform Extracellular Vesicles as Potential Blood RNA Biomarkers for Alzheimer's Disease

Edgar Gonzalez-Kozlova[#,1], Swapnil Tichkule[#,2], Yohei Nose[#,1], Tzu-Yi Chen[#,3], Eduard Reznik[3,4], Juliet V Santiago[5], Anish Korrapati[3], Taliah Soleymani[3], Roman Kosoy[2,6,7], Igor Figueiredo[1], Donghoon Lee[2,6,7], Gabriel E Hoffman[2,6,7], Natasha Kyprianou[3,4], Ronald E. Gordon[3], Carlos Cordon-Cardo[3], Srikant Rangaraju[8], Nicholas T Seyfried[5], Vahram Haroutunian[10], John, F. Fullard[2,6,7], Panos Roussos[2,6,7,9,10], Navneet Dogra[3,11,12,13]**

Supplementary Information

Supplementary Figures 1-10

Supplementary Tables 1-2

Affiliations:

[1] Department of Immunology, Icahn School of Medicine at Mount Sinai, New York, NY, 10029 USA

[2] Department of Psychiatry, Icahn School of Medicine at Mount Sinai, New York, NY, 10029 USA

[3] Department of Pathology, Molecular and Cell-Based Medicine, Icahn School of Medicine at Mount Sinai, New York, NY, 10029 USA

[4]Department of Urology, Icahn School of Medicine at Mount Sinai, New York, NY, 10029 USA

[5] Department of Biochemistry and Neurology, Emory University, Atlanta, GA 30322 USA

[6] Department of Genetics and Genomics Sciences, Icahn School of Medicine at Mount Sinai, New York, NY, 10029 USA

[7] Center for Disease Neurogenomics, Icahn School of Medicine at Mount Sinai, New York, NY, 10029 USA

[8] Department of Neurology, Yale University, New Haven, CT 06510

[9] Center for Precision Medicine and Translational Therapeutics, James J. Peters VA Medical Center, Bronx, NY, 10468 USA

[10] Mental Illness Research, Education, and Clinical Center (VISN 2 South), James J. Peters VA Medical Center, Bronx, NY, 10468 USA

[11] Icahn Genomics Institute, Icahn School of Medicine at Mount Sinai, New York, NY, 10029 USA

[12]Artificial Intelligence and Human Health, Icahn School of Medicine at Mount Sinai, New York, NY, 10029 USA

[13] Alzheimer's Disease Research Center, Icahn School of Medicine at Mount Sinai, New York, NY, 10029 USA

# These authors have contributed equally to the work.

*** Correspondence**

- Dr. Navneet Dogra, PhD

  Email: navneet.dogra@mssm.edu

- Dr. Panos Roussos, MD, PhD

  Email: panagiotis.roussos@mssm.edu

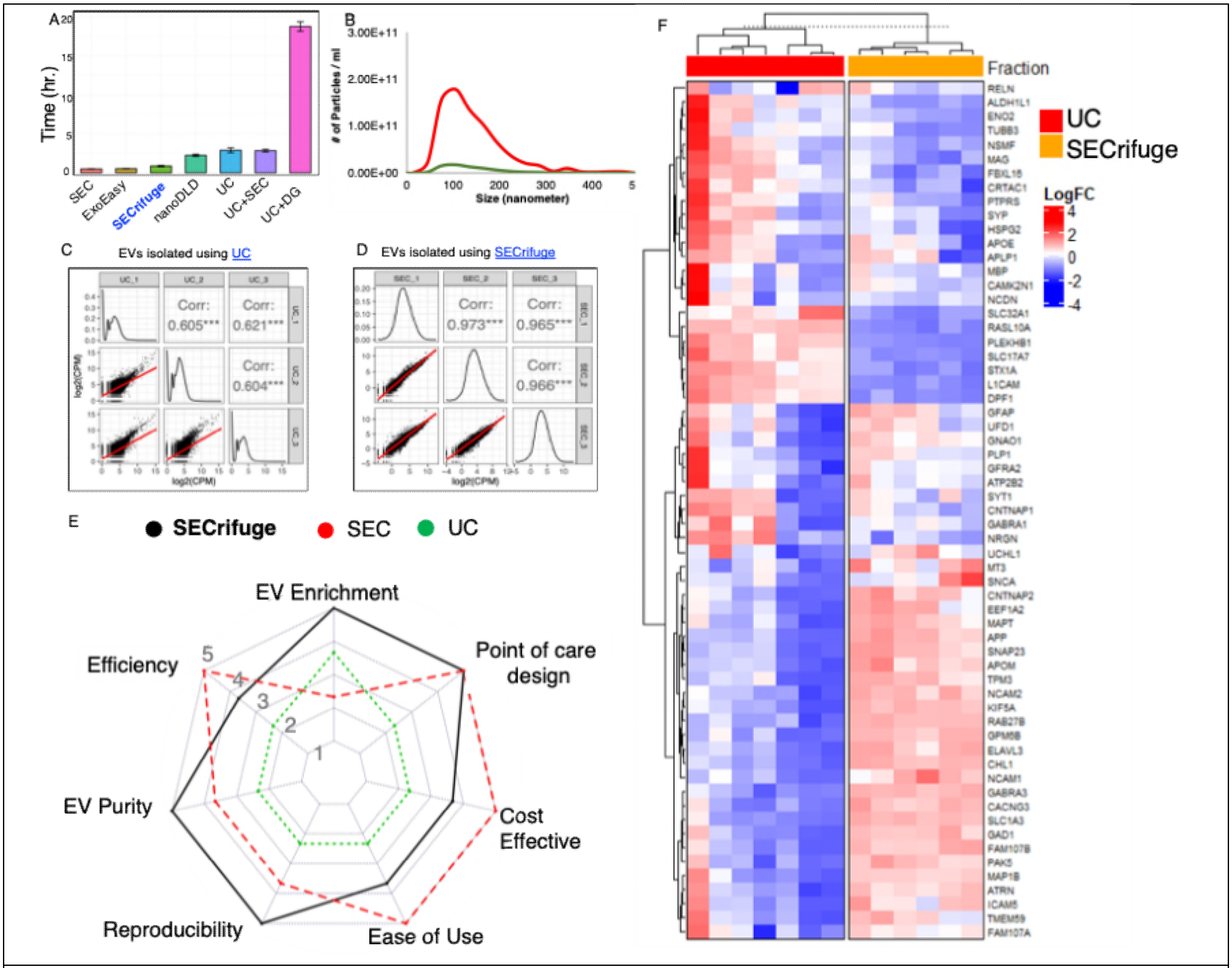

Supplementary Figure 1. Comparison of different EV isolation technologies. A. Measured run time to isolate EVs from technologies. Bars are means of biological replicates, n=3 independent experiments. Upper and lower error bars show the standard error of means from 3 independent experiments. **B.** Particle enrichment assessed by nanoparticle tracking analyses (NTA). *SECrifuge* technology isolated EVs in less than one hour with >10 fold enrichment. Reproducibility of isolation. RNA-seq of small EVs isolated using UC (**C**) and our SECrifuge method (**D**). UC yielded low (rho = 0.6, P-value <0.05), while the SECrifuge technology yielded high (Rho = 0.97, P<0.05) correlation. **E**) Performance metric factors are assessed, ranked, and plotted on a radar plot. Seven key factors ranked on a scale of 1-5 (5 being the best) for all technologies. **F**. Reproducibility of Brain-derived RNA among different human subjects. RNA-seq analysis of SECrifuge and UC-isolated EVs. Brain-derived RNAs are compared among each separate EV isolation from human serum.

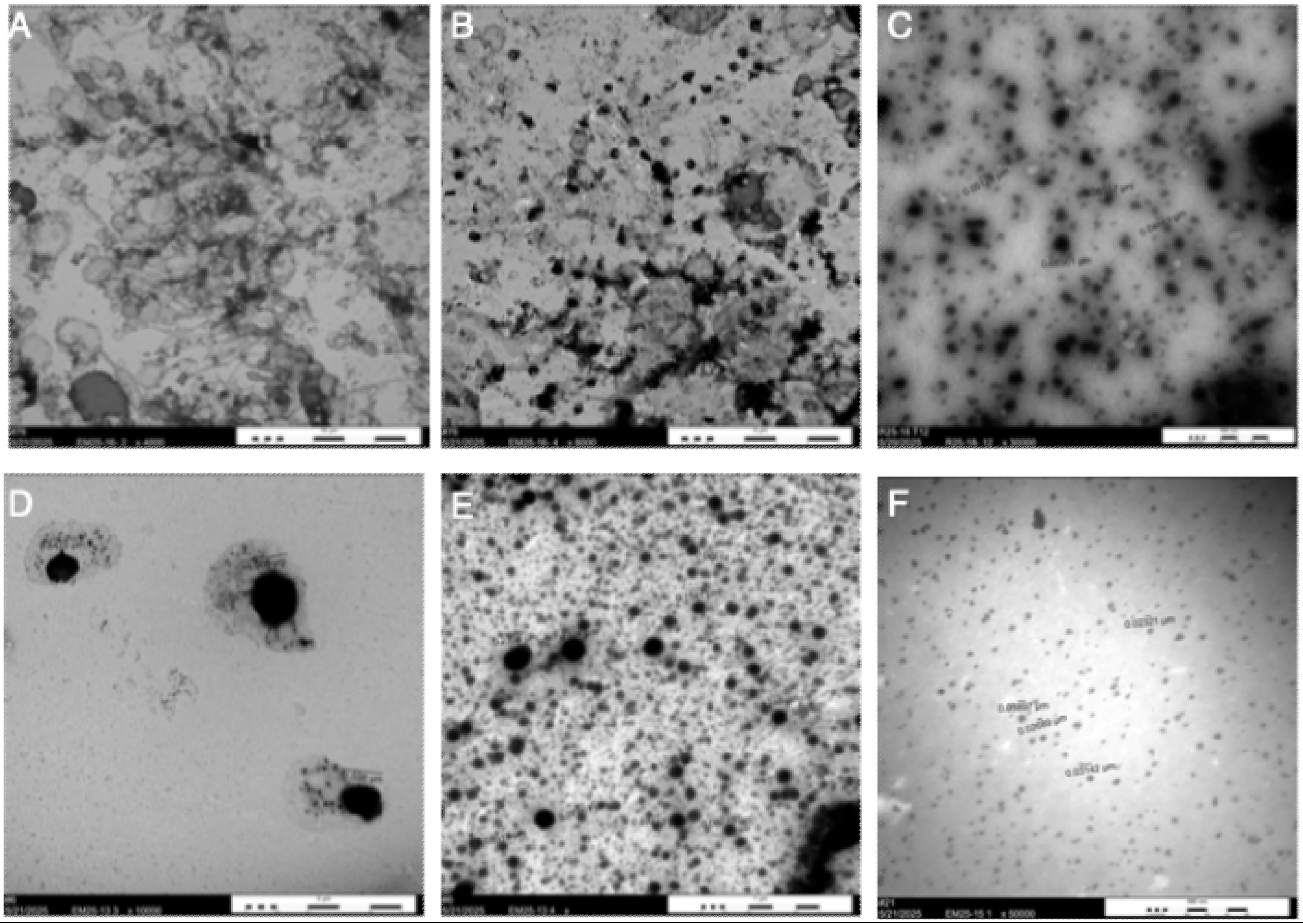

Supplementary Figure 2. Transmission electron microscopy (TEM) reveals distinct morphologies and sizes of EVPs from brain and blood. A-C. Unprocessed TEMs of brain-derived large EVs (A), small EVs (B), and small EPs (C). **D-F.** Unprocessed TEMs blood-derived large EVs (D), small EVs (E), and small EPs (F). This experiment was repeated with 3 biological replicates on 9 different EVP fractions with similar results.

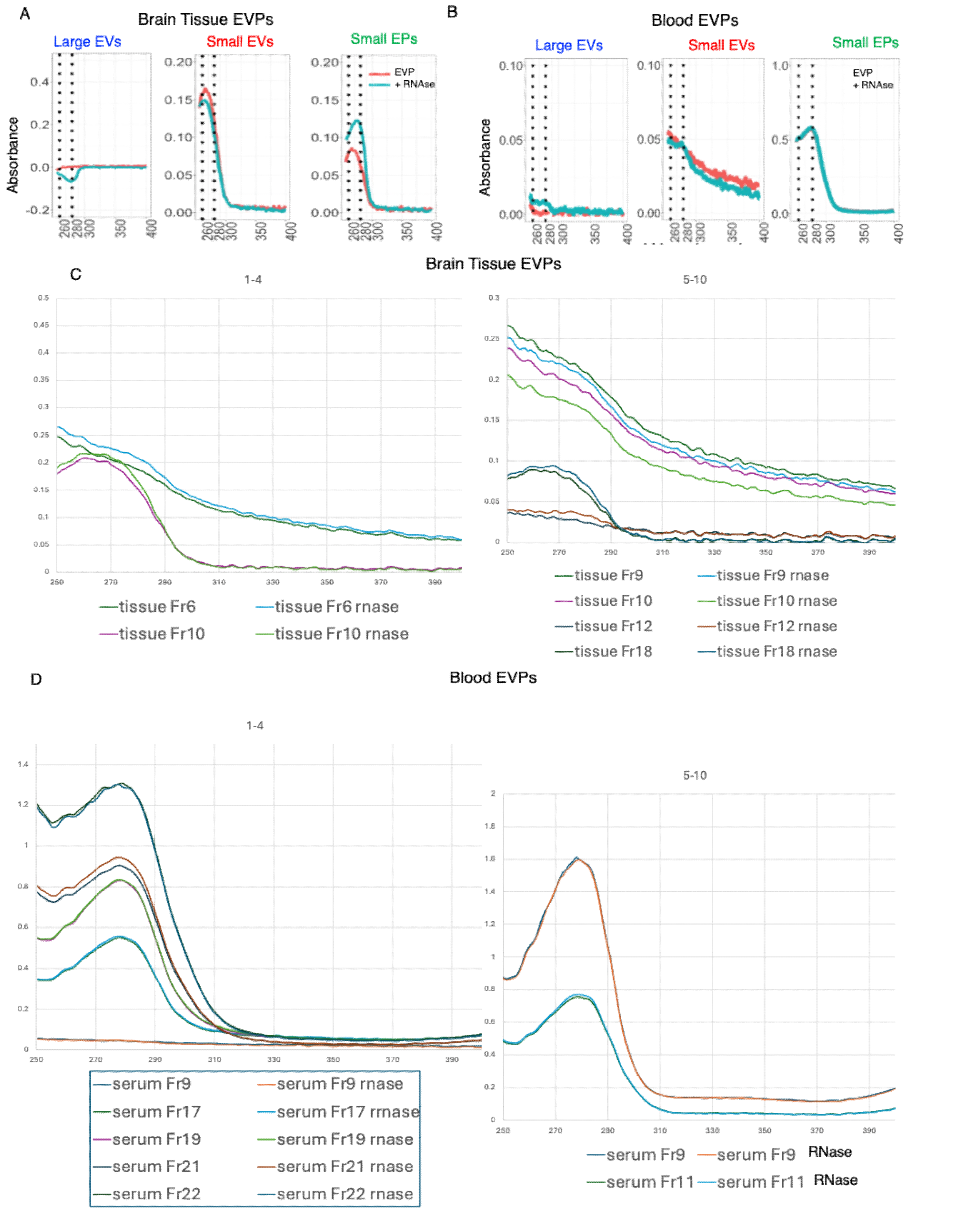

Supplementary Figure 3. EVPs are resistant or susceptible to RNase. A. Brain Tissue EVPs treatment with RNase. **B.** Blood EVPs treatment with RNase. **C**. Brain tissue EVP replicates. **D**. Blood EVP replicates. We treated EVP subpopulations with RNase and measured absorbance between 250-400 nm. Dotted vertical lines are positioned at 260 and 280 nm, respectively. biological replicates: The experiment was repeated

independently by 3 different technicians with brain tissue supernatants (from 10 human brains, EVP n = 30) and blood (from 10 human serum, EVP n = 30) with similar results.

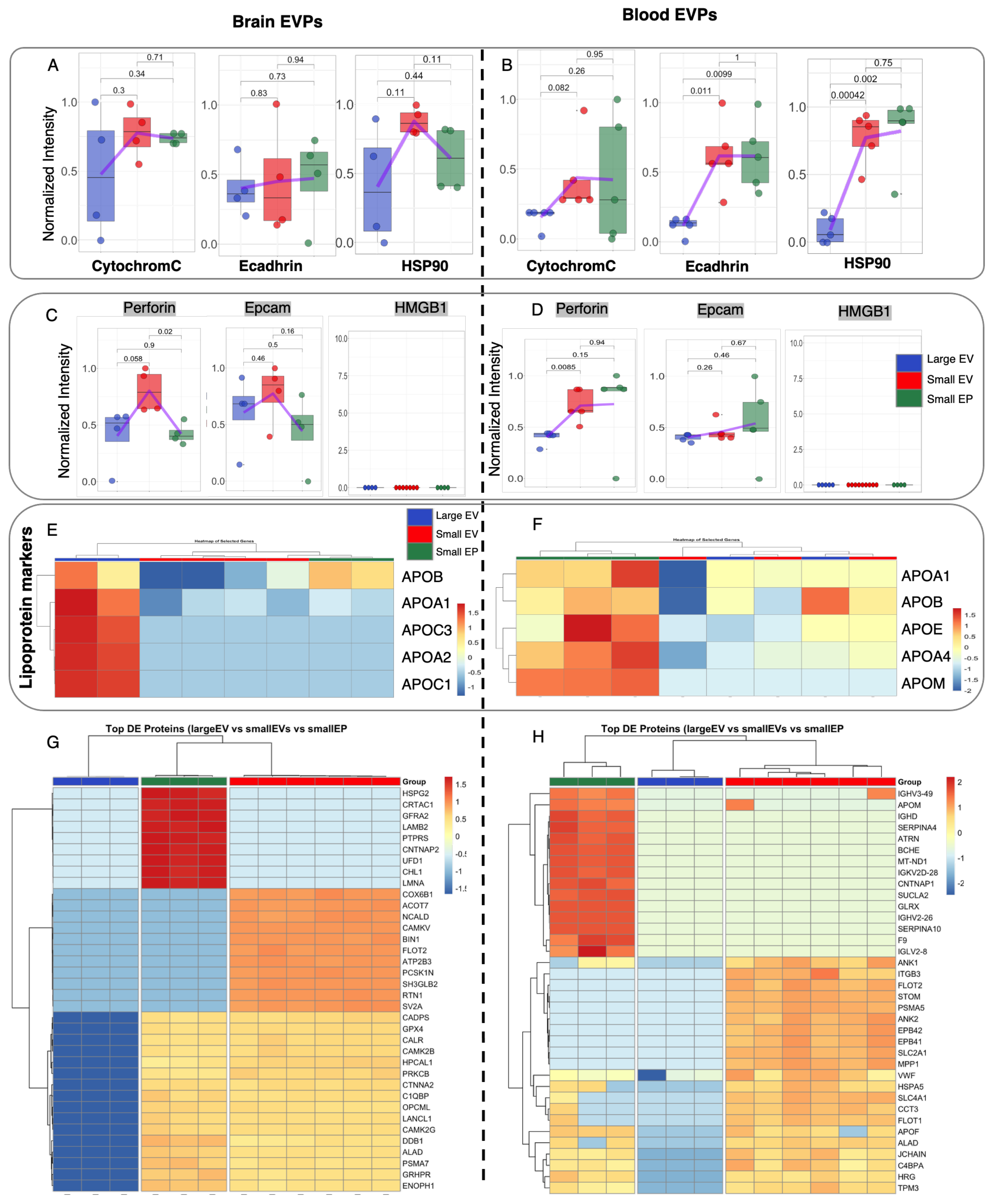

Supplementary Fig. 4. Characterization of MISEV-recommended biomolecular cargo associated with EVPs. To address MISEV recommended hallmarks for EVs and NVEPs, we used two orthogonal technologies, a Luminex human exosome characterization multiplex panel and liquid chromatography coupled to high-resolution mass spectrometry (LC-MS/MS). The experiment was repeated on biological

replicates: 3 different subjects with brain tissue supernatants (EVP n = 9) and blood (EVP n = 9). **A-B**. Luminex human exosome assay shows that non-EV specific RNAs are not significantly enriched in brain (**A, C**) and blood-derived EVPs (**B, D**). The y-axis for HMGB is concentration, to avoid biased normalized intensity for zero expression. Statistical significance between two independent groups was assessed using a two-sided Wilcoxon rank-sum test. For analyses involving multiple pairwise comparisons, p values were adjusted for multiple testing using the Holm method. the boxplots are defined as Centre line or median, the bounds of the box correspond to 25th percentile (Q1) or lower hinges and 75th percentile (Q3) upper hinges). The Whiskers extend to the smallest value no further than 1.5 × IQR from the lower hinge and the largest value no further than 1.5 × IQR from the upper hinge representing the IQR or inter-quartile range (Q3 − Q1). The dots outside the whiskers are considered outliers. **E-F**. Relative abundance of lipoprotein markers in EVPs. **G-H** Top 40 proteins enriched in brain (**G**) and blood-derived (**H**) EVPs. Source Data are provided with this manuscript.

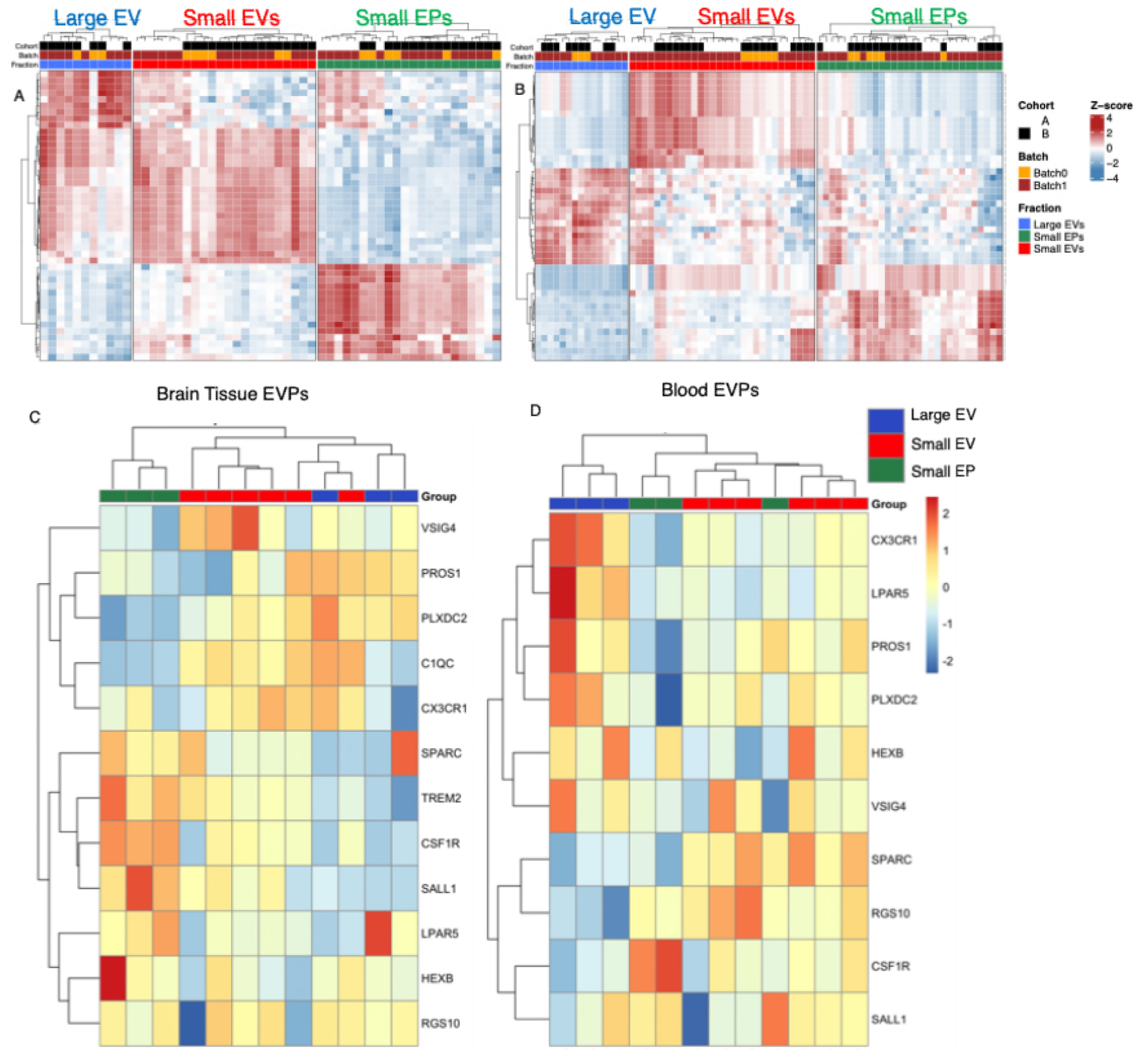

Supplementary Figure 5. Enrichment of the RNA within EVP subtype (large EVs, small EVs, and small EPs) from brain and blood. A. Brain EVPs isolated from 10 human subjects. **B** Blood EVPs isolated from 10 human subjects. A and B are two different cohorts of subjects. Batch 0 and Batch 1 are two different sequencing batched of EVPs isolated from human subjects. EVPs are labeled as fractions. Microglial RNA signature and its presence in brain (**C**) and blood (**D**) derived EVPs.

Gene	CellType	Brain Enrichment	Blood Enrichment
**UCHL1**	Neuron, neuroendocrine<br>Brain – Hypothalamus<br>associated with Parkinson disease.	Small EPs (<50nm)	Small EPs (<50nm)
**L1CAM**	Brain - Cerebellum	Small EVs<br>Small EPs	Small EPs
**ENO2**	found in mature neurons and cells of neuronal origin,<br><br>GTEX: te (basal ganglia)Brain - Cerebellar Hemisphere	Small EPs	Small EPs
**FAM107A**	Synaptic, GTEX: all brain	Small EPs	Small EVs<br>Small EPs
**PAK5**	GTEX: Brain - Cerebellar Hemisphere	Small EPs	Small EPs
**STX1B Syntaxin 1B**	Brain - Cerebellar Hemisphere	Small EVs<br>Small EPs	Small EVs<br>Small EPs
**KIF5A**	Brain – Cortex, Brain - Front	Small EVs	Small EVs
**PLEKHB1**	Brain - Spinal cord	Small EVs	Small EVs
**SYT1 Synaptotagmin-1,**	Brain - Frontal Cortex (BA9)	Small EVs<br>Large EVs	Small EVs<br>Large EVs
**GFAP glial fibrillary acidic protein**	Brain - Spinal cord (cervical c-1,<br>Glial	Small EVs	Large EVs<br>Small EVs
**MAP2 microtubule associated protein 2**	Brain	Small EVs<br>Large EVs	Large EVs<br>Small EVs
**SNCA synuclein alpha**	Brain - Cerebellar Hemisphere	Large EVs<br>Small EVs	Large EVs<br>Small EVs
**SLC1A3**	Glia		
**NCDN Neurochondrin**	Brain - Nucleus accumbens (basal ganglia)	Small EVs<br>Small EPs	Small EVs<br>Small EPs
**MBP myelin basic protein**	Brain - Spinal cord (cervical c-1)	Small EVs<br>Small EPs	Small EVs<br>Small EPs
**MAPT microtubule associated protein tau**	Brain - Cerebellum	Small EVs<br>Small EPs	Small EPs
**NRGN neurogranin**	Brain - Frontal Cortex (BA9)	Small EVs<br>Small EPs	Large EVs<br>Small EPs

Supplementary Table 1. Enrichment of brain-derived RNAs in different EVPs.

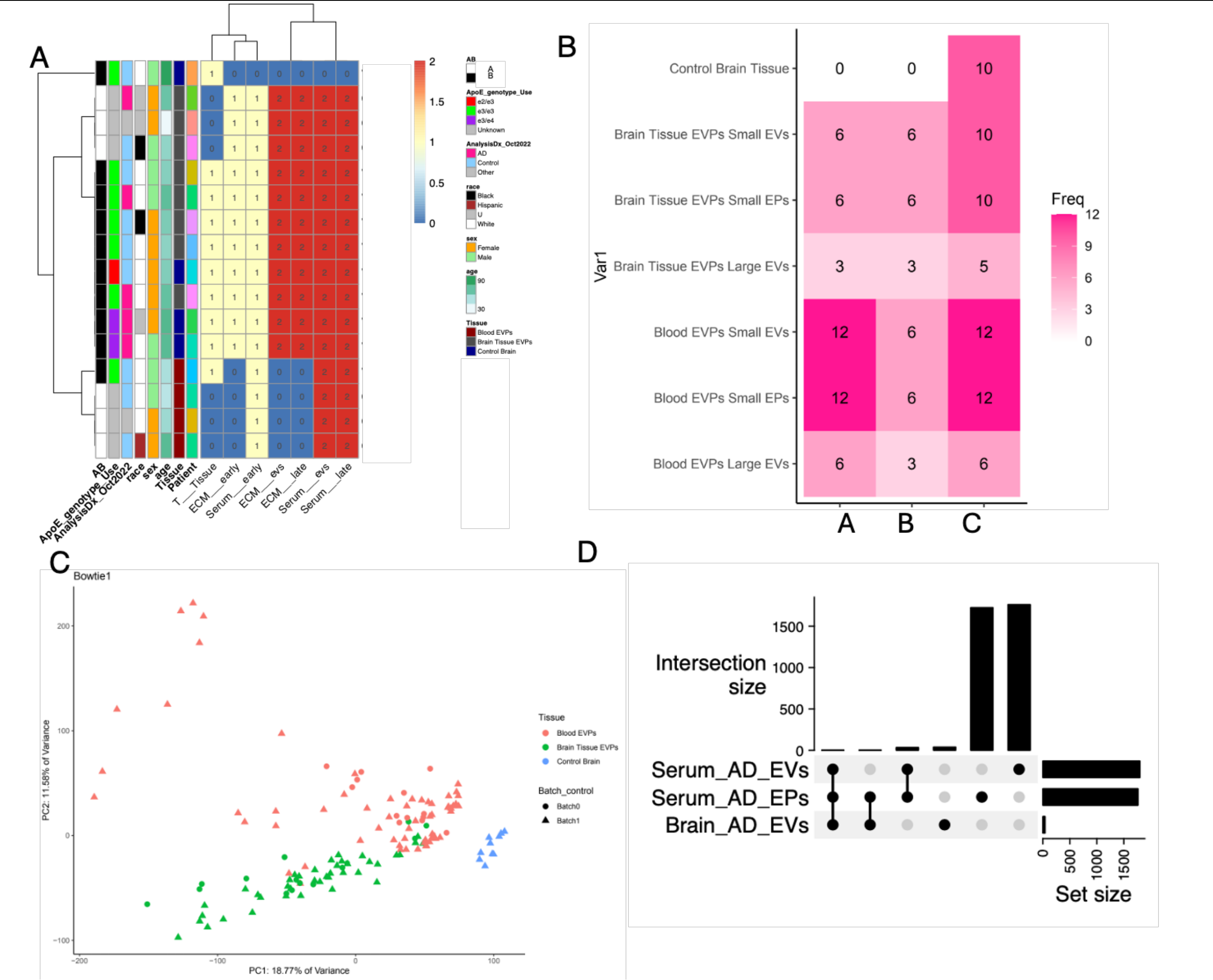

Supplementary Figure 6. Small EVs and small EPs can distinguish AD from non-AD cases. A. Different AD confounding factors and samples considered in this study. **B**. Different EVP samples used in this study. **C**. PCA of the transcriptome of brain tissue, brain EVPs, and blood EVPs. **D**. Overlap of RNAs among different EVPs. Source Data are provided with this manuscript.

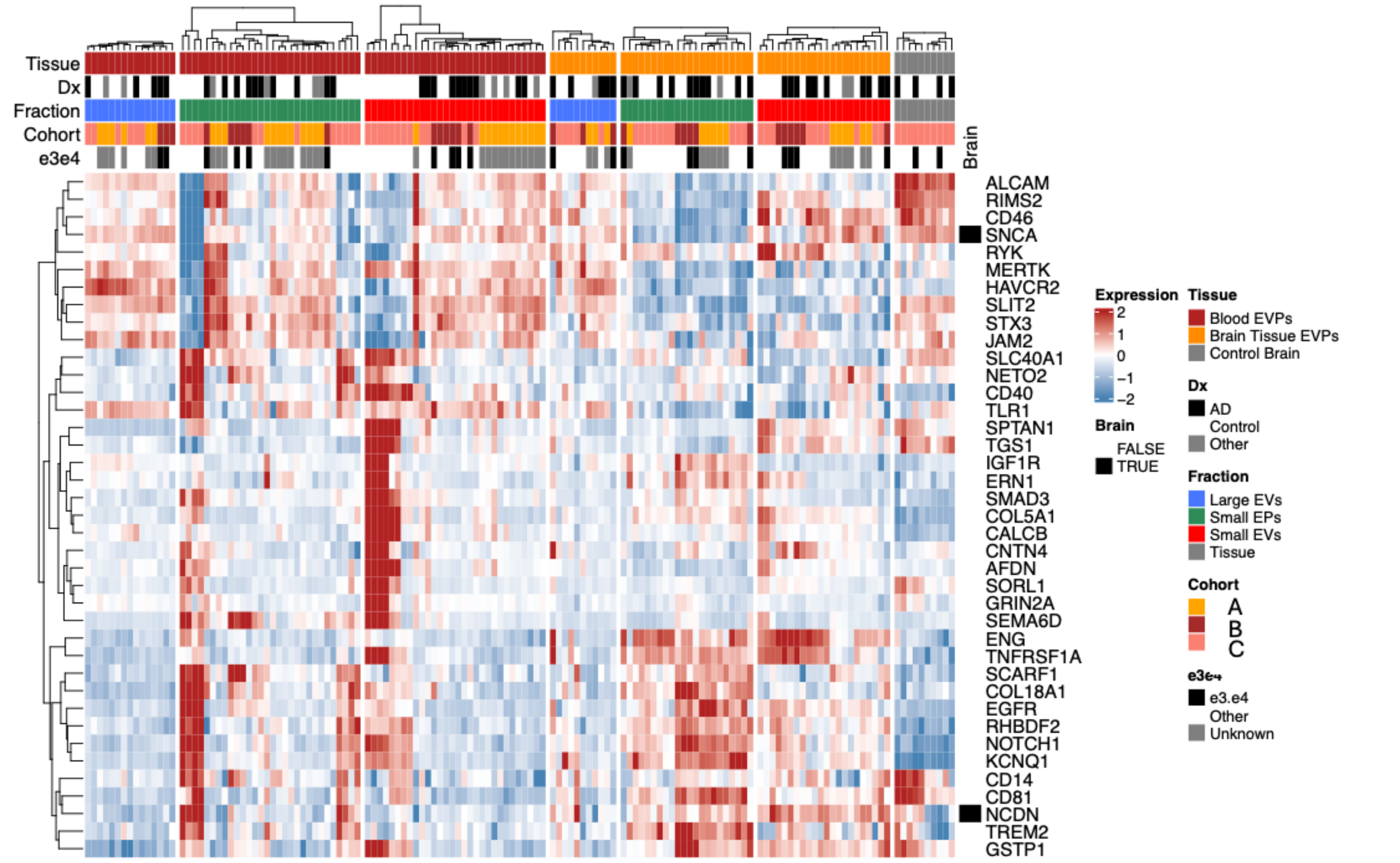

Supplementary Figure 7. Brain and blood-derived small EVs and small EPs can distinguish AD from non-AD cases. Significantly different RNA in AD vs controls, many of which are known to exclusively express in brain (labeled with black column). Only significant RNAs (|log2(FC)| >0.5 and FDR <0.05) are reported; colors represent scaled Z-score distribution, with blue for low expression and red for the high expression levels. Source Data are provided with this manuscript.

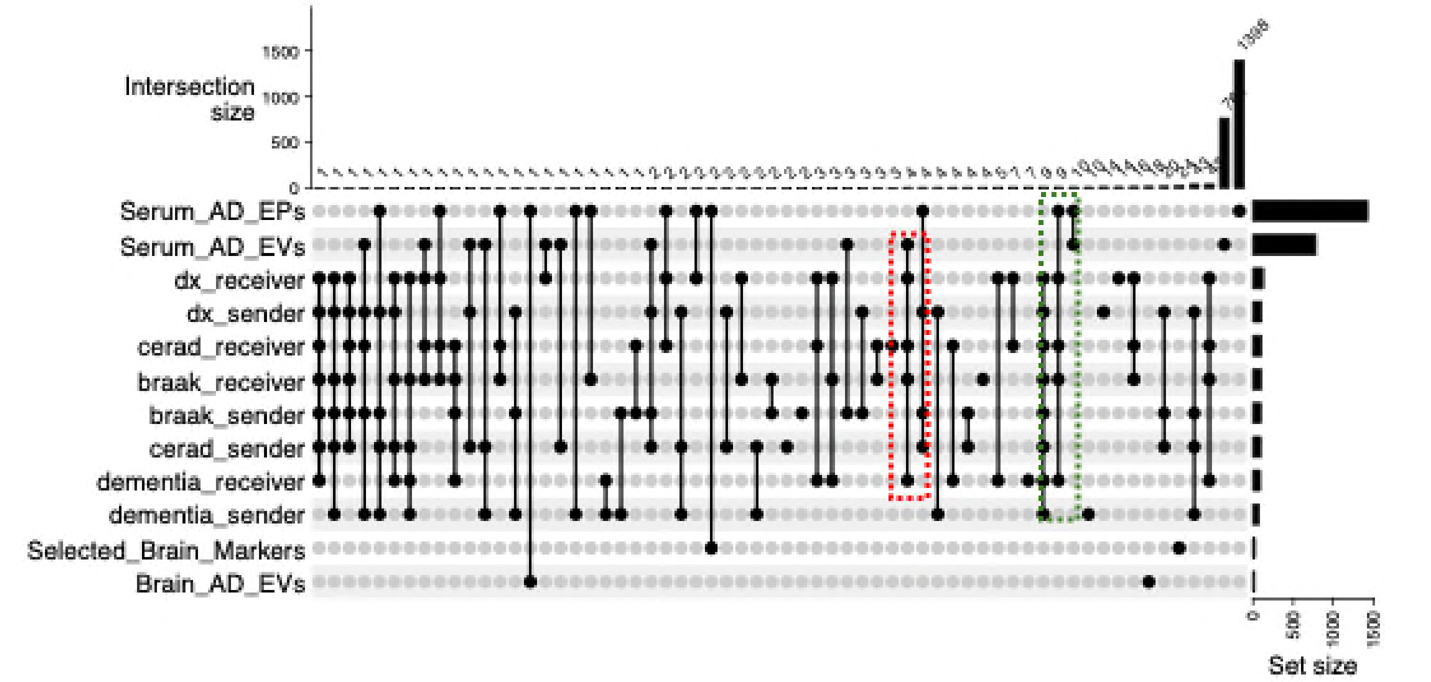

Supplementary Figure 8. Comparison of AD-EV biomarkers with current diagnosis. We compared our EV and EP-based diagnostics with Braak staging system and CERAD (Consortium to Establish a Registry for Alzheimer's Disease). 9 and 4 RNA biomarkers associated with Serum AD-EPs and -EVs, respectively, correlated with CERAD, Braak, and final diagnosis of AD cases. The small EV overlap is highlighted in red, and small EPs is in Green.

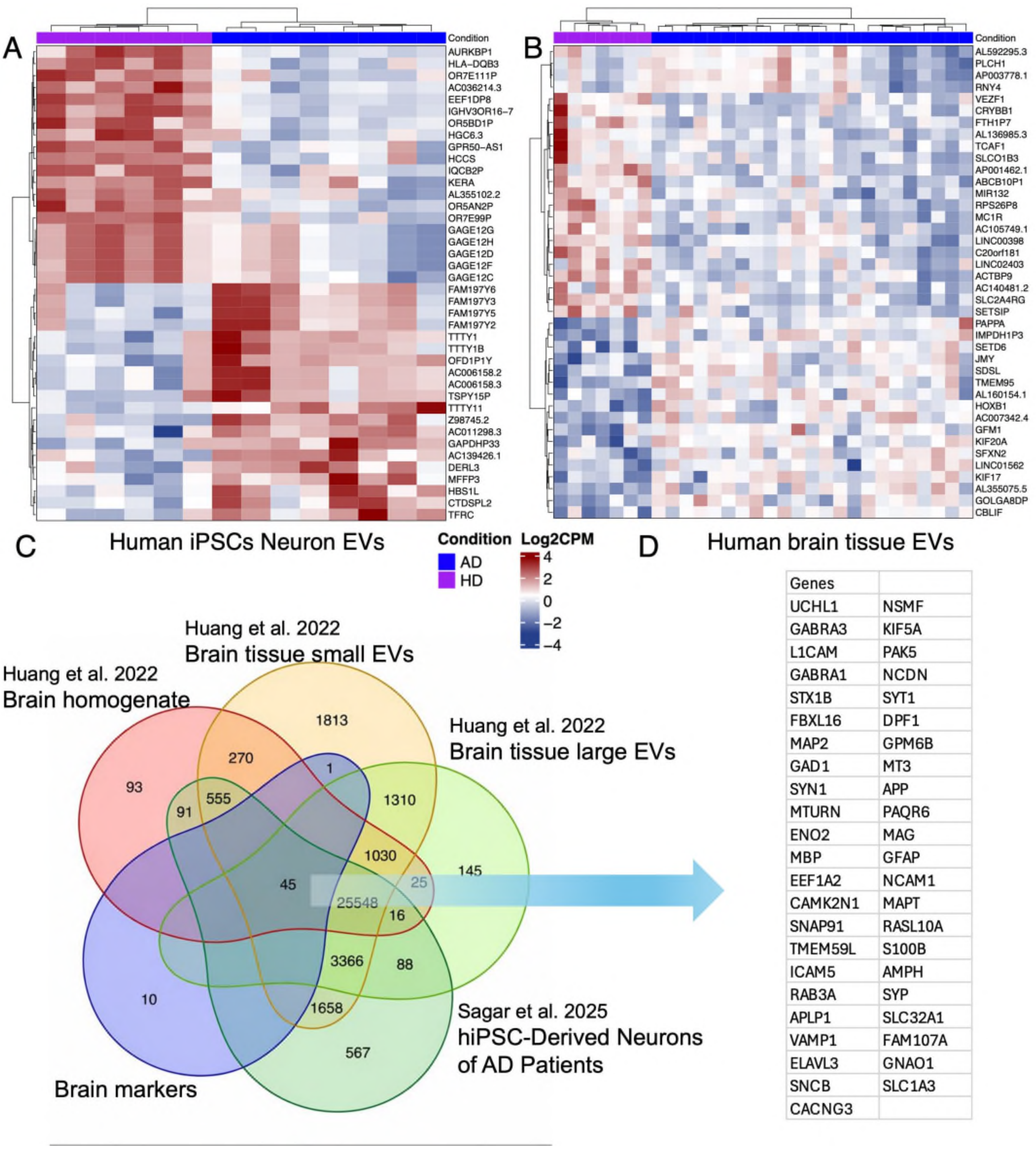

Genes	
UCHL1	NSMF
GABRA3	KIF5A
L1CAM	PAK5
GABRA1	NCDN
STX1B	SYT1
FBXL16	DPF1
MAP2	GPM6B
GAD1	MT3
SYN1	APP
MTURN	PAQR6
ENO2	MAG
MBP	GFAP
EEF1A2	NCAM1
CAMK2N1	MAPT
SNAP91	RASL10A
TMEM59L	S100B
ICAM5	AMPH
RAB3A	SYP
APLP1	SLC32A1
VAMP1	FAM107A
ELAVL3	GNAO1
SNCB	SLC1A3
CACNG3	

Supplementary Figure 9. Overlapping RNAs across studies. A. Top differential RNAs from the RNA-seq of Small EVs isolated from human iPSCs neurons (AD and healthy donors(HD)). **B**. Top differential RNAs from the RNA-seq of human brain tissue-derived small EVs. **C**. A Venn diagram of overlapping brain-derived 45 RNAs across different studies. **D**. Overlapping 45 genes are shown here.

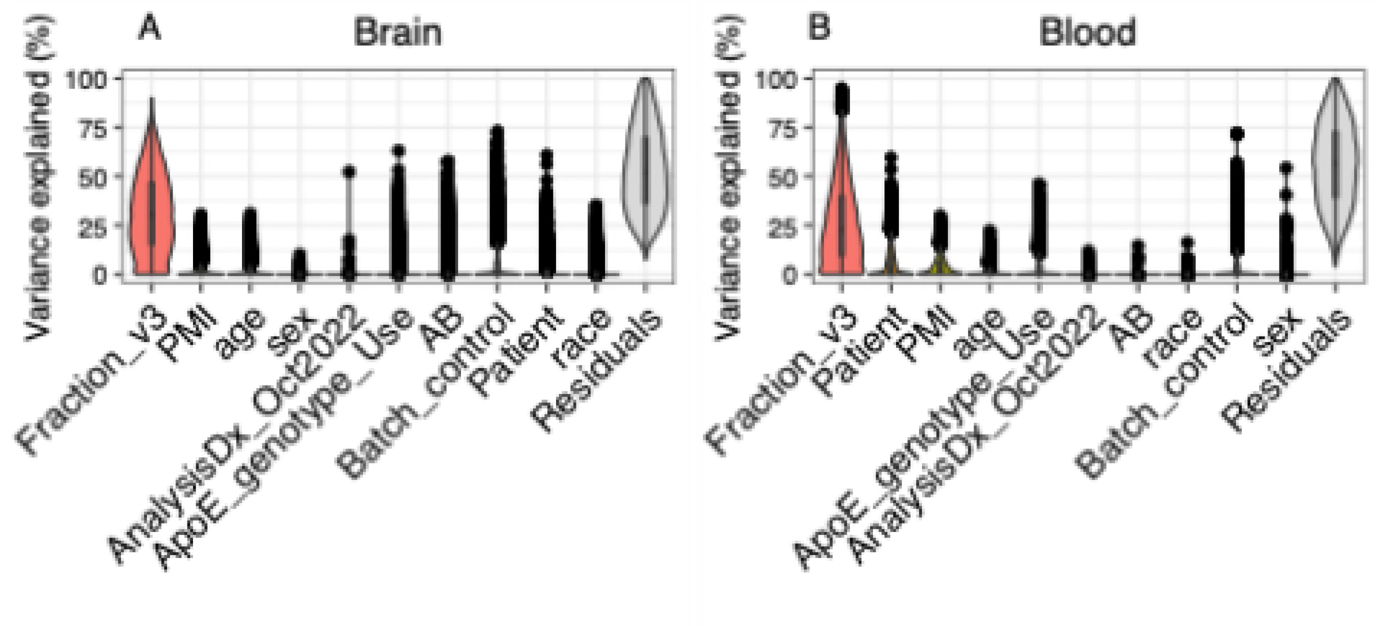

Supplementary Figure 10. Variance analyses shows that EVP subtype (labeled as "fraction_v3") displayed the highest variance contribution in brain and blood.

AD_Dx	Provenance	age	poE genotype
Ctrl_1	Autopsy	54	e3/e3
AD_1	Autopsy	82	e3/e3
Ctrl_2	Autopsy	99	e3/e4
AD_2	Autopsy	80	e3/e3
AD_3	Autopsy	76	e3/e3
Ctrl_3	Autopsy	67	e3/e3
AD_4	Autopsy	84	
Other_1	Autopsy	63	e3/e3
Other_2	Autopsy	68	e2/e3
AD_5	Autopsy	78	e3/e3
Ctrl_4	Autopsy	65	e3/e3
Other_3	Biopsy	58	other
Ctrl_5	Biopsy	80	other
Ctrl_6	Biopsy	49	other
Ctrl_7	Biopsy	62	other
Ctrl_8	Biopsy	58	other
AD_6	Biopsy	74	other
Other_4	Biopsy	28	other
Control_9	Autopsy	na	other
AD	Autopsy	na	other
AD	Autopsy	na	other
AD	Autopsy	na	other
Control	Autopsy	na	other
Control	Autopsy	na	other
Control	Autopsy	na	other
AD	Autopsy	na	other
sAD	C-derived neu	68	not reported
sAD1	iPSC-derived	60	not reported
sAD2	iPSC-derived	70	not reported
sAD3	iPSC-derived	62	not reported
sAD4	iPSC-derived	72	not reported
sAD5	iPSC-derived	55	not reported
sAD6	iPSC-derived	70	not reported
sAD7	iPSC-derived	81	not reported
Control1	iPSC-derived	56	not reported
Control2	iPSC-derived	81	not reported
Control3	iPSC-derived	85	not reported
Control4	iPSC-derived	56	not reported
Control5	iPSC-derived	88	not reported
Control6	iPSC-derived	84	not reported
AD1	Autopsy	60+	E2/3
AD2	Autopsy	60+	E2/3
AD3	Autopsy	60+	E2/3
AD4	Autopsy	80+	E2/3
AD5	Autopsy	60+	E2/3
AD6	Autopsy	80+	E3/3
AD7	Autopsy	100+	E3/3
AD8	Autopsy	80+	E3/3
AD9	Autopsy	60+	E3/3
AD10	Autopsy	80+	E3/3
AD11	Autopsy	50+	E3/4
AD12	Autopsy	90+	E3/4
AD13	Autopsy	80+	E3/4
AD14	Autopsy	80+	E3/4
AD15	Autopsy	60+	E3/4
AD16	Autopsy	80+	E3/4
AD17	Autopsy	60+	E4/4
AD18	Autopsy	90+	E4/4
AD19	Autopsy	70+	E4/4
AD20	Autopsy	90+	E4/4
AD21	Autopsy	60+	E4/4
AD22	Autopsy	70+	E4/4

Extended data. Table 2. Patient sample information.
