# Supplementary material for: SECmeres outperform extracellular vesicles as potential blood RNA biomarkers for Alzheimer’s disease
Source: Nat Commun. 2026 Jun 22;17:5453. doi: 10.1038/s41467-026-74541-8 (PMC13287576; doi:10.1038/s41467-026-74541-8)
Supplement: Supplementary file 2 — Reporting Summary [file 41467_2026_74541_MOESM2_ESM.pdf]

nature portfolio

Corresponding author(s): Navneet Dogra, PhD, Panos Roussos, MD, PhD

Last updated by author(s): Apr 17, 2026

Reporting Summary

Nature Portfolio wishes to improve the reproducibility of the work that we publish. This form provides structure for consistency and transparency in reporting. For further information on Nature Portfolio policies, see our Editorial Policies and the Editorial Policy Checklist.

Statistics

For all statistical analyses, confirm that the following items are present in the figure legend, table legend, main text, or Methods section.

n/a	Confirmed	
☐	☒	The exact sample size (*n*) for each experimental group/condition, given as a discrete number and unit of measurement
☐	☒	A statement on whether measurements were taken from distinct samples or whether the same sample was measured repeatedly
☐	☒	The statistical test(s) used AND whether they are one- or two-sided<br>*Only common tests should be described solely by name; describe more complex techniques in the Methods section.*
☐	☒	A description of all covariates tested
☐	☒	A description of any assumptions or corrections, such as tests of normality and adjustment for multiple comparisons
☐	☒	A full description of the statistical parameters including central tendency (e.g. means) or other basic estimates (e.g. regression coefficient) AND variation (e.g. standard deviation) or associated estimates of uncertainty (e.g. confidence intervals)
☐	☒	For null hypothesis testing, the test statistic (e.g. *F*, *t*, *r*) with confidence intervals, effect sizes, degrees of freedom and *P* value noted<br>*Give P values as exact values whenever suitable.*
☒	☐	For Bayesian analysis, information on the choice of priors and Markov chain Monte Carlo settings
☒	☐	For hierarchical and complex designs, identification of the appropriate level for tests and full reporting of outcomes
☒	☐	Estimates of effect sizes (e.g. Cohen's *d*, Pearson's *r*), indicating how they were calculated

Our web collection on statistics for biologists contains articles on many of the points above.

Software and code

Policy information about availability of computer code

Data collection	not applicable
Data analysis	This paper does not report original code. The code<br>used is available on https://eegk.github.io/ or upon request.<br>All analysis were performed on<br>the R studio platform using R Version 4.1 and several packages including limma, edgeR, ggplot2, reshape,<br>tidyverse, variancePartition, pheatmap, annotables, GenomicFeatures, GenomicRanges and doParallel.

For manuscripts utilizing custom algorithms or software that are central to the research but not yet described in published literature, software must be made available to editors and reviewers. We strongly encourage code deposition in a community repository (e.g. GitHub). See the Nature Portfolio guidelines for submitting code & software for further information.

Data

Policy information about availability of data

All manuscripts must include a data availability statement. This statement should provide the following information, where applicable:

- Accession codes, unique identifiers, or web links for publicly available datasets
- A description of any restrictions on data availability
- For clinical datasets or third party data, please ensure that the statement adheres to our policy

Data
reported in this paper has been shared by the lead contact. Raw RNA-seq data will be available upon publication at https://www.ncbi.nlm.nih.gov/geo/, which can be searched with GEO number (to be provided).Protocols for EV separation and characterization are provided with this manuscript and will be made available on EV TRACK (http://evtrack.org/) upon publication of the manuscript. Data reported in this paper has been shared by the lead contact. This paper does not report original code. The code used is available on https://eegk.github.io/ or upon request. RNA-seq data will be available upon publication at https://www.ncbi.nlm.nih.gov/geo/, which can be searched with GEO number (to be provided). Any additional information required to reanalyze the data reported in this paper is available from the lead contact upon request.

Research involving human participants, their data, or biological material

Policy information about studies with human participants or human data. See also policy information about sex, gender (identity/presentation), and sexual orientation and race, ethnicity and racism.

Reporting on sex and gender	Sex based analyses were performed.
Reporting on race, ethnicity, or other socially relevant groupings	To account for various co-facotors, we applied variance partition analyses (as described in methods). A comprehensive dataset of different confounding factors (age, race, ApoE genotype, sex, temporary/final diagnosis, cause of death, lobe, diabetes, smoking, depression, hypertension, early vs late onset of AD) was available and taken into consideration.
Population characteristics	APOE genotype were considered for the study.
Recruitment	The samples were obtained from already established bio bank.from the Mount Sinai NIH Brain Bank and Tissue Repository
Ethics oversight	The brain bank procedures are approved by the Icahn School of Medicine at Mount Sinai Institutional Review Board (IRB). We confirm that our research complies with all relevant ethical regulations and are approved by the Icahn School of Medicine at Mount Sinai Institutional Review Board (IRB). Informed consent was obtained from participants at the time of original sample collection.

Note that full information on the approval of the study protocol must also be provided in the manuscript.

Field-specific reporting

Please select the one below that is the best fit for your research. If you are not sure, read the appropriate sections before making your selection.

[x] Life sciences [ ] Behavioural & social sciences [ ] Ecological, evolutionary & environmental sciences

For a reference copy of the document with all sections, see nature.com/documents/nr-reporting-summary-flat.pdf

Life sciences study design

All studies must disclose on these points even when the disclosure is negative.

Sample size	A power analysis was used to determine the appropriate sample size.
Data exclusions	No data were excluded from the study.
Replication	We have conducted reproducibility analyses of our SECrifuge methodology and its comparison with existing technology ultracentrifugation (UC) using additional set of 10 human subjects (5 AD, 5 controls). We have conducted transcriptomic analyses of additional 10 subjects (5 AD and 5 controls) EVPs isolated using SECrifuge methodology.
Randomization	Data was randomized. To address if the observed differences are driven by a subset of individuals rather than reflecting a consistent trend, we analyzed individual subsets (in three different batches) rather than keeping them as a single batch. We applied boorstrapping, a non-parametric statistical method. Bootstrapping estimates how reliable our result is by repeatedly resampling our data and re-computing the statistic of interest. From these analyses, we identified top significant gene differentially expression between AD and controls.
Blinding	In our reproducibility experiment, we blinded the patient groups and isolated EVPs using SECrifuge. We found that

large EVs, small EVs, and small EPs were reproducibly isolated ($P < 0.00005$)

Reporting for specific materials, systems and methods

We require information from authors about some types of materials, experimental systems and methods used in many studies. Here, indicate whether each material, system or method listed is relevant to your study. If you are not sure if a list item applies to your research, read the appropriate section before selecting a response.

Materials & experimental systems

n/a	Involved in the study
☒	☐ Antibodies
☒	☐ Eukaryotic cell lines
☒	☐ Palaeontology and archaeology
☒	☐ Animals and other organisms
☐	☒ Clinical data
☒	☐ Dual use research of concern
☒	☐ Plants

Methods

n/a	Involved in the study
☐	☐ ChIP-seq
☐	☐ Flow cytometry
☐	☐ MRI-based neuroimaging

Clinical data

Policy information about clinical studies

All manuscripts should comply with the ICMJE guidelines for publication of clinical research and a completed CONSORT checklist must be included with all submissions.

Clinical trial registration	NA
Study protocol	NA
Data collection	NA
Outcomes	NA

Plants

Seed stocks	NA
Novel plant genotypes	NA
Authentication	NA

ChIP-seq

Data deposition

☐ Confirm that both raw and final processed data have been deposited in a public database such as GEO.

☐ Confirm that you have deposited or provided access to graph files (e.g. BED files) for the called peaks.

Data access links<br>*May remain private before publication.*	NA
Files in database submission	NA
Genome browser session<br>(e.g. UCSC)	NA

Methodology

Replicates	NA

Sequencing depth: NA

Antibodies: NA

Peak calling parameters: NA

Data quality: NA

Software: NA

Flow Cytometry

Plots

Confirm that:

- [ ] The axis labels state the marker and fluorochrome used (e.g. CD4-FITC).
- [ ] The axis scales are clearly visible. Include numbers along axes only for bottom left plot of group (a 'group' is an analysis of identical markers).
- [ ] All plots are contour plots with outliers or pseudocolor plots.
- [ ] A numerical value for number of cells or percentage (with statistics) is provided.

Methodology

Sample preparation: NA

Instrument: NA

Software: NA

Cell population abundance: NA

Gating strategy: NA

- [ ] Tick this box to confirm that a figure exemplifying the gating strategy is provided in the Supplementary Information.

Magnetic resonance imaging

Experimental design

Design type: NA

Design specifications: NA

Behavioral performance measures: NA

Acquisition

Imaging type(s): NA

Field strength: NA

Sequence & imaging parameters: NA

Area of acquisition: NA

Diffusion MRI [ ] Used [x] Not used

Preprocessing

Preprocessing software: NA

Normalization: NA

Normalization template: NA

Noise and artifact removal: NA

Volume censoring: NA

Statistical modeling & inference

Model type and settings	NA
Effect(s) tested	NA

Specify type of analysis: ☐ Whole brain ☐ ROI-based ☐ Both

Statistic type for inference (See Eklund et al. 2016)	NA
Correction	NA

Models & analysis

n/a	Involved in the study
☒	☐ Functional and/or effective connectivity
☒	☐ Graph analysis
☒	☐ Multivariate modeling or predictive analysis
