# Supplementary material for: SECmeres outperform extracellular vesicles as potential blood RNA biomarkers for Alzheimer’s disease
Source: Nat Commun. 2026 Jun 22;17:5453. doi: 10.1038/s41467-026-74541-8 (PMC13287576; doi:10.1038/s41467-026-74541-8)
Supplement: Supplementary file 3 — Transparent Peer Review file [file 41467_2026_74541_MOESM3_ESM.pdf]

nature portfolio

Peer Review File

SECmeres Outperform Extracellular Vesicles as Potential Blood RNA Biomarkers for Alzheimer's Disease

Corresponding Author: Dr navneet dogra

This file contains all reviewer reports in order by version, followed by all author rebuttals in order by version.

Version 0:

Reviewer comments:

Reviewer #1

(Remarks to the Author)
Thank you for the opportunity to review this interesting manuscript that presents initial results on a new technology. Overall, the impressive technology and promising results need to be balanced against very small sample sets that are inadequate to support multiple conclusions. Without evaluation in an appropriately sized cohort of living individuals well annotated as cases and controls, which are readily available from multiple national resources, this potentially important new technology is not yet matured beyond proof-of-concept stage. I have the following specific comments:

I applaud the novel SECrifuge technique as a potential advance in a somewhat bewildering field of competing technologies. However, only 3 samples from uncharacterized individuals is inadequate to establish the correspondence between SECrifuge-derived EVPs and the most commonly used existing methods and taxonomy. A more robust demonstration in control and AD samples is needed to establish confidence that SECrifuge-derived data is concordant with existing standard protocols. Otherwise these results stand alone with unknown generalizability in an already complex field.

Similarly, only 3 uncharacterized individuals' samples to establish "distinct" proteins in different subpopulations of EVPs seems to a very strong conclusion from a very limited number of human samples (Figure 3). Are all 3 people adults? Men or women? Age? Perhaps this is sufficient as a proof-of-concept methods paper but is inadequate to support the claims being made. Samples underlying Figure 4 are from only 4 cases and 4 controls; the same concern applies to the conclusion of "distinct" RNA content.

The number of subjects in the neuropathology section (10 AD and 16 control) is reasonable given the limitations of this type of study and seems to be appropriately controlled for confounding variables, although the racial variation in the cohort is quite limited beyond Caucasian. The samples appear to derive from biopsy or from autopsy. This opens many important issues. What is the impact of general anesthesia that presumably all patients received who underwent brain biopsy? What was the reason for brain biopsy and could this be a confounder? When comparing biopsy results to autopsy results, what was the impact of dying? Extended table 2 is confusing because there are only 18 entries yet this section of the paper claims 26 individuals with autopsy or biopsy. Regardless, this is still a very small numbers to claim to have a significant classifier without some sort of validation in CSF or blood from living individuals. Samples from expertly annotated living cases and controls are available from multiple national resources and the investigators' institutions would remove this last very serious concern. Omission of data from samples sourced from clinical research cohorts is a major shortcoming that seriously undermines the potential impact of this new approach to EVPs.

Reviewer #2

(Remarks to the Author)
This study presents a comprehensive profiling of extracellular particles in Alzheimer's disease (AD) and identifies a novel class of small extracellular particles (sEPs) as potential biomarkers, representing a valuable contribution to the field. However, several key issues should be addressed to strengthen the validity and interpretation of the findings

Major Comments
1. The authors apply different false discovery rate (FDR) thresholds without justification. In some cases, the threshold is set relatively high (FDR < 0.2), which may raise concerns for EVP analyses, particularly given the known reproducibility challenges in extracellular vesicle and particle studies.
2. While the study examines extracellular particles derived from both brain and blood samples, it does not provide a direct comparison between these two tissue sources. It remains unclear how the same molecular species (sEVs, sEPs, lEVs) compare across tissues and whether the observed molecular signatures are reproducible across compartments.
3.“Finally, we curated a list of brain-specific, and in many cases, brain-exclusive protein markers (Extended Data Table 1) from the Human Protein Atlas, GTEx Portal, and Allen Brain Atlas 48,49,228. Over 95% of the selected proteins in our curated list were exclusively enriched in brain, reflecting brain lineage-specific markers (Extended Data Table 1).”
Please clarify the criteria used to distinguish “brain-specific” from “brain-exclusive” proteins.
4. The use of single-nucleus RNA sequencing data to infer the cellular origin of extracellular particles assumes that the nuclear transcriptome accurately reflects RNA packaged into EVs, which is typically sorted from the cytoplasm. This assumption should be discussed.
5. The gene list in Figure 6G differs from those in Figures 6D–E. The criteria for gene selection in Figure 6G should be explicitly described.
6. In Figure 6G, the control group exhibits substantial heterogeneity, suggesting that observed differences may be driven by a subset of individuals rather than reflecting a consistent trend.
7. In Extended Data Figure 7, it is unclear how correlations between Braak stage, CERAD score, and identified biomarkers were evaluated. Additionally, information regarding the patients’ neuropathological diagnoses is missing.
Minor Comments
• Please add or clarify the colormap legend for Figures 2d–e to ensure consistency and ease of interpretation.
• Typo: “whereases”

Version 2:

Reviewer comments:

Reviewer #1

(Remarks to the Author)
I thank the reviewers for a highly responsive revision that improves the quality of data and analyses. Indeed, I think the fundamentals of this new technique are demonstrated robustly but I remain cautious about the claimed molecular distinction of patients vs. controls based on an enlarged but still relatively small set of independent samples. The field has experienced such strong initial claims many times only to learn later that the situation was more nuanced.

1. My suggestion is to make clear that although the sample size is adequate for discovery, much more work needs to be done regarding influence of race, gender, age, stage of Alzheimer’s disease, and the influence of uncharacterized co-morbidities variably present in the autopsy samples. The potential impacts of confounding by underlying disease that promoted brain biopsy and by general anesthesia also remain unknown for biopsy samples.

2. I suspect that there is an error in the supplementary table that describes characteristics of people who made brain donations. Post-mortem interval for the autopsy samples from one source ranges from 241 min to 1750 min and from a second source ranges from 4 min to 20 min. I expect that the units are incorrect for tissue from the second source and that PMI is 4 hr to 20 hr rather than min. Converting PMI from the first tissue source’s data to hr then has their PMI ranging from 4 to 29 hr, conveniently overlapping between the two sources if my assumption is correct. If I am wrong, then there is a major systematic difference in the autopsy specimens from different sources.

3. With this sample size and variation in PMI, I suggest that the authors conduct supplemental analyses on the effect of PMI on the major outcomes and report these in the text. I recognize that the authors have done variance analyses but these PMIs are broad and some longer than is typical for RNA-based detection.

Reviewer #2

(Remarks to the Author)
The authors have addressed all of my comments and concerns, and the revised manuscript has improved accordingly. I have no further comments.

Reviewer #3

(Remarks to the Author)
In this manuscript, the authors show that a previously overlooked class of sub-50nm extracellular nanoparticles (small EPs), serves as a more effective non-invasive biomarker for Alzheimer’s Disease (AD) than traditional small extracellular vesicles (sEVs).

The researchers identified these nanoparticles in both human brain tissue and blood. Notably, they lack the protein hallmarks typically found in exosomes and sEVs but are highly enriched with brain-specific markers. In addition, RNA associated with small EPs can discriminate AD patients and healthy controls. Using single-nucleus RNA sequencing

deconvolution, the authors mapped the origins of these particles and associate small EVs with RNA from oligodendrocytes and astrocytes and small EPs with brain-endothelial cells, critical components of the blood-brain barrier.

Specific Comments:
1) It is unclear how the authors excluded contamination from intracellular vesicles during the isolation of brain-derived EVs/EPs. Given the complexity of brain tissue, more detail is needed on how vesicles released extracellularly were distinguished from intracellular vesicles originating from disrupted cells. Please clarify the strategy and provide evidence that intracellular contamination is minimal.

2) The EM images are generally unfocused and do not convincingly support the reported size distributions. In particular, the images of blood-derived EVs are of very low informative value and do not add meaningful support to the conclusions. Higher-quality EM data would be necessary.

3) Regarding the analysis of total RNA content in each EV/EP population, it is unclear how normalization was performed. Was RNA normalized to EV number, EV size, total protein, or another parameter? This is critical for interpreting differences in cargo composition.

4) The manuscript emphasizes fundamental cargo differences between EV/EP classes, potentially reflecting distinct biogenetic pathways. However, further sub-fractionation by size could reveal additional heterogeneity, as EV size may correspond to distinct functional subpopulations depending on cell type and biogenesis mechanisms.

5) Do the brain-specific transcript signatures include microglial markers? This should be clarified, as microglia represent a major brain-resident cell population and could significantly influence the interpretation of brain-derived EV cargo.

6) Markers typically associated with exomeres are missing for the sEP population. Please clarify whether these were assessed and, if not, discuss this limitation.

7) Extended Data Fig. 2A–F does not appear essential to support the main conclusions and could potentially be removed.

8) In Fig. 2D, CD63 is missing. Please clarify whether CD63 was assessed.

9) The color codes used in Figures 1–3 are confusing, not only across different figures but even within the same panel. A consistent color scheme would greatly improve readability.

Version 3:

Reviewer comments:

Reviewer #1

(Remarks to the Author)
Thank you for the thoughtful revisions. I have no further recommendations.

Reviewer #3

(Remarks to the Author)
I have carefully evaluated the revised version of the manuscript as well as the authors’ responses to my comments. The authors have thoroughly addressed all the points I raised and have made appropriate revisions to the manuscript. I am satisfied with the changes implemented and believe that the revised version has improved.

Open Access This Peer Review File is licensed under a Creative Commons Attribution 4.0 International License, which permits use, sharing, adaptation, distribution and reproduction in any medium or format, as long as you give appropriate credit to the original author(s) and the source, provide a link to the Creative Commons license, and indicate if changes were made.
In cases where reviewers are anonymous, credit should be given to 'Anonymous Referee' and the source.
The images or other third party material in this Peer Review File are included in the article's Creative Commons license, unless indicated otherwise in a credit line to the material. If material is not included in the article's Creative Commons license and your intended use is not permitted by statutory regulation or exceeds the permitted use, you will need to obtain permission directly from the copyright holder.
To view a copy of this license, visit https://creativecommons.org/licenses/by/4.0/

We are grateful to the Editor for allowing us to address the reviewer's feedback, which has greatly improved this manuscript. We also thank the reviewers, as their critical input has positively impacted the quality of our manuscript. We now clarify the important points raised by reviewers. We address in detail each question below:
Reviewer's feedback is highlighted in blue text and our responses are in green.

Reviewer's Comments:

Reviewer #1 (Remarks to the Author)
Reviewer Comment: Thank you for the opportunity to review this interesting manuscript that presents initial results on a new technology. Overall, the impressive technology and promising results need to be balanced against very small sample sets that are inadequate to support multiple conclusions. Without evaluation in an appropriately sized cohort of living individuals well annotated as cases and controls, which are readily available from multiple national resources, this potentially important new technology is not yet matured beyond proof-of-concept stage.
Response: Dear Reviewer#1, thank you for constructive comments and time spent to provide important feedback, which has helped us improve our manuscript. Additionally, we are highly appreciative of the Reviewer for recognizing the value of our *SECrifuge* methodology and providing encouraging remarks ("*I applaud the novel SECrifuge technique*"). Also, we acknowledge Reviewer's concern about the low sample number and the resulting data supporting our conclusions. We assure you that we are dedicated to improving our empirical evidence and have conducted additional experiments to support our conclusion or adding limitations of the study. As suggested, we have now made following modifications in the main manuscript and included new complementary data in support of conclusions:

- We have now conducted new reproducibility analyses of our SECrifuge methodology and its comparison with existing technology ultracentrifugation (UC) using additional set of 10 human subjects (5 AD, 5 controls).
- We have now conducted new transcriptomic analyses of additional 10 subjects (5 AD and 5 controls) EVPs isolated using SECrifuge methodology.
- New transcriptomics from 10 brain tissue homogenates has now been included. This new dataset improved the brain lineage marker analysis.
- Figures 1 has been modified. Figures. 1N, O, P, Q now include new reproducibility (in size, and RNAseq) data and comparison of SECrifuge with UC. Additionally, Extended data Fig. 1F now includes reproducibility analyses of Brain-derived RNA among EVPs isolated from different human subject's serum.
- Figures 2 has been modified. Fig. 2F. now includes reproducibility (in proteins) analyses and relative comparison of tetraspanins concentrations in large EVs, small EVs, and small EPs. Fig. 2G. shows relative concentrations of tetraspanins between AD and controls.
- Figure 3 has been modified. Fig. 3A, B now includes additional brain and blood EVP's proteomic datasets.
- Figure 4 has been modified. Fig. 4 now includes additional RNAseq datasets and their corresponding analyses.
- A new Extended data Figure 5 has been included. This figure shows reproducible enrichment of the RNA molecules within each EVP subtype (large EVs, small EVs, and small EPs) from brain and blood of 10 human subjects (5 AD and 5 controls).
- Figure 6 have been modified. Figure 6H now shows significant brain and blood EVP-associated RNAs. Additionally, methods section now details the analyses carried out to obtain Fig. 6G.
- A new Figure 7 has been added. This figure clearly displayed the significant RNAs and their association with different EVPs.

- As suggested, we have now compared our findings (AD versus controls) with living individuals derived iPSC neurons.
- A new Extended data Figure 9 has been included. Here, the variance analyses shows that EVP subtype displayed the highest variance contribution in brain and blood.
- A new Extended data Figure 10 has been included. The new figure shows Overlapping RNAs across studies (Fig. 10A-d). 10A. shows top differential RNAs from the RNAseq of Small EVs isolated from human iPSCs neurons (AD and healthy donors(HD)). 10B shows Top differential RNAs from the RNAseq of human brain tissue-derived small EVs. Fig. 10C shows a Venn diagram of overlapping brain-derived 45 RNAs across different studies. Fig. 9D. shows the list of overlapping 45 genes across studies.
- To address EVP's brain cell of origin, we have analyzed and compared brain tissue RNAseq with EVP RNAseq from matching subjects.
- As suggested, we have substantially increased the size of our patient cohort to 62 samples (40 AD and 22 controls).
- Patient sample information table has been updated.
- New additions are highlighted in the revised manuscript and clean copy has been provided for your convenience.

Below we address specific comments provided by the Reviewer.
I have the following specific comments:
I applaud the novel SECrifuge technique as a potential advance in a somewhat bewildering field of competing technologies.
We are grateful for your applause, encouragement, and acknowledging the potential advance of our *SECrifuge* methodology. Thanks to a new NIH funding (details below), we truly believe that this novel technology can shift the current paradigm of EV isolation.

However, only 3 samples from uncharacterized individuals is inadequate to establish the correspondence between SECrifuge-derived EVPs and the most commonly used existing methods and taxonomy. A more robust demonstration in control and AD samples is needed to establish confidence that SECrifuge-derived data is concordant with existing standard protocols. Otherwise these results stand alone with unknown generalizability in an already complex field.
We acknowledge the concern about the strength of the data supporting our conclusions. We assure you that we are dedicated to improving our empirical evidence and plan to conduct additional experiments. We agree that we need to establish confidence that SECrifuge is reproducible and is concordant with existing standard protocols.

As suggested by the reviewer, and to ensure rigor and reproducibility of SECrifuge, we have

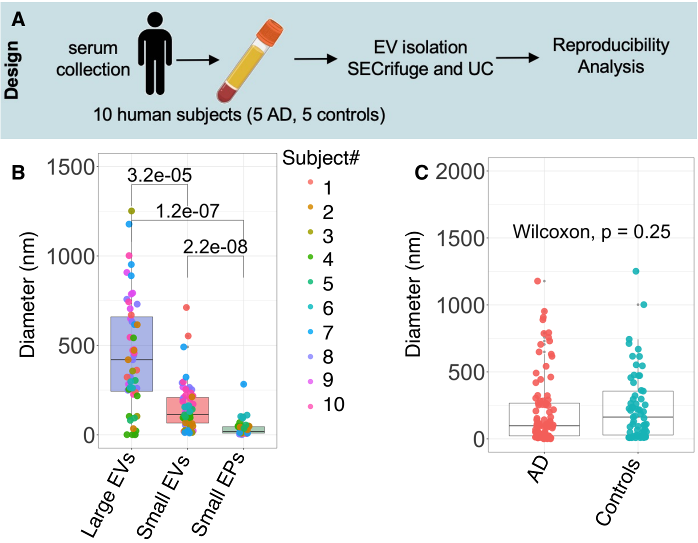

Response Fig. 1. Reproducible separation of EVPs. A. Reproducible ($P < 0.00005$) separation of large EVs, small EVs, and small EPs in 10 human subjects (5 AD and 5 controls). The dots are colored by each individual subject. Significant P-value is observed for all EVPs. P-values were calculated using a two-sided Welch's t-test, which compares group means while accounting for unequal variances. **B**. EVPs isolated from AD and controls had no significant differences in their diameters (nm). The dots are colored by AD and controls. Because the data did not meet assumptions of normality, comparisons between AD and control groups were performed using the non-parametric Wilcoxon rank-sum test.

tested our methodology on a new set of 10 human subjects (5 AD, 5 controls) and in vitro cell lines. The datasets are described below as follows: **1**) We tested the reproducibility of EVP isolation with their size and cargo (RNA and proteins). **2**) We conducted a comparative RNA sequencing (RNAseq) analysis of SECrifuge and ultracentrifugation (UC) isolated EVs. **3**) We tested our methodology for reproducible separation of EVPs in in vitro cell models.

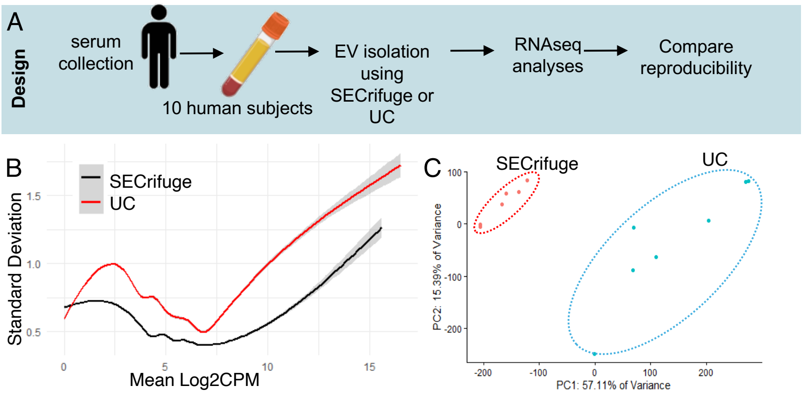

Response Figure 2. Comparative RNA sequencing analysis of SECrifuge and UC-isolated EVs. **A.** Experimental flow chart for RNA sequencing of EVs isolated from serum derived from a 10 human subjects EV isolation followed by RNA extraction and library preparation. **B** Plot of standard deviation versus mean $\log_2$ counts per million (CPM) for RNA sequencing data from 9 SECrifuge and UC EV isolates. **C**. PCA for RNA sequencing data from UC and SECrifuge isolates.

<u>**1**) Testing the reproducible separation of EVPs by size</u>: Reproducibility of EVP's (large EVs, small EVs, and small EPs) isolation was assessed using repeated independent isolations. DLS measurements showed consistent particle size distributions and low variability across replicates, indicating high reproducibility of the isolation method. We tested isolation reproducibility of SECrifuge methodology with additional set of 10 human subjects (5 AD, 5 controls). In this new experiment, we blinded the patient groups and isolated EVPs using SECrifuge. We found that large EVs, small EVs, and small EPs were reproducibly isolated ($P < 0.00005$) (Response Fig. 1A). Additionally, as suggested by the reviewer, we compared size of EVP from AD and control subjects. We found that there was no significant difference in the size of EVPs by disease condition (Response Fig. 1B). For your convenience, the new figures are shown here (response figure 1 A & B) and have been added to the main manuscript (Fig. 1).

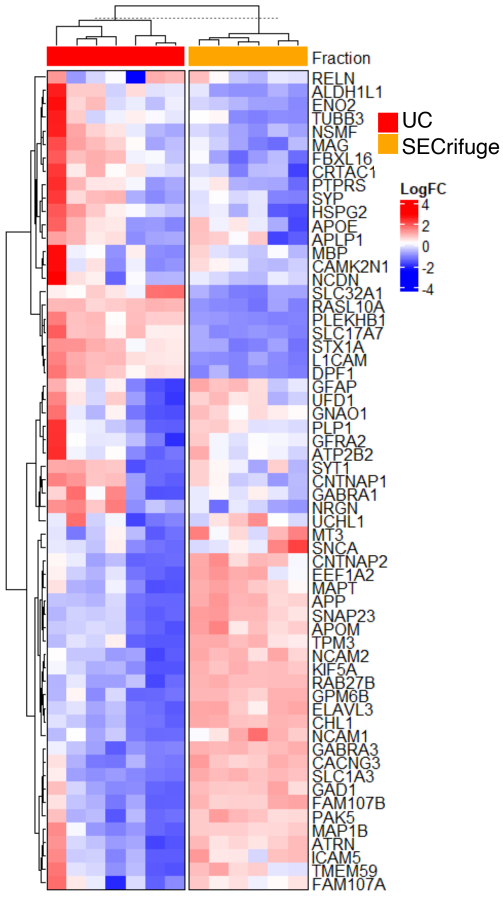

Response Figure 3. Reproducibility of Brain-derived RNA among different human subjects. RNAseq analysis of SECrifuge and UC-isolated EVs. Brain-derived RNAs are compared among each separate isolation.

<u>**2**) Comparison with other exiting methods and reproducible RNA analyses of EVPs</u>: In this study, we conducted a comparative RNAseq analysis of SECrifuge and ultracentrifugation (UC) isolated EVs. To measure variability, we isolated EVs by UC and by SECrifuge respectively (Response Fig. 2A). In our previous publications[1, 2], we have observed that variability depends on gene expression level, reflecting the heteroskedastic nature of the data. This dependence can be characterized by the relationship between the standard deviation σ vs. the mean μ (Response Fig. 2B) of replicate gene expression measurements. To compute this relationship, we bin genes by average expression and pooled gene expression values of all genes whose mean in the replicates fell within that bin. In this way, we can estimate the probability density function of the pooled gene expression values in each bin (Response Fig. 2B), which can be characterized by σ and μ of the replicate average

in that bin. The resulting σ vs. µ relations were fit by a local regression (LOESS) curve for each replicate (Response Fig. 2B.). SECrifuge replicates (n = 6) displayed low variability, while UC replicates (n = 7) introduce considerable additional variability, evidenced by a σ that increases with µ, indicating highly expressed genes detected at significantly different expression levels. This variability may be due to isolation of different EVPs together, hence the additional genetic sources, including larger vesicles, and small EPs during UC processing. These data imply that SECrifuge-isolated EVs yield more reproducible sequencing data than UC-isolated EVs. Principal components analysis (PCA) for RNAseq data from SECrifuge EV isolates displayed lower variance compared to UC (Response Fig. 2C).

<u>Identifying same RNA molecules in different isolations</u>. Here, we compared a list of common brain-derived RNA markers in each individual serum EVs (Response Fig. 3). SECrifuge demonstrated reproducible identification of brain derived markers in all six samples, while lower samples to sample reproducibility was observed between each UC isolation (Response Fig. 3).

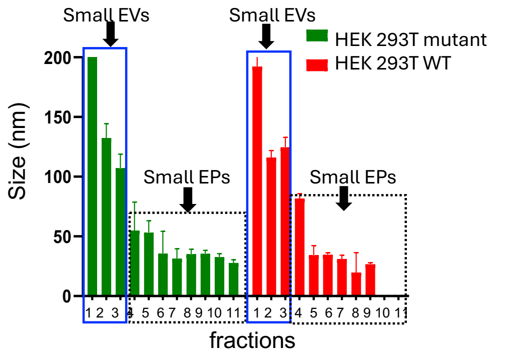

Response Figure 4. EVP isolation in other cell models. Two distinct cell models were cultured independently, and EVPs were isolated using SECrifuge.

<u>**3**) Reproducible separation of EVPs in other cell models:</u> While this data is beyond the scope of this manuscript, we wish to share with the reviewer that our methodology is reproduced in other biofluids, disease models, and cell culture derived EVPs. In another ongoing project in the lab, two distinct human embryonic kidney (HEK) cell models (wild type and mutant) were cultured independently, and EVPs were isolated using SECrifuge (Response fig. 4). The goal of this project is to isolate small EVs and small EPs. Using SECrifuge we were able to reproducibly isolate EVPs in less than one hour.

Finally, we wish to share that we (the corresponding author and co-authors) were recently awarded a new NIH R01 (R01CA296581) grant, where our SECrifuge methodology will be used for EVP isolation from >100 human subject's blood serum. Consequently, we are continuing to test and improve our SECrifuge methodology. In summary, our new experiments reassure the reproducible isolation and characterization of EVPs isolated using SECrifuge and comparison with existing technologies.

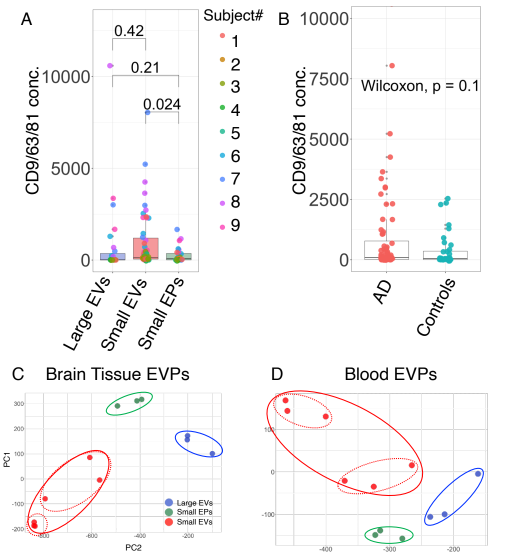

Response Figure 5. Protein quantification and comparison. A. Relative comparison of tetraspanins in large EVs, small EVs, and small EPs. **B**. Relative expression of tetraspanins between AD and controls. **C**. Principal components analysis (PCA) of brain-derived EVP-proteome. **D**. PCA of blood-derived EVP-proteome.

Similarly, only 3 uncharacterized individuals' samples to establish "distinct" proteins in different subpopulations of EVPs seems to a very strong conclusion from a very limited number of human samples (Figure 3). Are all 3 people adults? Men or women? Age? Perhaps this is sufficient as a proof-of-concept methods paper but is inadequate to support the claims being made.

As suggested by the reviewer, we have now expanded these analyses to include 10 human subjects (5 AD and 5 controls). Here, we compared Tetraspanins (CD9, CD63, CD81) — considered markers for small EVs[3]. We found that while the large EV and small EV Displayed non-significant differences in Tetraspanins, small EPs were significantly (P = 0.024) depleted in tetraspanins (Response Fig. 5A). We then compared the relative

expression of tetraspanins between AD and controls. We found that there was no significant (P = 0.1) difference in tetraspanin enrichment between AD and controls (Response Fig. 5B).

Additionally, we have now conducted additional 6 subject's proteomic analyses from AD and controls. The new data has been incorporated in the main manuscript Fig 3. And is displayed here (Response Fig for your convenience. Additionally, thanks to the reviewer's feedback, we have added additional donor meta data, including age, race and APOE genotype (table 1).

As suggested, we have toned down our claims and our analysis as preliminary results and "*proof of concept*". We have adjusted the text to reflect a more moderate language, the revised paragraph now says: "*while this study provides a proof-of-concept and preliminary analyses of a small set of samples, different EVPs seem to have different cargo types*."

As a result of these new analyses, we have modified the Figure 3 in the main manuscript. Figure 3 is also displayed below for your convenience.

Samples underlying Figure 4 are from only 4 cases and 4 controls; the same concern applies to the conclusion of "distinct" RNA content.

As suggested by the reviewer, we have now expanded these transcriptomic analyses to 26 human subjects (10 AD and 16 controls). We were reassured that all 26 subject displayed distinct enrichment of the RNA within each EVP subtype (large EVs, small EVs, and small EPs) (Response Fig. 6). The new data has been incorporated in the main manuscript Fig 4. And is displayed here (Response Fig. 6 for your convenience.

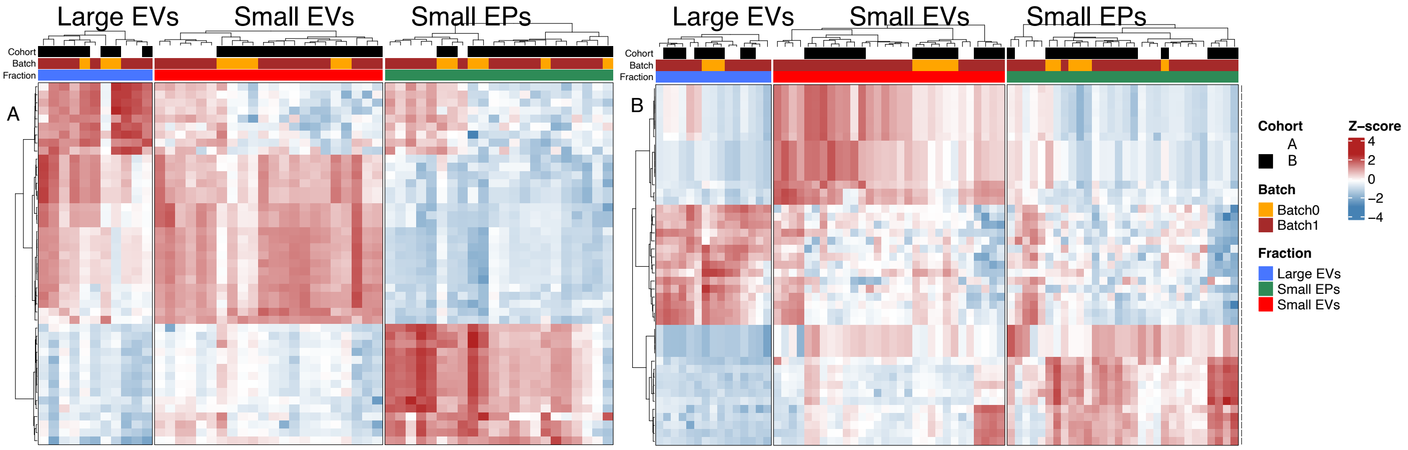

Response Figure 6. Enrichment of the RNA within EVP subtype (large EVs, small EVs, and small EPs) from brain and blood. **A**. Brain EVPs isolated from 11 human subjects. **B** Blood EVPs isolated from 11 human subjects. A and B are two different cohorts of subjects. Batch 0 and Batch 1 are two different sequencing batched of EVPs isolated from human subjects. EVPs are labeled as fractions.

The number of subjects in the neuropathology section (10 AD and 16 control) is reasonable given the limitations of this type of study and seems to be appropriately controlled for confounding variables, although the racial variation in the cohort is quite limited beyond Caucasian.

We are thankful for this comment and agree that racial variation is limited. We have added a "limitations" section where the following text has been added: "*we acknowledge the limited number of racial variations in our samples and, as a consequence, our claims were restricted to Caucasians due to the limited set of samples.*"

The samples appear to derive from biopsy or from autopsy. This opens many important issues. What is the impact of general anesthesia that presumably all patients received who underwent brain biopsy?

What was the reason for brain biopsy and could this be a confounder? When comparing biopsy results to autopsy results, what was the impact of dying?

Thank you for highlighting this concern. These analyses in fact improved our reproducibility and helped us a ton by removing a major cofactor. We agree that biopsy and autopsy are inherently different and could be a confounder. As per reviewer's suggestion, we conducted a separate analysis, where biopsies were not included in AD vs controls analyses (previously presented in Fig. 6). In these new analyses, we removed 7 biopsy samples (1AD, 6 controls), and conducted analyses only on the 19 subjects (9 AD and 10 controls). We found that AD and controls demonstrated unique RNAs enriched in small EVs (Response Fig. 7A) and small EPs (Response Fig. 7B).

To identify if autopsy is indeed a confounder in our analysis, we conducted variance partition analysis. This analysis addressed how much of the variability in our data is explained by each factor. In this study, we used a mixed effect model to account for the difference between these cohorts. We found that cohort (combined autopsy and biopsy) contributed to less variability than other cofactors (Response fig. 7C, total variance contribution by autopsy samples is highlighted red). We have added a supplementary figure to show these differences.

We have now presented biopsy and autopsy as a confounder (Fig. 6G, CKs1 are biopsies, while VAs0 and VAs1 are autopsies). Furthermore, we have added a "*limitations*" section where the following text has been added: "*we acknowledge that the biopsies samples may not truly represent controls.*"

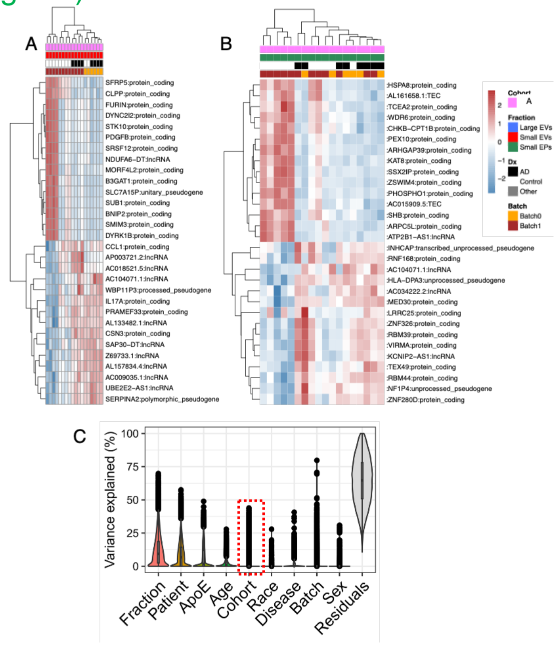

Response Figure 7. Autopsy as a confounder. Comparing AD and controls from Blood small EVs (A) and small EPs (B). **C**. The total variance contribution by autopsy samples is highlighted red.

The biopsies are from patients with edema who underwent a procedure to clear the fluid - an artifact of which is removal of a small piece of cortex which we used for our studies. The brain bank procedures are approved by the Icahn School of Medicine at Mount Sinai Institutional Review Board (IRB).

We've looked at differences between living and dead tissue and will likely need input from one of the computational people to best address the reviewer's comment.

Extended table 2 is confusing because there are only 18 entries yet this section of the paper claims 26 individuals with autopsy or biopsy.

We apologize for this oversight. This error has now been fixed. The new Extended table 2 has been added to the manuscript.

Regardless, this is still a very small numbers to claim to have a significant classifier without some sort of validation in CSF or blood from living individuals. Samples from expertly annotated living cases and controls are available from multiple national resources and the investigators' institutions would remove this last very serious concern. Omission of data from samples sourced from clinical research cohorts is a major shortcoming that seriously undermines the potential impact of this new approach to EVPs.

Thank you for highlighting this critical point about reproducibility and small number of samples. To ensure that our findings provide an unbiased outcome that is not dependent upon small number, EVP isolation methods, and is reproducible across sites/institutions, and protocols applied for RNA-seq, we

have increased our QC reproducibility, and RNAseq reproducibility in our own datasets and curated external datasets from Johns Hopkins University (details provided in revised Extended data table 2). Our combined analyses have included new brain homogenates, and iPSCs from living subjects (total samples 62, with 40 AD and 22 controls) presented a set of overlapping 45 brain-derived RNA molecules which were represented across experiments, batches, and institutions (Response Fig. 8). Thanks to the reviewer's feedback, we are now able to provide a set of RNA biomarkers, which are tested in inter-institutional variability. We have prepared a new figure (Extended Fig. 10), which is also provided here for your convenience.

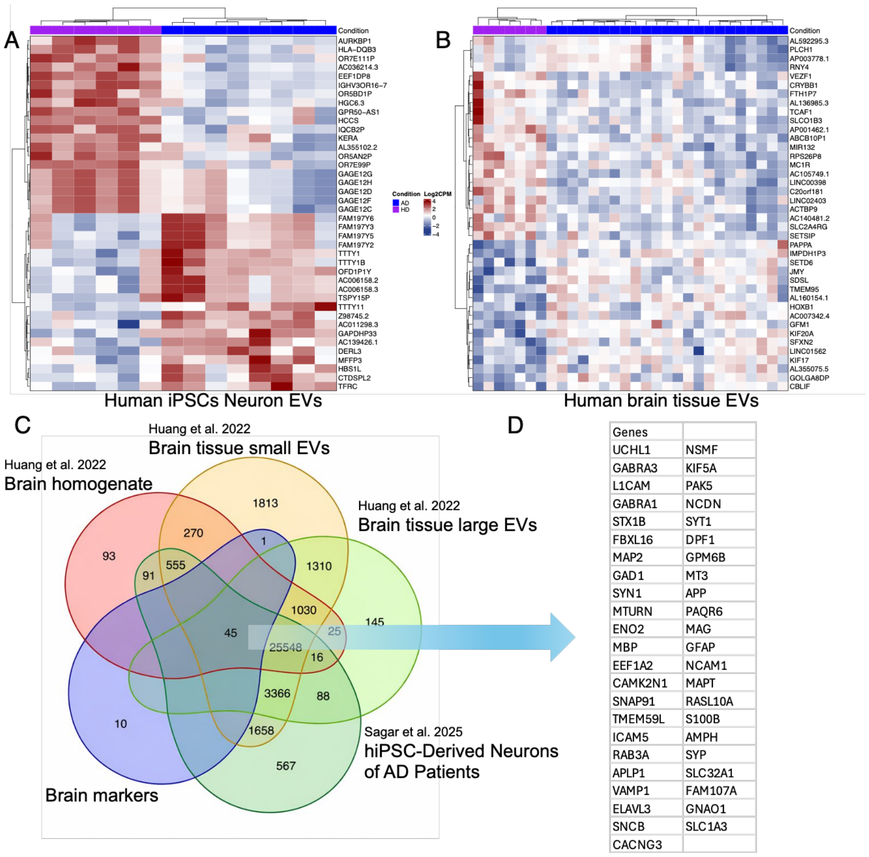

Response Figure 8. Overlapping RNAs across studies. A. Top differential RNAs from the RNAseq of Small EVs isolated from human iPSCs neurons (AD and healthy donors(HD)). **B**. Top differential RNAs from the RNAseq of human brain tissue-derived small EVs. **C.** A Venn diagram of overlapping brain-derived 45 RNAs across different studies. **D**. Overlapping 45 genes are shown here.

Finally, we are thankful to the Reviewer for their time, several positive comments, and important feedback that has made this manuscript much stronger. The new data created enhances the reproducibility and robustness of our study, We are hopeful that our response is satisfactory and that this manuscript will be an excellent resource for the EVP and AD community. Thank you!

Reviewer #2 (Remarks to the Author)

Reviewer Comment: This study presents a comprehensive profiling of extracellular particles in Alzheimer's disease (AD) and identifies a novel class of small extracellular particles (sEPs) as potential biomarkers, representing a valuable contribution to the field. However, several key issues should be addressed to strengthen the validity and interpretation of the findings

Response: Dear Reviewer #2, Thank you for your valuable insights and constructive feedback on our manuscript regarding small extracellular particles (EPs) in Alzheimer's disease (AD). We thank the opportunity to address the concerns raised. We are appreciative of the Reviewer for recognizing the value of our study, "*a valuable contribution to the field*". Also, we acknowledge the concern about the strength and reproducibly of the data supporting our conclusions. We assure you that we are dedicated to improving our empirical evidence and have conducted additional experiments to support our claims. As suggested, we have now made following modifications in the main manuscript and included new complementary data:

- We have now conducted new reproducibility analyses of our SECrifuge methodology and its comparison with existing technology ultracentrifugation (UC) using additional set of 10 human subjects (5 AD, 5 controls).
- We have now conducted new transcriptomic analyses of additional 10 subjects (5 AD and 5 controls) EVPs isolated using SECrifuge methodology.
- New transcriptomics from 10 brain tissue homogenates has now been included. This new dataset improved the brain lineage marker analysis.
- Figures 1 has been modified. Figures. 1N, O, P, Q now include new reproducibility (in size, and RNAseq) data and comparison of SECrifuge with UC. Additionally, Extended data Fig. 1F now includes reproducibility analyses of Brain-derived RNA among EVPs isolated from different human subject's serum.
- Figures 2 has been modified. Fig. 2F. now includes reproducibility (in proteins) analyses and relative comparison of tetraspanins concentrations in large EVs, small EVs, and small EPs. Fig. 2G. shows relative concentrations of tetraspanins between AD and controls.
- Figure 3 has been modified. Fig. 3A, B now includes additional brain and blood EVP's proteomic datasets.
- Figure 4 has been modified. Fig. 4 now includes additional RNAseq datasets and their corresponding analyses.
- A new Extended data Figure 5 has been included. This figure shows reproducible enrichment of the RNA molecules within each EVP subtype (large EVs, small EVs, and small EPs) from brain and blood of 10 human subjects (5 AD and 5 controls).
- Figure 6 have been modified. Figure 6H now shows significant brain and blood EVP-associated RNAs. Additionally, methods section now details the analyses carried out to obtain Fig. 6G.
- A new Figure 7 has been added. This figure clearly displayed the significant RNAs and their association with different EVPs.
- As suggested, we have now compared our findings (AD versus controls) with living individuals derived iPSC neurons.
- A new Extended data Figure 9 has been included. Here, the variance analyses shows that EVP subtype displayed the highest variance contribution in brain and blood.
- A new Extended data Figure 10 has been included. The new figure shows Overlapping RNAs across studies (Fig. 10A-d). 10A. shows top differential RNAs from the RNAseq of Small EVs isolated from human iPSCs neurons (AD and healthy donors(HD)). 10B shows Top differential RNAs from the RNAseq of human brain tissue-derived small EVs. Fig. 10C shows a Venn

diagram of overlapping brain-derived 45 RNAs across different studies. Fig. 9D. shows the list of overlapping 45 genes across studies.

- To address EVP's brain cell of origin, we have analyzed and compared brain tissue RNAseq with EVP RNAseq from matching subjects.
- As suggested, we have substantially increased the size of our patient cohort to 62 samples (40 AD and 22 controls).
- Patient sample information table has been updated.
- New additions are highlighted in the revised manuscript and clean copy has been provided for your convenience.

Below we address specific comments provided by the Reviewer.

Major Comments

1. The authors apply different false discovery rate (FDR) thresholds without justification. In some cases, the threshold is set relatively high (FDR < 0.2), which may raise concerns for EVP analyses, particularly given the known reproducibility challenges in extracellular vesicle and particle studies.

Thank you, this question overlaps with Reviewer 1's comment regarding reproducibility of EVP isolation and analyses. We therefore refer to Figure R1-2 (reproduced here for your convenience):

We acknowledge the concern about the reproducibility challenges in extracellular vesicle and particle studies. We assure you that we are dedicated to improving our empirical evidence and have conducted additional experiments. Below we discuss the new experiments and additional reproducibility analyses. This strategy has helped us reduce false discovery rates (FDR), thereby increasing reproducibility:

As suggested by the reviewer, and to ensure rigor and reproducibility of SECrifuge, we have tested our methodology on a new set of 10 human subjects (5 AD, 5 controls) and in vitro cell lines. The datasets are described below as follows: **1**) We tested the reproducibility of EVP isolation with their size and cargo (RNA and proteins). **2**) We conducted a comparative RNA sequencing (RNAseq) analysis of SECrifuge and ultracentrifugation (UC) isolated EVs. **3**) We tested our methodology for reproducible separation of EVPs in in vitro cell models.

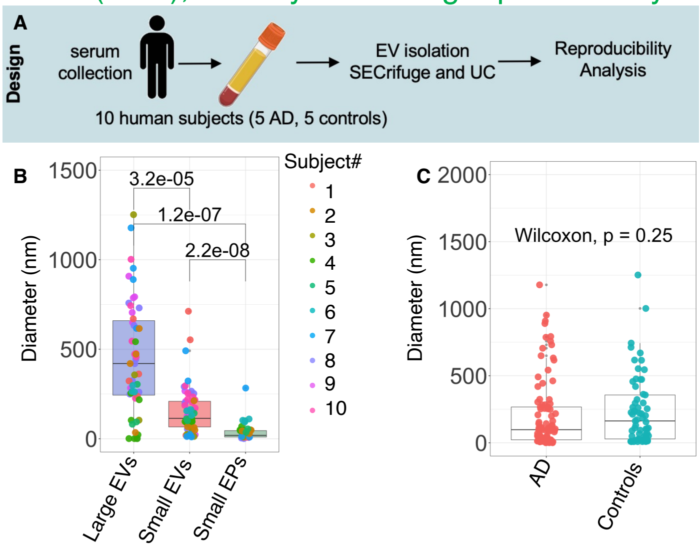

Response Fig. 1. Reproducible separation of EVPs. A. Reproducible ($P < 0.00005$) separation of large EVs, small EVs, and small EPs in 10 human subjects (6 AD and 5 controls). The dots are colored by each individual subject. Significant P-value is observed for all EVPs. P-values were calculated using a two-sided Welch's t-test, which compares group means while accounting for unequal variances. **B**. EVPs isolated from AD and controls had no significant differences in their diameters (nm). The dots are colored by AD and controls. Because the data did not meet assumptions of normality, comparisons between AD and control groups were performed using the non-parametric Wilcoxon rank-sum test.

<u>1) Testing the reproducible separation of EVPs by size</u>: Reproducibility of EVP's (large EVs, small EVs, and small EPs) isolation was assessed using repeated independent isolations. DLS measurements showed consistent particle size distributions and low variability across replicates, indicating high reproducibility of the isolation method. We tested isolation reproducibility of SECrifuge methodology with additional set of 10 human subjects (5 AD, 5 controls). In this new experiment, we blinded the patient groups and isolated EVPs using SECrifuge. We found that large EVs, small EVs, and small EPs were reproducibly isolated (P <

0.00005) (Response Fig. 1A). Additionally, as suggested by the reviewer, we compared size of EVP from AD and control subjects. We found that there was no significant difference in the size of EVPs by disease condition (Response Fig. 1B). For your convenience, the new figures are shown here (response figure 1 A & B) and have been added to the main manuscript (Fig. 1).

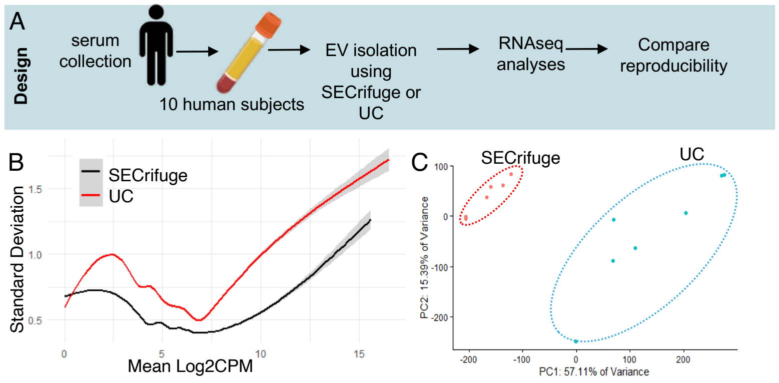

Response Figure 2. Comparative RNA sequencing analysis of SECrifuge and UC-isolated EVs. A. Experimental flow chart for RNA sequencing of EVs isolated from serum derived from a 10 human subjects EV isolation followed by RNA extraction and library preparation. **B** Plot of standard deviation versus mean $\log_2$ counts per million (CPM) for RNA sequencing data from 9 SECrifuge and UC EV isolates. **C**. PCA for RNA sequencing data from UC and SECrifuge isolates.

<u>2) Comparison with other exiting methods and reproducible RNA analyses of EVPs</u>: In this study, we conducted a comparative RNAseq analysis of SECrifuge and ultracentrifugation (UC) isolated EVs. To measure variability, we isolated EVs by UC and by SECrifuge respectively (Response Fig. 2A). In our previous publications[1, 4], we have observed that variability depends on gene expression level, reflecting the heteroskedastic nature of the data. This dependence can be characterized by the relationship between the standard deviation σ vs. the mean μ (Response Fig. 2B) of replicate gene expression measurements. To compute this relationship, we bin genes by average expression and pooled gene expression values of all genes whose mean in the replicates fell within that bin. In this way, we can estimate the probability density function of the pooled gene expression values in each bin (Response Fig. 2B), which can be characterized by σ and μ of the replicate average in that bin. The resulting σ vs. μ relations were fit by a local regression (LOESS) curve for each replicate (Response Fig. 2B.). SECrifuge replicates (n = 6) displayed low variability, while UC replicates (n = 7) introduce considerable additional variability, evidenced by a σ that increases with μ, indicating highly expressed genes detected at significantly different expression levels. This variability may be due to isolation of different EVPs together, hence the additional genetic sources, including larger vesicles, and small EPs during UC processing. These data imply that SECrifuge-isolated EVs yield more reproducible sequencing data than UC-isolated EVs. Principal components analysis (PCA) for RNAseq data from SECrifuge EV isolates displayed lower variance compared to UC (Response Fig. 2C).

<u>Identifying same RNA molecules in different isolations</u>. Here, we compared a list of common brain-derived RNA markers in each individual serum EVs (Response Fig. 3). SECrifuge demonstrated reproducible identification of brain derived markers in all six samples, while lower samples to sample reproducibility was observed between each UC isolation (Response Fig. 3).

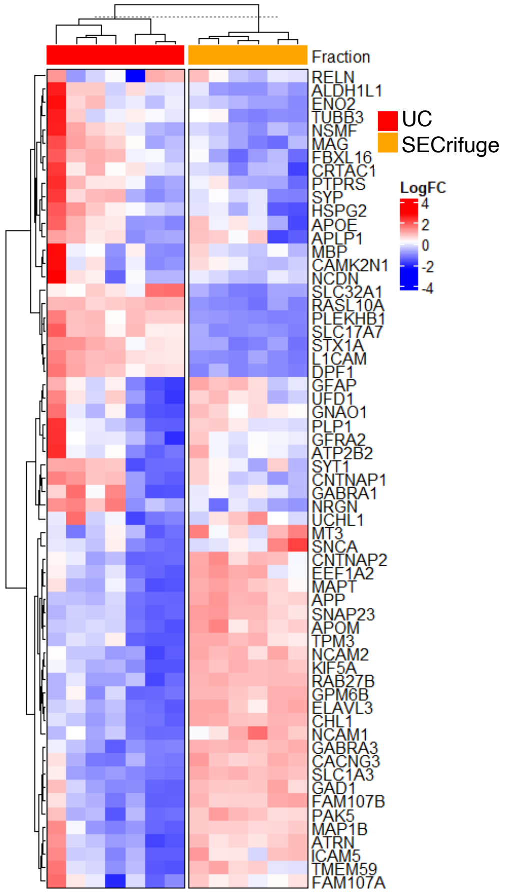

Response Figure 3. Reproducibility of Brain-derived RNA among different human subjects. RNAseq analysis of SECrifuge and UC-isolated EVs. Brain-derived RNAs are compared among each separate isolation.

3) <u>Reproducible separation of EVPs in other cell models:</u> While this data is beyond the scope of this manuscript, we wish to share with the reviewer that our methodology is reproduced in other biofluids, disease models, and cell culture derived EVPs. In another ongoing project in the lab, two distinct human embryonic kidney (HEK) cell models (wild type and mutant) were cultured independently, and EVPs were isolated using SECrifuge (Response fig. 4). The goal of this project is to isolate small EVs and small EPs. Using SECrifuge we were able to reproducibly isolate EVPs in less than one hour.

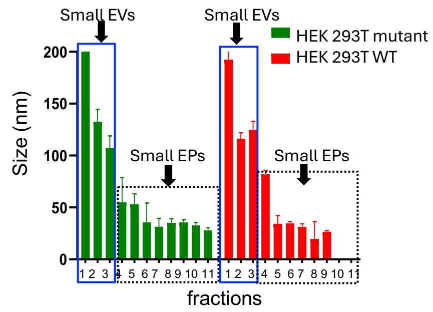

Response Figure 4. EVP isolation in other cell models. Two distinct cell models were cultured independently, and EVPs were isolated using SECrifuge.

Finally, we wish to share that we (the corresponding author and co-authors) were recently awarded a new NIH R01 (R01CA296581) grant, where our SECrifuge methodology will be used for EVP isolation from >100 human subject's blood serum. Consequently, we are continuing to test and improve our SECrifuge methodology.
In summary, our new experiments reassure the reproducible isolation and characterization of EVPs isolated using SECrifuge and comparison with existing technologies.

2. While the study examines extracellular particles derived from both brain and blood samples, it does not provide a direct comparison between these two tissue sources. It remains unclear how the same molecular species (sEVs, sEPs, lEVs) compare across tissues and whether the observed molecular signatures are reproducible across compartments.
Thank you for your valuable suggestions. We have included a direct comparison between the two sources. We have also included a direct quantitative comparison of molecular signatures across EVP subtypes derived from brain and blood (Response Fig. 9). This figure has been added to the extended data figure 7. The quantitative comparison of molecular signatures across EVP subtypes derived from brain and blood has been added to Fig. 6H in the main manuscript. The figures are displayed below for your convenience.

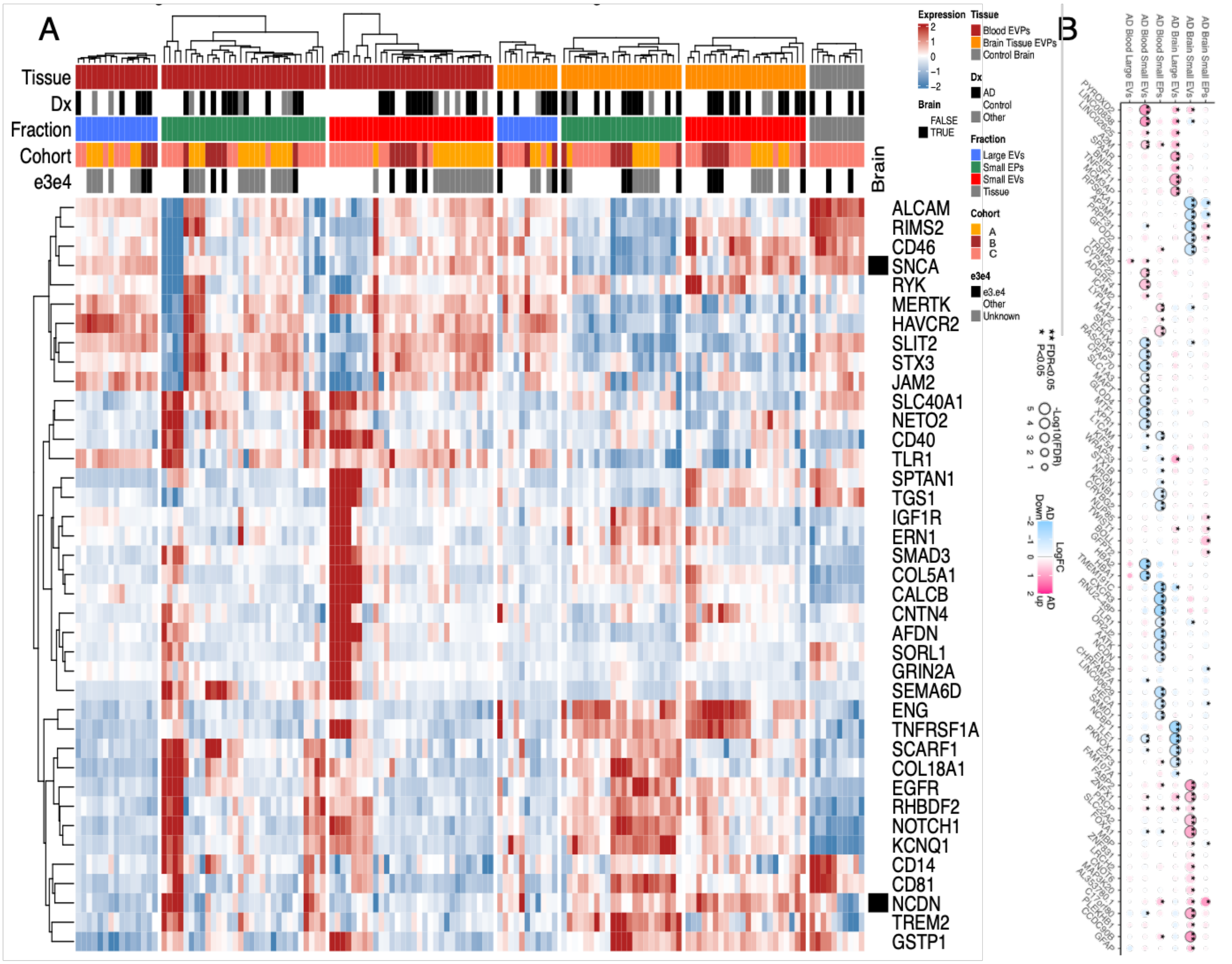

Response Figure 9. **A**. Brain and blood-derived EVPs RNA from AD and non-AD cases. **B**. This correlation figure provides a direct quantitative comparison of molecular signatures across EVP subtypes derived from brain and blood.

Additional Correlations: By evaluating the correlation structure of RNA profiles between matched EV/EP classes (sEVs, sEPs, and lEVs) across compartments, this analysis assesses the degree of concordance between tissue sources. Demonstrating positive and subtype-consistent correlations supports the conclusion that each particle class carries a reproducible molecular identity, independent of its anatomical origin. This new analysis therefore shows the evidence that comparable extracellular particle populations exist in both tissues and that their transcriptome-level features are preserved across biological compartments.

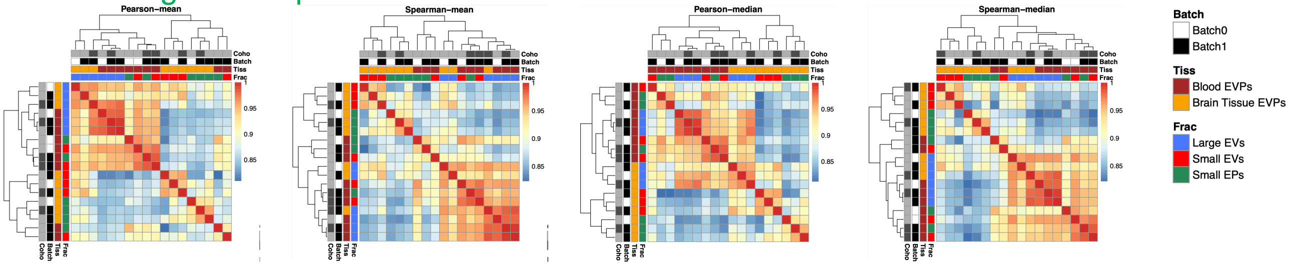

Pearson Mean   Spearman Mean   Pearson Median   Spearman Median   legends

Response Figure 10. Evidence that comparable EVP populations exist in both tissues and that their transcriptome-level features are preserved across biological compartments.

Additional Differential Expression: The supplemental differential expression (DE) figure expands on this by determining whether the same genes that distinguish particle subtypes within one type (e.g., blood) exhibit similar directionality and magnitude of change in the corresponding subtypes derived from brain tissue. Differential expression shows that distinct signatures were present across different compartments (The gene list for each EVP is provided with the manuscript). By presenting cross-tissue concordance in DE signatures, we can evaluate the reproducibility of subtype-specific markers and the stability of biologically meaningful differences. Demonstrating consistent DE patterns across compartments strengthens the mechanistic interpretation that these particles share conserved biogenesis pathways and functional roles (Response fig. 11). This figure therefore directly addresses concerns regarding whether the molecular signatures identified in blood-derived particles accurately reflect those present in the brain.

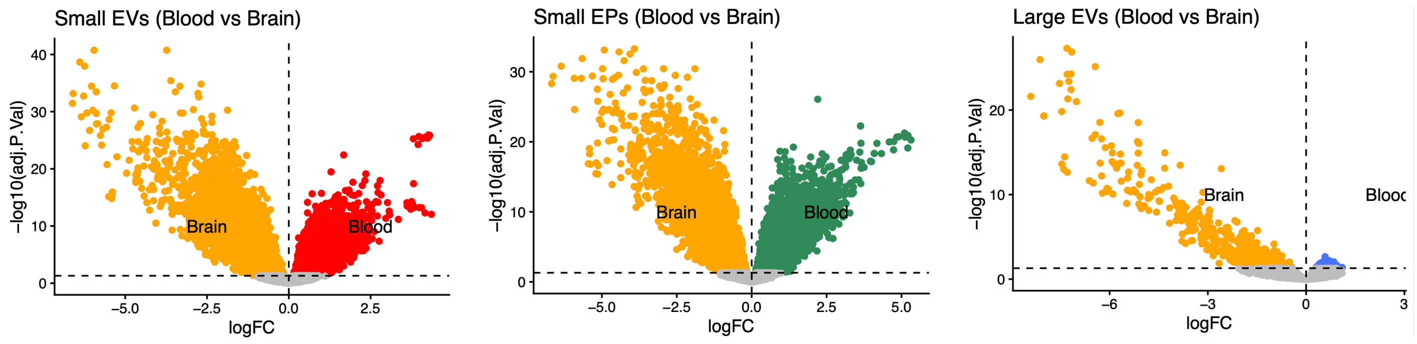

Response Figure 11. Differential expression shows distinct signatures across different compartments. The gene list for each EVP is provided with the manuscript.

Variance analyses: By comparing variance structures across compartments, this figure helps determine whether observed differences are driven by consistent biological signal or by high inter-individual noise in one tissue relative to another. We found that EVP subtype (labeled as "fraction_v3") displayed the highest variance explained in brain and blood (response Fig. 12). Demonstrating similar variance profiles across brain- and blood-derived particles provides an additional layer of evidence that the molecular features of each subtype are robust, transferable, and not artificially inflated by tissue-specific heterogeneity.

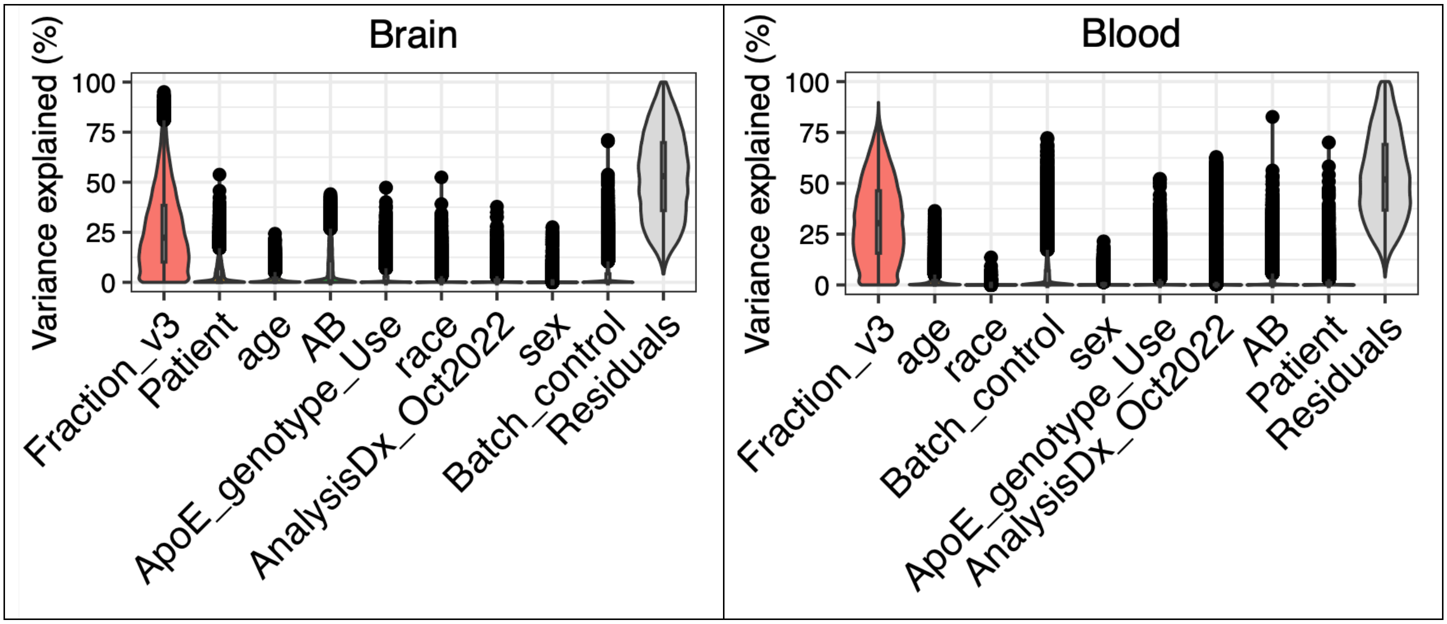

Response Figure 12. Variance analyses shows that EVP subtype (labeled as "fraction_v3") displayed the highest variance contribution in brain and blood.

Collectively, these variance-partition analyses show that the EV/EP subclass ("Fraction") is the dominant axis of variation in both brain and blood datasets, while technical and demographic covariates contribute minimally. The reproducibility of this pattern across compartments supports the premise that particle subclass-specific molecular signatures are robust, biologically grounded, and comparable between tissues. This directly addresses reviewer concerns regarding the cross-tissue consistency and biological validity of small EP, small EV, and large EV molecular profiles.
This figure has now been added as Extended data Figure 9. In the main manuscript.

3."Finally, we curated a list of brain-specific, and in many cases, brain-exclusive protein markers (Extended Data Table 1) from the Human Protein Atlas, GTEx Portal, and Allen Brain Atlas 48,49,228. Over 95% of the selected proteins in our curated list were exclusively enriched in brain, reflecting brain lineage-specific markers (Extended Data Table 1)."
Please clarify the criteria used to distinguish "brain-specific" from "brain-exclusive" proteins.
Thank you for highlighting this very important point. After considering you comment, we have changed the text "brain-exclusive" to brain-specific, as there is no true brain-exclusive but overwhelmingly brain-specific marker. Below we explain how such markers were curated.
Criteria to define brain-specificity: RNA lineage is specific to diverse tissue types[5]. To address their brain specificity, we carefully curated a list of commonly studied genes in the context of brain and compared their relative bulk tissue gene expression in GTEX (Response Fig. 13). Our criteria were, If the top 5 tissue types are brain-tissue subtypes then the gene is termed as brain-specific. While some genes have been investigated for brain derived EVs (such as L1CAM), but they were abundantly expressed in Colon, Esophagus, and Kidney (Response Fig. 13). On the other hand, GFAP RNA was overwhelmingly present in brain tissue subtypes, hence termed as highly specific brain gene (Response Fig. 13). We have added this information in methods section (titled as "Criteria to define brain-specificity") .

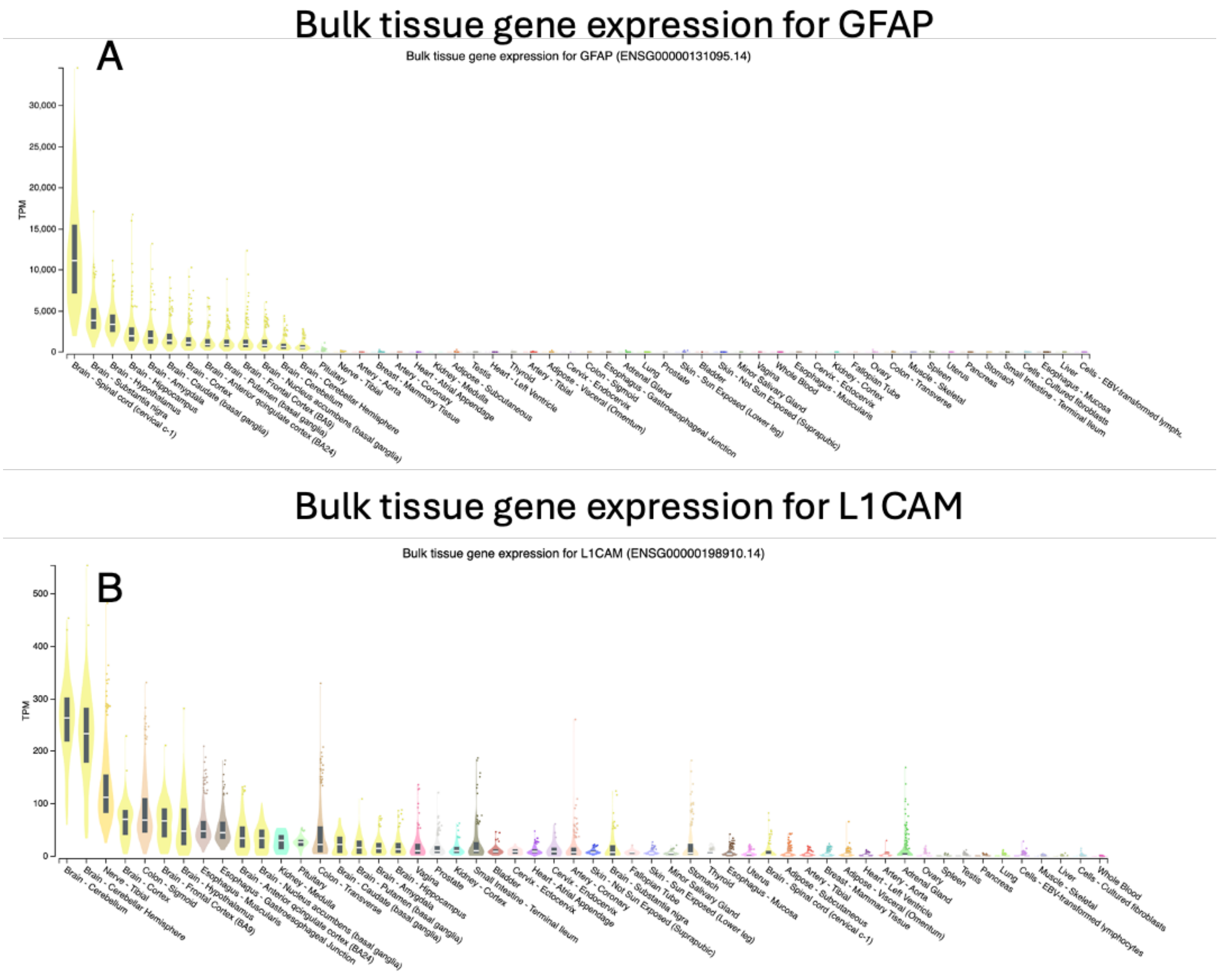

Response Figure 13. Brain-specificity of two gene studies in EVPs. While GFAP is highly specific, L1CAM was not.

4. The use of single-nucleus RNA sequencing data to infer the cellular origin of extracellular particles assumes that the nuclear transcriptome accurately reflects RNA packaged into EVs, which is typically sorted from the cytoplasm. This assumption should be discussed.

Thank you for this thoughtful comment. We agree that inferring the cellular origin of EVs from snRNA-seq data requires careful consideration because snRNA-seq captures nuclear rather than cytoplasmic transcripts. This approach assumes that nuclear gene expression patterns are a reasonable proxy for the broader programs that control which RNAs are packaged into EVs. While individual cytoplasmic transcripts may differ, the key cell type–defining and cell state–related expression signatures are well captured in nuclear RNA[6], supporting the use of snRNA-derived profiles for cell-type–level comparisons. Importantly, our analysis is designed to infer cell-type correspondence, not direct transcript-level equivalence between nuclear RNA and EV cargo. We acknowledge this as a limitation and view our framework as complementary to cytoplasmic EV-RNA profiling, with future single-cell approaches linking nuclear and EV-sorted RNA expected to further strengthen these inferences.

5. The gene list in Figure 6G differs from those in Figures 6D–E. The criteria for gene selection in Figure 6G should be explicitly described.

Thank you for highlighting the point. This was intentional but not clearly explained. Figures 6D–E presents the top differentially expressed genes as a mean of gene expression across all subjects, identified within each specified comparison. On the other hand, Figure 6G provides an integrated summary of established brain-specific genes that are both significantly altered and shared across the overlapping signatures. These analyses encompass multiple parallel comparisons, including EP-, small EV–, and large EV–derived blood samples, evaluated across three independent cohorts.
The criteria for gene selection in Figure 6G has now been described in Methods section and below for your convenience.

"*We applied boorstrapping to enhances the accuracy of differential analysis and the gene expression results providing a non-parametric, empirical approach to estimate variability, standard errors, confidence intervals, and p-values through repeated resampling with replacement from the original data. This reduces reliance on potentially misspecified parametric assumptions, leading to more robust and well-calibrated inferences, particularly in low-replicate scenarios or heterogeneous datasets common in omics. For differentials between batches (technical groupings like sequencing runs) and cohorts (biological groupings like patient populations or studies), we applied a specialized bootstrapping strategy: stratified resampling within batches and hierarchical resampling to respect nested structures (samples within donors or cohorts), to separate technical noise from biological signal. This improves reproducibility, controls false discovery rates more effectively, reduces bias from unmodeled batch effects or cohort heterogeneity, and increases statistical power and stability when comparing results across technical batches or biological cohorts, often yielding more reliable differential calls than standard parametric methods alone*."

6. In Figure 6G, the control group exhibits substantial heterogeneity, suggesting that observed differences may be driven by a subset of individuals rather than reflecting a consistent trend.
We appreciate the Reviewer for pointing out this very important observation. There indeed is a batch effect, as the total ~150 EVPs RNAseq were sequences in 3 different batches (shown as cohort A, B, C in Fig. 6G). To address if the observed differences are driven by a subset of individuals rather than reflecting a consistent trend, we analyzed individual subsets (in three different batches) rather than keeping them as a single batch (Response Fig. 14A, B, C). We applied boorstrapping, a non-parametric statistical method. Bootstrapping estimates how reliable our result is by repeatedly resampling our data and re-computing the statistic of interest. From these analyses, we identified top significant gene differentially expression between AD and controls (Fig. 6G, H).
Next, to ensure that our findings provide an unbiased outcome that is not dependent upon variability driven by subsets and is reproducible across sites/institutions, we have compared our analyses with a curated external dataset from Johns Hopkins University (details provided in revised Extended data table 2). Our combined analyses have included new brain homogenates, and iPSCs from living subjects (total samples 62, with 40 AD and 22 controls) presented a set of overlapping 45 brain-derived RNA molecules (Response Fig. 14C, D), which were represented across experiments, batches, and institutions.

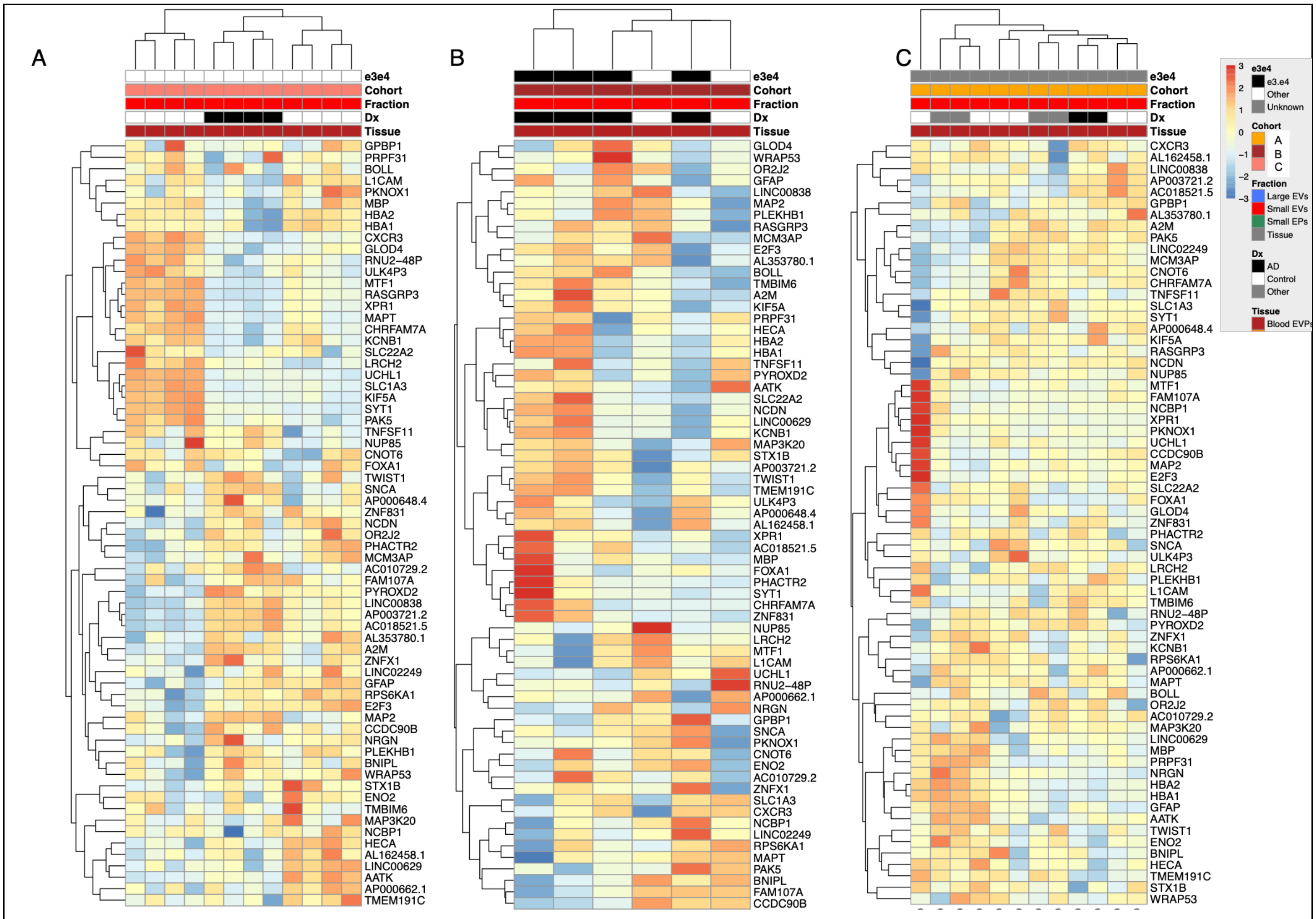

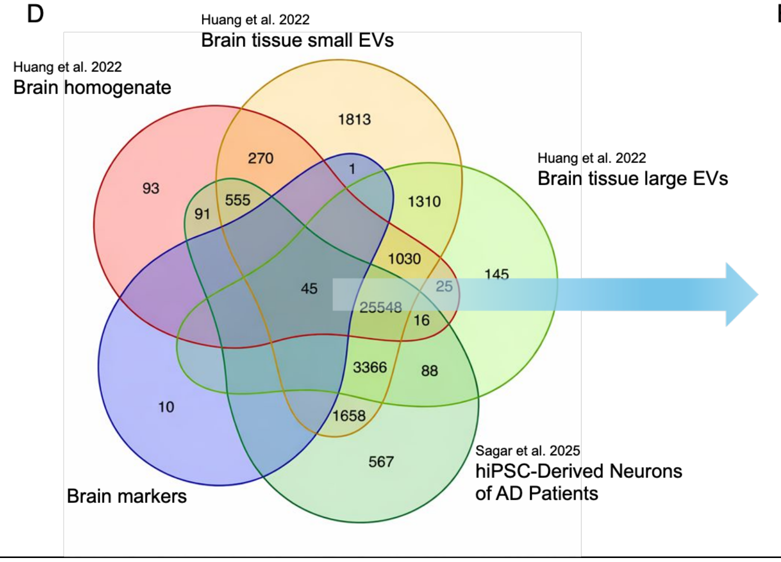

E

Common EVP RNAs across studies

Genes	
UCHL1	NSMF
GABRA3	KIF5A
L1CAM	PAK5
GABRA1	NCDN
STX1B	SYT1
FBXL16	DPF1
MAP2	GPM6B
GAD1	MT3
SYN1	APP
MTURN	PAQR6
ENO2	MAG
MBP	GFAP
EEF1A2	NCAM1
CAMK2N1	MAPT
SNAP91	RASL10A
TMEM59L	S100B
ICAM5	AMPH
RAB3A	SYP
APLP1	SLC32A1
VAMP1	FAM107A
ELAVL3	GNAO1
SNCB	SLC1A3
CACNG3	

Response Figure 14. A, B, C. RNAseq analyses of 3 different inhouse batches. **D**. Overlapping RNAs across studies. **E**. List of overlapping RNAs.

Thanks to the reviewer’s feedback, we are now able to provide a set of RNA biomarkers, which are tested in inter-institutional variability.

7. In Extended Data Figure 7, it is unclear how correlations between Braak stage, CERAD score, and identified biomarkers were evaluated. Additionally, information regarding the patients' neuropathological diagnoses is missing.

Thank you for the excellent question. The identification of Braak stage and CERAD was done by a collaborating lab using single cell analysis conducted by Dr. Donghoon Lee, at al.[7]. We only compared our differentially expressed genes to the genes identified by their team. We think this comparison is useful to the community because it shows that our signatures present in EVPs, can also be found associated with different brain pathologies and pathology based scores. We have coted this publication in the main manuscript.

Minor Comments

• Please add or clarify the colormap legend for Figures 2d–e to ensure consistency and ease of interpretation.

• Typo: “whereases”

Thank you for noticing these errors. We have fixed these errors.

Finally, we are thankful to the Reviewer for their time, several positive comments, and important feedback that has made this manuscript much stronger. The new data created enhances the reproducibility and robustness of our study, We are hopeful that our response is satisfactory and that this manuscript will be an excellent resource for the EVP and AD community. Thank you!

1. Chen, T.-Y. *et al.* Extracellular vesicles carry distinct proteo-transcriptomic signatures that are different from their cancer cell of origin. *iScience* **25** (2022).
2. Miceli, R.T. *et al.* Extracellular vesicles, RNA sequencing, and bioinformatic analyses: Challenges, solutions, and recommendations. *J Extracell Vesicles* **13**, e70005 (2024).
3. Welsh, J.A. *et al.* Minimal information for studies of extracellular vesicles (MISEV2023): From basic to advanced approaches. *J Extracell Vesicles* **13**, e12404 (2024).
4. Smith, J.T. *et al.* Integrated nanoscale deterministic lateral displacement arrays for separation of extracellular vesicles from clinically-relevant volumes of biological samples. *Lab Chip* **18**, 3913-3925 (2018).
5. Carithers, L.J. & Moore, H.M. The Genotype-Tissue Expression (GTEx) Project. *Biopreserv Biobank* **13**, 307-308 (2015).
6. Bakken, T.E. *et al.* Single-nucleus and single-cell transcriptomes compared in matched cortical cell types. *PLoS One* **13**, e0209648 (2018).
7. Lee, D. *et al.* Plasticity of Human Microglia and Brain Perivascular Macrophages in Aging and Alzheimer's Disease. *medRxiv* (2024).

We are grateful to the Editor for allowing us to address the reviewer's feedback, which has greatly improved this manuscript. We also thank the reviewers, as their valuable time and critical input has positively impacted the quality of our manuscript. We now clarify the important points raised by reviewers. We address in detail each question below:
Reviewer's feedback is highlighted in blue text and our responses are in green.

REVIEWER COMMENTS

Reviewer #1 (Remarks to the Author):
Reviewer Comment: I thank the reviewers for a highly responsive revision that improves the quality of data and analyses. Indeed, I think the fundamentals of this new technique are demonstrated robustly but I remain cautious about the claimed molecular distinction of patients vs. controls based on an enlarged but still relatively small set of independent samples. The field has experienced such strong initial claims many times only to learn later that the situation was more nuanced.
Response: Dear Reviewer#1, we are highly appreciative of your positive comments and critical feedback. Your feedback has greatly improved the quality of this manuscript. As suggested, we have now made following modifications in the main manuscript:

- We agree with reviewer's comment regarding "field has experienced such strong initial claims" and their subsequent impact. As suggested, the "Abstract" now explicitly says:
"Collectively, our study provides a proof-of-concept "
"they require validation in larger, blinded clinical trials."
- We have added new text in the "Limitations" section:
"Although the sample size is adequate for discovery, more work needs to be done regarding influence of race, gender, age, stage of AD, and the influence of uncharacterized co-morbidities variably present in the autopsy samples."
- The error in PMI data has been fixed in the "Extended data. Table 2. Patient sample information."
- As suggested, we conducted a new variance partition analyses keeping PMI as a confounder. The figure is shown below (Response Figure 1) and updated in main manuscript "Extended data Figure 10".
- New additions are highlighted in the revised manuscript and clean copy has been provided for your convenience.

1. My suggestion is to make clear that although the sample size is adequate for discovery, much more work needs to be done regarding influence of race, gender, age, stage of Alzheimer's disease, and the influence of uncharacterized co-morbidities variably present in the autopsy samples. The potential impacts of confounding by underlying disease that promoted brain biopsy and by general anesthesia also remain unknown for biopsy samples.
Response: We agree with the reviewer and appreciate your commitment to rigor of this publication.
As suggested, we have added a clear sentence in the abstract and limitations section.

"Abstract" now includes:
"Collectively, our study provides a proof-of-concept that the transcriptome of peripheral blood extracellular vesicles & particles (EVPs) carries disease-specific information in AD with potential applications as a minimally invasive diagnostic tool, although they require validation in larger, blinded clinical trials."

We have added new text in the "Limitations" section:

"Although the sample size is adequate for discovery, more work needs to be done regarding influence of race, gender, age, stage of AD, and the influence of uncharacterized co-morbidities variably present in the autopsy samples."

2. I suspect that there is an error in the supplementary table that describes characteristics of people who made brain donations. Post-mortem interval for the autopsy samples from one source ranges from 241 min to 1750 min and from a second source ranges from 4 min to 20 min. I expect that the units are incorrect for tissue from the second source and that PMI is 4 hr to 20 hr rather than min. Converting PMI from the first tissue source's data to hr then has their PMI ranging from 4 to 29 hr, conveniently overlapping between the two sources if my assumption is correct. If I am wrong, then there is a major systematic difference in the autopsy specimens from different sources.
Thanks for noticing this error. We have now presented all PMI data in minutes. The error in PMI data has been fixed in the "Extended data. Table 2". Patient sample information.

3. With this sample size and variation in PMI, I suggest that the authors conduct supplemental analyses on the effect of PMI on the major outcomes and report these in the text. I recognize that the authors have done variance analyses but these PMIs are broad and some longer than is typical for RNA-based detection.
We thank Reviewer for this very important point. As suggested, we conducted a new variance partition analyses. Here, we included PMI as a confounder in our variance partition analyses. PMI contributed to <3% total variance in our study.

This figure has now been added to the manuscript as "Extended data Figure 10." And reported in main manuscript.

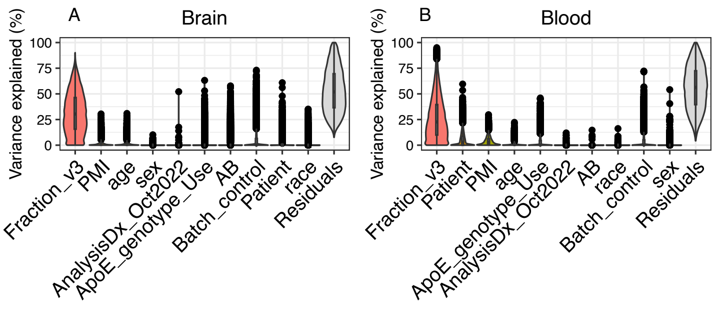

Response Figure 1. PMI as a confounding factor.

The field has experienced such strong initial claims many times only to learn later that the situation was more nuanced.
We agree and acknowledge this very important point raised by Reviewer 1. As a new investigator, the corresponding author is truly inspired by Reviewer1's feedback and commitment to rigor and reproducibility. The corresponding author has learned a great deal and look forward to practice this feedback in their own career. Thank you!

Reviewer #2 (Remarks to the Author):

The authors have addressed all of my comments and concerns, and the revised manuscript has improved accordingly. I have no further comments.
We would like to thank the reviewer #2 for their valuable time and critical feedback, which has improved the overall quality of our manuscript. Thank you!

Reviewer #3 (Remarks to the Author):

Reviewer Comment: In this manuscript, the authors show that a previously overlooked class of sub-50nm extracellular nanoparticles (small EPs), serves as a more effective non-invasive biomarker for Alzheimer's Disease (AD) than traditional small extracellular vesicles (sEVs). The researchers identified these nanoparticles in both human brain tissue and blood. Notably, they lack the protein hallmarks typically found in exosomes and sEVs but are highly enriched with brain-specific markers. In addition, RNA associated with small EPs can discriminate AD patients and healthy controls. Using single-nucleus RNA sequencing deconvolution, the authors mapped the origins of these particles and associate small EVs with RNA from oligodendrocytes and astrocytes and small EPs with brain-endothelial cells, critical components of the blood-brain barrier.

Response: Dear Reviewer#3, thank you for your constructive comments and valuable time spent to provide important feedback, which has helped us improve our manuscript. Additionally, we are highly appreciative of the Reviewer for recognizing the importance of our study, small EPs, and providing encouraging remarks. Also, we acknowledge Reviewer's concern about the contamination from intracellular vesicles during the isolation of brain-derived EVs/EPs. Additionally, your comments regarding the presence of microglia and exomeres markers has greatly helped us characterize the EVPs and develop new insights, which has now been added in the conclusion, methods, and limitations section. We assure you that we are dedicated to improving our empirical evidence and have conducted additional experiments to support our conclusion or adding limitations of the study. As suggested, we have now made following modifications in the main manuscript and included new complementary data in support of conclusions:

- Cellular debris contaminants: This is an important issue and a matter of intense debate in the EV community. As suggested, we conducted a new set of experiment and tested for the cellular debris and intracellular contaminants in the brain and blood EVP isolations (discussed below, new results presented in Response Fig. 2).
- TEM: We have added a new transmission electron micrograph (TEM) of small EVs derived from blood (Response Fig. 3).
- RNA normalization addressed: We agree that RNA normalization is a critical first step for interpreting differences in cargo composition. As suggested, we have discussed this in detail in the "Methods" section also presented below for your convenience.
- Sub-fraction by size: Reasoning for our focus on three EVP subfractions has been discussed in detail and new data has been included (Response Fig. 4).
- Reproducibility in different cell types: To ensure that our EVP isolation is rigorous and reproducible across cell types and biofluids, we have presented a new data showing a reproducible separation of EVPs in *in vitro* cell models (Response Fig. 5).
- Microglial markers presented: As suggested, we curated a strict list of transcripts considered highly specific to resident microglia. We show microglia-associated RNAs in different EVPs (Response Fig. 6).
- Exomere markers included: As suggested, we conducted a new bioinformatic analysis of brain and blood-derived EVPs' proteome, and compared with exomere markers as presented in Zhang Lyden et al. 2018 (Response Fig. 7).
- Consistent color scheme: We have made concerted effort to keep a consistent color-scheme. In all figures large EVs are colored "Blue", small EVs are "Red", and small EPs are "green".
- New additions are highlighted in the revised manuscript and a clean copy has been provided for your convenience.

Below we address specific comments provided by the Reviewer.
Specific Comments:
1) It is unclear how the authors excluded contamination from intracellular vesicles during the isolation of brain-derived EVs/EPs. Given the complexity of brain tissue, more detail is needed on how vesicles released extracellularly were distinguished from intracellular vesicles originating from disrupted cells. Please clarify the strategy and provide evidence that intracellular contamination is minimal.
Thanks for bringing the significant point of cellular contamination in EV isolations. We agree with the Reviewer that intracellular contamination must be address, especially when isolating EVs from the brain tissue. Furthermore, we acknowledge that this is an important issue and a matter of intense debate in the EV community. To address this, we have adopted following different approaches in our methodology. We discuss these in detail below:

1) Use of fresh human brain tissue: Flash frozen human brain induces cells death and apoptosis[1]. Recently, our team developed a comprehensive protocol to isolate viable cells from the fresh human brain tissue (Kosoy, Roussos* et al. *Nature Genetics*, 2022)[2]. Prior to starting the current project, the protocol was established on fresh brain tissue from a total of 150 unique donors derived from biopsies (n=27) and autopsies (n=123)[2]. A byproduct of this is, left over human brain tissue supernatant from fresh human brain, which is used to isolate human brain EVPs presented in this study. Although this approach ensures fresh EVPs from unfrozen brain tissue derived viable cells, we agree that this strategy does not completely eliminate the likelihood of cellular contamination (cellular stress, rupture, blebbing) in the EVP isolation.

2) Testing for the cellular contaminants in the EVP isolations: We thanks the reviewer for this important point, as we learned that there indeed is some cellular stress during EVP isolation. We discuss this below in details:
To test if fresh brain cells undergo cellular stress or damage during EVP isolation, we tested for following markers:
a) Cytochrome C in brain-derived samples is generally interpreted as a marker of mitochondrial stress and apoptosis, reflecting neuronal injury or metabolic failure. Cytochrome C is also mentioned in MISEV2023[3] as a transmembrane protein associated with intracellular compartments other than plasma membrane or endosomes.
b) HMGB1 is a nonhistone nuclear chromatin-binding protein, is best interpreted as a DAMP marker of cellular stress, necrosis, and inflammation, particularly relevant for brain injury and neurodegenerative processes.
Analysis of selected analytes: Here, we conducted a Luminex assay (a type of immunoassay that precisely measures multiple analytes in one sample) on EVPs. We found that while the nuclear protein HMGB1 was below the detection limits in the blood and brain-derived EVPs, Cytochrome C was elevated in brain-derived EVPs (Response Fig. 2A-B). These results indicate minimum nuclear contamination but likely mitochondrial stress or neuronal injury during the EVP isolation from the brain tissue.

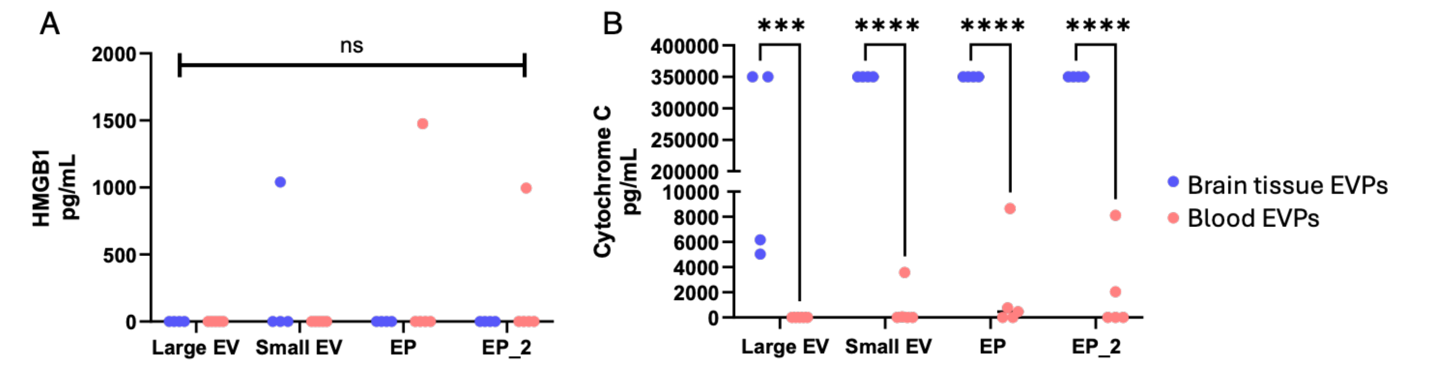

Response Figure 2. Testing for intracellular contamination in brain and blood derived EVPs. A) HMGB, a nuclear protein was below the detection limits in both the brain and blood-derived EVPs. **B**) Cytochrome C a marker of mitochondrial stress and apoptosis, is elevated in brain-derived EVPs.

After this observation and suggestion by the Reviewer #3, we have added following text in the "Limitations" section of the main manuscript, also highlighted below for your convenience:
"While care was taken to gently dissociate fresh brain tissue, it is difficult to completely eliminate the possibility of cellular contamination arising from cellular stress, rupture, or membrane blebbing during tissue processing. Consequently, the presence of intracellular components, including intracellular vesicles, in the EVP isolation from brain tissue cannot be entirely excluded."

We have added following text in main manuscript "Results" section:
"We found that while the nuclear protein HMGB1 was below the detection limits in the blood and brain-derived EVPs, Cytochrome C was elevated in brain-derived EVPs."

As suggested by the reviewer, following text has been added to the "Methods" section:
"We followed the following protocol, which has also been published in our recent publication[2]. Fresh autopsy and biopsy tissue specimens were processed using the Adult Brain Dissociation Kit (Miltenyi Biotech Cat# 130107677), according to manufacturer's instructions. Following demyelination (Miltenyi de myelination kit, Miltenyi Biotech, Cat# 130096733) cells were incubated 10 in antibody (CD45: BD Pharmingen, Clone HI30 and CD11b: BD Pharmingen, Clone ICRF44) at 1:500 for 1 hour in the dark at 4 ˚C with end-over-end rotation. RNase inhibitors (Takara Bio) were used throughout the cell prep. Prior to fluorescence activated cell sorting (FACS), DAPI (Thermoscientific) was added at 1:1000 to facilitate the separation of live from dead cells. Viable (DAPI negative) CD45/CD11b positive cells were isolated by FACS using a FACSAria flow 15 cytometer (BD Biosciences). Following FACS, cell concentrations and viability were confirmed using a Countess automated cell counter (Life technologies)."

2) The EM images are generally unfocused and do not convincingly support the reported size distributions. In particular, the images of blood-derived EVs are of very low informative value and do not add meaningful support to the conclusions. Higher-quality EM data would be necessary.
We appreciate the Reviewer for making this important observation. We have added a new transmission electron micrograph of small EVs derived from blood (Response Fig. 3B). We acknowledge that TEM is merely for a qualitative analysis of EVPs in this study. The size distribution of EVPs is measured using dynamic light scattering (DLS) from 10 human brain and blood EVP samples (Response Fig. 3B and Fig. 1N, main manuscript) We hope that these images are satisfactory to the reviewer.

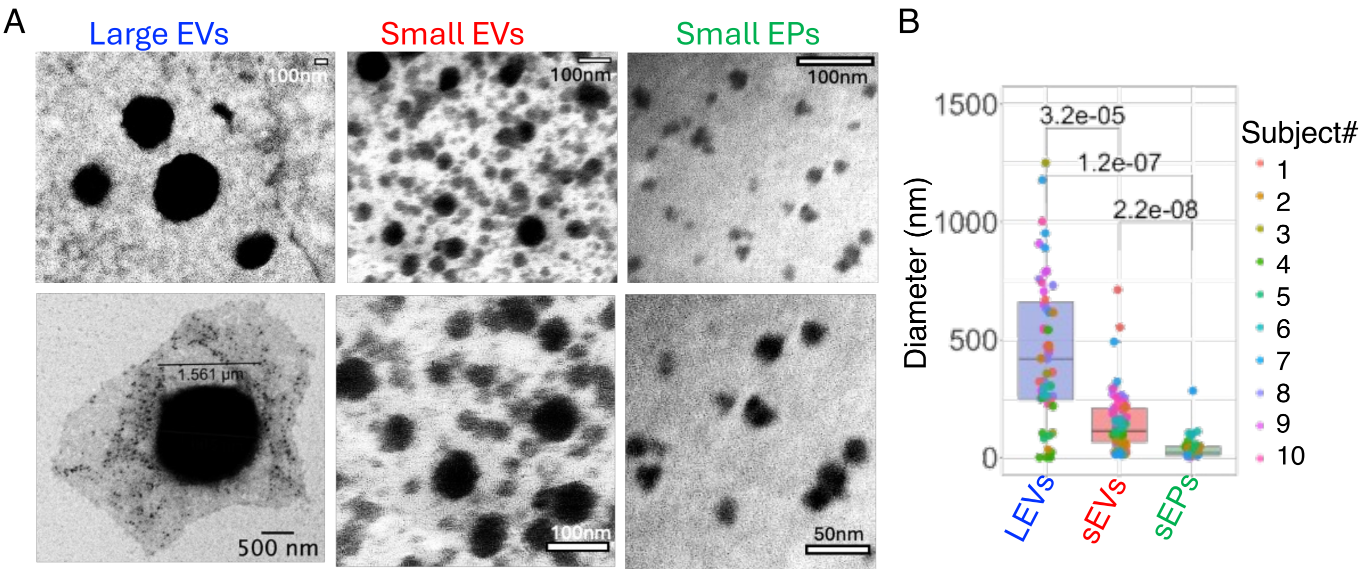

Response Figure 3. **A**. Transmission electron micrograph (TEM) of EVPs from blood. **B**. Reproducible ($P < 0.00005$) separation of large EVs, small EVs, and small EPs in 10 human subjects (5 AD and 5 controls). The dots are colored by each individual subject. Significant P-value is observed for all EVPs. P-values were calculated using a two-sided Welch's t-test, which compares group means while accounting for unequal variances.

3) Regarding the analysis of total RNA content in each EV/EP population, it is unclear how normalization was performed. Was RNA normalized to EV number, EV size, total protein, or another parameter? This is critical for interpreting differences in cargo composition.

Thaks for this important point. We agree that RNA normalization is a critical first step for interpreting differences in cargo composition. In our previous publications[4-6], we have observed variability with RNA analyses and established a foundation of robust protocols, which helped us conduct this study.

We take several steps to ensure that the interpretation of cargo differences in robust and reproducible.

1) Careful quantification of RNA and cDNA in <u>library preparation</u>: Before the cDNA library prep, total RNA is carefully quantified and same amount of total RNA concentration is used for cDNA preparation. As controls, no RNA negative control, and control RNA miR163s are used. Additionally, universal human reference RNA (UHRR) control is used. Finally, the cDNA is normalized across samples and sequenced. Next-generation RNA sequencing is performed and read alignment to human genome is compared with negative and positive controls (typically in our samples negative controls yield <3% genome alignment). These steps make sure that similar amounts of RNA are present in each samples, allowing to compare the quantity of RNA across sample for a specific gene or non-coding region.

Above text has been added to the "Methods" section.

2) Normalization of <u>RNA-seq count data</u>: Raw counts are first converted to log-counts per million (logCPM) using the voom method in limma. Before this transformation, library sizes are normalized to account for differences in sequencing depth across samples, commonly using the TMM (trimmed mean of M-values) method implemented in edgeR. TMM scales samples so that the majority of genes have similar expression distributions across libraries. The voom function then models the mean–variance relationship of logCPM values and assigns precision weights to each observation, allowing the RNA-seq data to be analyzed with limma's linear modeling framework. Thus, normalization in a limma RNA-seq pipeline generally involves library size normalization, transformation to logCPM, and variance modeling via voom, rather than a single internal normalization procedure.

Above text has been added to the "Methods" section.

3) Bootstrapping to enhances the accuracy of differential analysis and the gene expression: We applied bootstrapping to enhances the accuracy of differential analysis and the gene expression results providing a non-parametric, empirical approach to estimate variability, standard errors, confidence intervals, and p-values through repeated resampling with replacement from the original data. This reduces reliance on potentially mis specified parametric assumptions, leading to more robust and well-calibrated inferences, particularly in low-replicate scenarios or heterogeneous datasets common in omics. Figures 6D–E presents the top differentially expressed genes as a mean of gene expression across all subjects, identified within each specified comparison. On the other hand, Figure 6G provides an integrated summary of established brain-specific genes that are both significantly altered and shared across the overlapping signatures. These analyses encompass multiple parallel comparisons, including EP-, small EV–, and large EV–derived blood samples, evaluated across three independent cohorts.

The criteria for gene selection in Figure 6G has now been described in "Methods" section and below for your convenience.

"*We applied bootstrapping to enhances the accuracy of differential analysis and the gene expression results providing a non-parametric, empirical approach to estimate variability, standard errors, confidence intervals, and p-values through repeated resampling with replacement from the original data. This reduces reliance on potentially mis specified parametric assumptions, leading to more robust and well-calibrated inferences, particularly in low-replicate scenarios or heterogeneous datasets common in omics. For differentials between batches (technical groupings like sequencing runs) and cohorts (biological groupings like patient populations or studies), we applied a specialized bootstrapping strategy: stratified resampling within batches and hierarchical resampling to respect nested structures (samples within donors or cohorts), to separate technical noise from biological signal. This improves reproducibility, controls false discovery rates more effectively, reduces bias from unmodeled batch effects or cohort heterogeneity, and increases statistical power and stability when comparing results across technical batches or biological cohorts, often yielding more reliable differential calls than standard parametric methods alone*."

4) The manuscript emphasizes fundamental cargo differences between EV/EP classes, potentially reflecting distinct biogenetic pathways. However, further sub-fractionation by size could reveal additional heterogeneity, as EV size may correspond to distinct functional subpopulations depending on cell type and biogenesis mechanisms.

Thanks for this suggestion. We agree that further sub-fractionation by size could reveal additional heterogeneity.

In our study, we focused on three subfractions because we found three distinct subpopulations reproducibly present in human brain tissues (n = 26), blood (n = 26), and cell culture model (new data, discussed below in Response Fig. 5). We found that three EVP subpopulation have distinct sizes, scattering patterns, zeta potentials and several biophysical properties (Response Fig. 4 and Fig 1 of main manuscript). To further ensure this reproducibility, we have conducted a comprehensive quality control analysis of separate EVPs to characterize their size, shape, morphology, scattering patterns, surface charges, proteins, RNAs, and canonical markers (Fig 1 of the main manuscript). Based on these distinct features, we classified EVPs into three subpopulations — large EVs, small EVs, and small EPs.

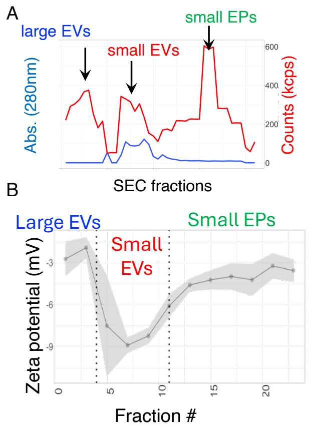

Response Figure 4. Analysis of EVP subpopulations. Scattering patterns (A) Zeta potential (B) from large EVs, small EVs, and small EPs.

Reproducible separation of EVPs in other cell models: While this data is beyond the scope of current manuscript, we wish to share with the reviewer that our methodology is reproduced in other biofluids, disease models, and cell culture derived EVPs. In another ongoing project in the lab, two distinct human embryonic kidney (HEK) cell models (wild type and mutant) were cultured independently, and EVPs were isolated using SECrifuge methodology (Response fig. 5). The goal of this project is to isolate small EVs and small EPs. We were able to apply our protocols to cell cultures and were able to obtain similar results as presented in this manuscript.

Finally, we wish to share that we (the corresponding author and co-authors) were recently awarded a new NIH R01 (R01CA296581) grant, where our SECrifuge methodology will be used for EVP isolation
 from >100 human subject's blood serum. Consequently, we are continuing to test and improve our SECrifuge methodology. In summary, our new experiments reassure the reproducible isolation and characterization of EVPs isolated using SECrifuge and comparison with existing technologies.

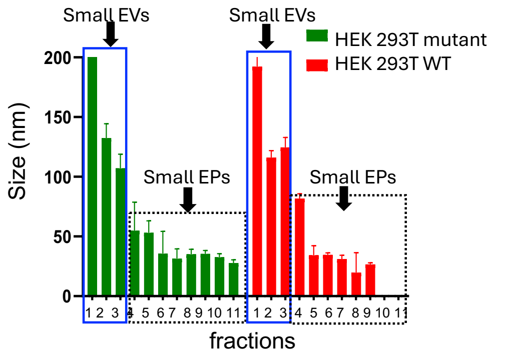

Response Figure 5. EVP isolation in other cell models. Two distinct cell models were cultured independently, and EVPs were isolated using SECrifuge.

5) Do the brain-specific transcript signatures include microglial markers? This should be clarified, as microglia represent a major brain-resident cell population and could significantly influence the interpretation of brain-derived EV cargo.

We thank the reviewer for this important point. Your feedback regarding the presence of microglia markers has greatly helped us to further characterize the EVPs and develop new insights, which have now been added in the conclusion, methods, and limitations section.

As suggested, we curated a new strict list of transcripts considered highly specific to resident microglia with minimal expression in neurons, astrocytes, oligodendrocytes, and infiltrating monocytes/macrophages under homeostatic conditions (Response Fig. 6). We found that different EVPs were enriched in different microglial RNA markers. This finding is exciting as it adds new valuable information for AD and EVP community and has been added to the main manuscript (Extended data. Figure 5).

For our single-cell study (Fig. 5 of the main manuscript), the brain-specific transcript signatures were curated from the Human Protein Atlas, GTEx Portal, and the Allen Brain Atlas, and were defined based on brain-enriched expression rather than restriction to a single cell type. When examined at single-cell resolution these transcripts were detected across major brain-resident populations, including neurons, astrocytes, oligodendrocytes, and microglia. Thus, the signature reflects broadly brain-enriched transcripts and does not represent a microglia-specific gene set.

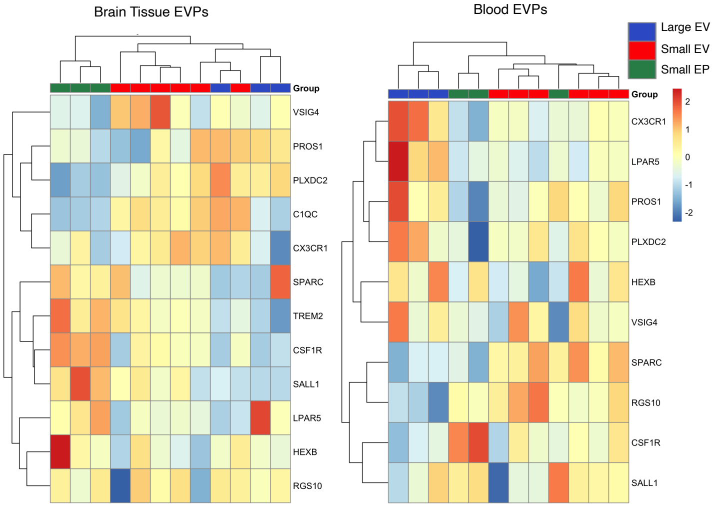

Response Figure 6. Microglial RNA signature and its presence in brain and blood-derived EVPs.

We have clarified this in the revised manuscript to ensure accurate interpretation of the cellular contributions to EV cargo.

"The snRNA-seq data revealed eight distinct cellular clusters, including astrocytes, excitatory and inhibitory neurons, endothelial cells, immune cells (predominantly microglia), mural cells, oligodendrocyte progenitor cells, and oligodendrocytes (Fig. 5A). To infer cellular origin, we assessed the distribution of our curated brain-enriched transcript set across clusters (Fig. 5B, C). This gene set includes markers expressed across major brain-resident populations, including neuronal, astrocytic, oligodendrocytes, and microglial markers, allowing us to evaluate relative cellular contributions to EV cargo."

A new figure in main manuscript (Extended data. Figure 5C, D) entitled "Microglial RNA signature and its presence in brain and blood-derived EVPs." has been added.

6) Markers typically associated with exomeres are missing for the sEP population. Please clarify whether these were assessed and, if not, discuss this limitation.
Thank you for this suggestion. Similarly, this feedback has greatly helped us to further characterize the EVPs and develop new insights, which have now been added in the conclusion, methods, and limitations section.

As suggested, we conducted a new analysis, where we curated exomere markers as published by Zhang Lyden et al. 2018[7]. We found that most of the representative exomeres protein markers were enriched in small EVs (response Fig. 7). The small EPs carried a unique set of proteins (response Fig. 7), which are not reported in exomeres. We also noticed that the original publication that defined exomeres, conducted experiments in different representative *in vitro* cell lines (B16-F10, AsPC-1, Pan02, MDA-MB-231-4175 and 4T1). In contrast, our study was conducted in human biofluids. Human biofluids add additional complexity to such experiments due to an abundance of lipoproteins ($\approx 10^{16}$ lipoproteins/mL), and NVEPs present in blood, which are generally less pronounced in most cell cultures[8].
Above text has been added to the "Conclusion" section of the main manuscript.

Overall, we appreciate this point by the reviewer because this led us to carry out additional analyses and comparison of our small EPs, which helped us to find unique proteins associated with small EP from brain and blood. Thanks to this feedback, we were able to report new set of EP-associated proteins.

The original figure (Figure 2D, E) has now been updated and also shown below for your convenience.

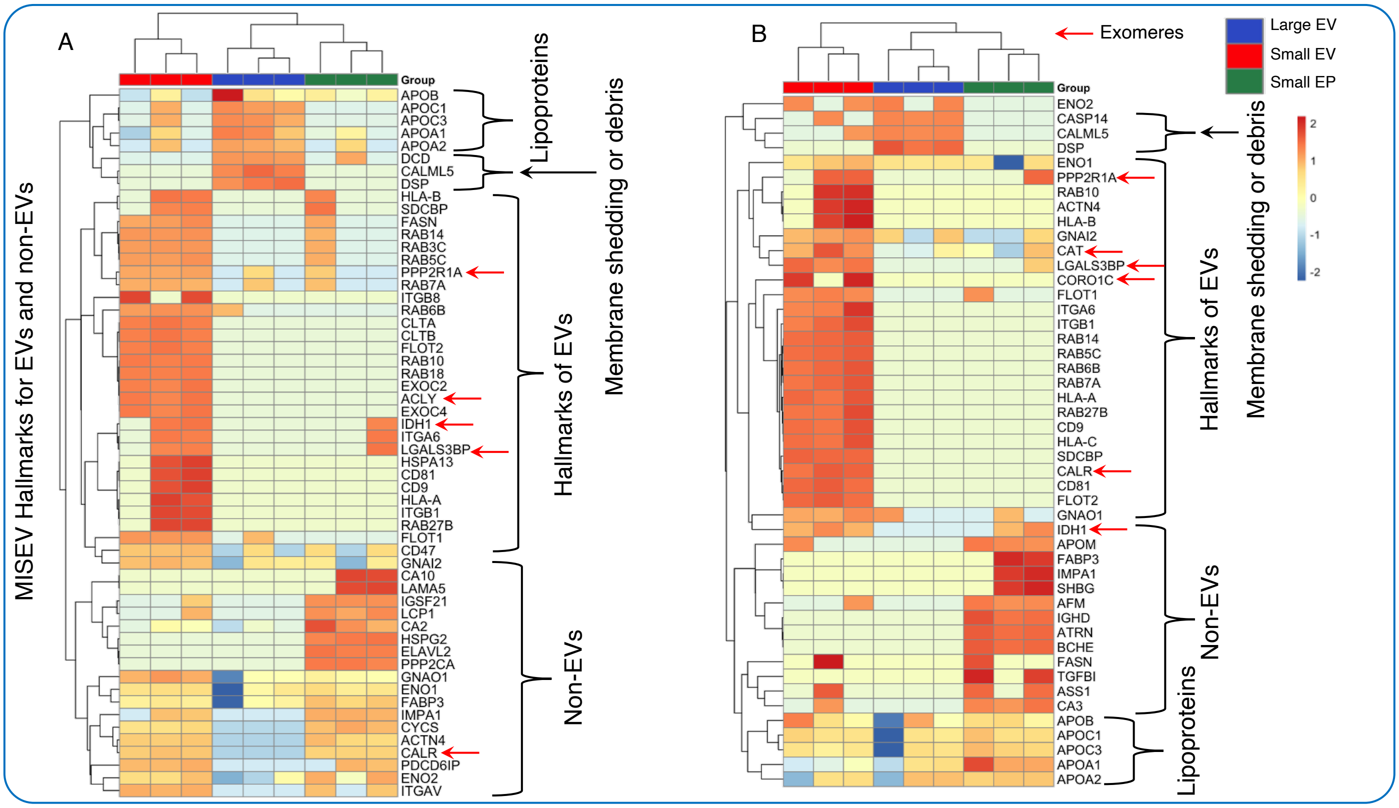

Response Figure 7. Characterization of biomolecular cargo associated with EVPs from brain (A) and blood (B). Exomeres associated protein markers are shown with red arrow.

7) Extended Data Fig. 2A–F does not appear essential to support the main conclusions and could potentially be removed.

We thank reviewer for this suggestion. If it is okay with the reviewer, we would like to keep this figure for data transparency reasons.

8) In Fig. 2D, CD63 is missing. Please clarify whether CD63 was assessed.

Thanks for noticing this important canonical marker for EVs. In our study, CD63 was not significantly detected in the proteomic data from EVPs. We have made a note in the main manuscript "Results" section, entitled "Characterization of MISEV-recommended biomolecular cargo associated with heterogeneous EVPs"

9) The color codes used in Figures 1–3 are confusing, not only across different figures but even within the same panel. A consistent color scheme would greatly improve readability.

We appreciate Reviewer for this suggestion. We have made concerted effort to keep a consistent color-scheme. In all figures, large EVs are colored "Blue", small EVs are "Red", and small EPs are "green".

Finally, we are thankful to the Reviewer for their time, several positive comments, and important feedback that has made this manuscript much stronger. The new data presented here enhances the reproducibility and robustness of our study. Your feedback has greatly helped us to further characterize the EVPs and develop new insights, which have now been added in the conclusion, methods, and limitations section.

We are hopeful that our response is satisfactory and that this manuscript will be an excellent resource for the EVP and AD community. Thank you!

1. Martinez-Madrid, B., Camboni, A., Dolmans, M.M., Nottola, S., Van Langendonckt, A., and Donnez, J. (2007). Apoptosis and ultrastructural assessment after cryopreservation of whole human ovaries with their vascular pedicle. Fertil Steril *87*, 1153-1165. 10.1016/j.fertnstert.2006.11.019.
2. Kosoy, R., Fullard, J.F., Zeng, B., Bendl, J., Dong, P., Rahman, S., Kleopoulos, S.P., Shao, Z., Girdhar, K., Humphrey, J., et al. (2022). Genetics of the human microglia regulome refines Alzheimer's disease risk loci. Nat Genet *54*, 1145-1154. 10.1038/s41588-022-01149-1.
3. Welsh, J.A., Goberdhan, D.C.I., O'Driscoll, L., Buzas, E.I., Blenkiron, C., Bussolati, B., Cai, H., Di Vizio, D., Driedonks, T.A.P., Erdbrugger, U., et al. (2024). Minimal information for studies of extracellular vesicles (MISEV2023): From basic to advanced approaches. J Extracell Vesicles *13*, e12404. 10.1002/jev2.12404.
4. Chen, T.-Y., Gonzalez-Kozlova, E., Soleymani, T., La Salvia, S., Kyprianou, N., Sahoo, S., Tewari, A.K., Cordon-Cardo, C., Stolovitzky, G., and Dogra, N. (2022). Extracellular vesicles carry distinct proteo-transcriptomic signatures that are different from their cancer cell of origin. iScience *25*. 10.1016/j.isci.2022.104414.
5. Dogra, N., Chen, T.Y., Gonzalez-Kozlova, E., Miceli, R., Cordon-Cardo, C., Tewari, A.K., Losic, B., and Stolovitzky, G. (2024). Extracellular vesicles carry transcriptional 'dark matter' revealing tissue-specific information. J Extracell Vesicles *13*, e12481. 10.1002/jev2.12481.
6. Miceli, R.T., Chen, T.Y., Nose, Y., Tichkule, S., Brown, B., Fullard, J.F., Saulsbury, M.D., Heyliger, S.O., Gnjatic, S., Kyprianou, N., et al. (2024). Extracellular vesicles, RNA sequencing, and bioinformatic analyses: Challenges, solutions, and recommendations. J Extracell Vesicles *13*, e70005. 10.1002/jev2.70005.
7. Zhang, H., Freitas, D., Kim, H.S., Fabijanic, K., Li, Z., Chen, H., Mark, M.T., Molina, H., Martin, A.B., Bojmar, L., et al. (2018). Identification of distinct nanoparticles and subsets of extracellular vesicles by asymmetric flow field-flow fractionation. Nat Cell Biol *20*, 332-343. 10.1038/s41556-018-0040-4.
8. Simonsen, J.B. (2017). What Are We Looking At? Extracellular Vesicles, Lipoproteins, or Both? Circ Res *121*, 920-922. 10.1161/CIRCRESAHA.117.311767.
